# Smart Nano-Antibiotics: AI-Guided Stimuli-Responsive Nanoplatforms for Precision Antimicrobial Therapy

**DOI:** 10.3390/antibiotics15070638

**Published:** 2026-06-26

**Authors:** Nargish Parvin, Keunhwan Park, Jae Hak Jung, Tapas Kumar Mandal

**Affiliations:** 1 Department of Mechanical Engineering, Gachon University, Seongnam 13120, Republic of Korea; nargish.parvin@gmail.com; 2 PiQuant Co., Ltd., Seoul 04323, Republic of Korea; 3 School of Chemical Engineering, Yeungnam University, Gyeongsan 38541, Republic of Korea

**Keywords:** smart nano-antibiotics, artificial intelligence, stimuli-responsive nanocarriers, targeted drug delivery, antimicrobial resistance

## Abstract

The rapid rise of antimicrobial resistance (AMR) has created an urgent need for innovative therapeutic strategies beyond conventional antibiotics. Smart nano-antibiotics have emerged as advanced antimicrobial systems capable of improving drug delivery, enhancing pathogen targeting, overcoming biofilm-associated resistance, and reducing systemic toxicity. This review discusses recent progress in stimuli-responsive nanoplatforms, including pH-responsive, enzyme-responsive, temperature-sensitive, and redox-activated systems for precision antimicrobial therapy. The role of artificial intelligence in nanomaterial design, toxicity prediction, drug release optimization, and personalized treatment development is also critically examined. Furthermore, the review highlights targeted antimicrobial delivery, multifunctional nano-drug combination systems, biosensor integration, and autonomous AI-driven therapeutic platforms for combating multidrug-resistant infections. Current challenges related to toxicity, regulatory limitations, scalability, and AI data reliability are discussed alongside emerging clinical and industrial developments. Smart nano-antibiotics represent a promising next-generation approach for improving precision antimicrobial therapy and addressing the growing global burden of antimicrobial resistance.

## 1. Introduction

Antimicrobial resistance (AMR) has emerged as one of the most serious global health challenges of the twenty-first century, affecting clinical medicine, public health systems, food security, and economic sustainability [1,2]. The increasing prevalence of multidrug-resistant (MDR), extensively drug-resistant (XDR), and pan drug-resistant pathogens has significantly reduced the effectiveness of many frontline antibiotics, leading to prolonged hospitalization, recurrent infections, increased mortality, and rising healthcare costs [3,4,5]. Particularly concerning is the continued spread of resistant ESKAPE pathogens, which are responsible for a substantial proportion of difficult-to-treat healthcare-associated infections [6]. The widespread misuse and overuse of antibiotics in human health, veterinary practice, and agriculture have further accelerated the dissemination of resistance genes across clinical and environmental settings. Despite decades of antibiotic development, the discovery pipeline for new antimicrobial agents has slowed considerably. Conventional antibiotics typically target a limited number of bacterial processes, including cell wall synthesis, protein synthesis, nucleic acid replication, and metabolic pathways [7]. Although several new antimicrobial scaffolds and derivative compounds continue to emerge, the pace of antibiotic development remains insufficient to keep pace with the global spread of antimicrobial resistance [8]. However, microorganisms rapidly evolve resistance through enzymatic degradation, target modification, efflux pumps, altered membrane permeability, and biofilm formation. These limitations, together with poor site-specific delivery and uncontrolled drug distribution, have reduced the effectiveness of traditional antimicrobial therapies against persistent infections [9,10]. Consequently, there is an urgent need for innovative therapeutic strategies capable of overcoming biological resistance barriers while improving treatment efficiency. Nanotechnology has emerged as a promising platform for next-generation antimicrobial therapy because of its unique physicochemical properties and multifunctional capabilities. Nanomaterials possess tunable surface chemistry, high surface-area-to-volume ratios, and customizable architectures that enhance interactions with microbial cells [11]. Various nanoplatforms, including metallic nanoparticles, polymeric nanocarriers, lipid-based nanoparticles, mesoporous silica systems, dendrimers, carbon nanostructures, and hybrid nanocomposites, have demonstrated encouraging antimicrobial activity against resistant pathogens. Unlike conventional antibiotics that generally act through a single mechanism, nano-antibiotics can exert multiple antimicrobial effects, including membrane disruption, oxidative stress induction, intracellular damage, and interference with microbial metabolism [12]. These properties have stimulated significant interest in nanotechnology-based approaches for combating antimicrobial resistance. A major advancement in this field is the development of stimuli-responsive or “smart” nano-antibiotic systems capable of releasing therapeutic agents in response to specific biological or external triggers [13,14,15]. Such systems can be activated by pH changes, enzymatic activity, redox imbalance, temperature variation, hypoxia, or externally applied stimuli such as light, magnetic fields, and ultrasound [16,17]. By regulating drug release according to local environmental conditions, smart nanoplatforms offer greater control over therapeutic activation than conventional formulations. Alongside advances in nanotechnology, artificial intelligence (AI) has emerged as a powerful computational tool for pharmaceutical research and antimicrobial discovery [18,19]. AI-driven approaches, including machine learning, deep learning, neural networks, and predictive modeling, can analyze complex biological datasets, identify novel antimicrobial candidates, predict drug–target interactions, and optimize nanomaterial design. In antimicrobial nanomedicine, AI is increasingly applied to predict nanoparticle behavior, optimize physicochemical properties, evaluate toxicity profiles, and improve the design of stimuli-responsive delivery systems [20]. These capabilities have the potential to accelerate development timelines and enhance the clinical translation of smart nano-antibiotic technologies. The integration of AI with stimuli-responsive nanoplatforms has therefore created a promising framework for next-generation precision antimicrobial therapy. However, important challenges remain, including nanoparticle toxicity, immune interactions, manufacturing scalability, regulatory uncertainty, data standardization, and clinical reproducibility [21]. To address these issues, this review examines the fundamental principles of smart nano-antibiotics, stimuli-responsive delivery systems, AI-assisted nanomaterial design, targeted antimicrobial strategies, translational barriers, and future opportunities for intelligent antimicrobial therapies aimed at combating AMR. Here Table 1 compares conventional antibiotics with smart nano-antibiotic systems and highlights the major advantages of nanotechnology-based antimicrobial therapy. The table demonstrates how smart nanoplatforms improve targeting efficiency, controlled drug release, biofilm penetration, and therapeutic precision while reducing resistance development and systemic toxicity. The comparison emphasizes the potential of smart nano-antibiotics as advanced alternatives for combating multidrug-resistant infections [13]. A comprehensive understanding of the design principles, mechanistic behavior, AI integration strategies, and translational barriers associated with smart nano-antibiotics is therefore essential for advancing this rapidly evolving field.

**Figure 1:**
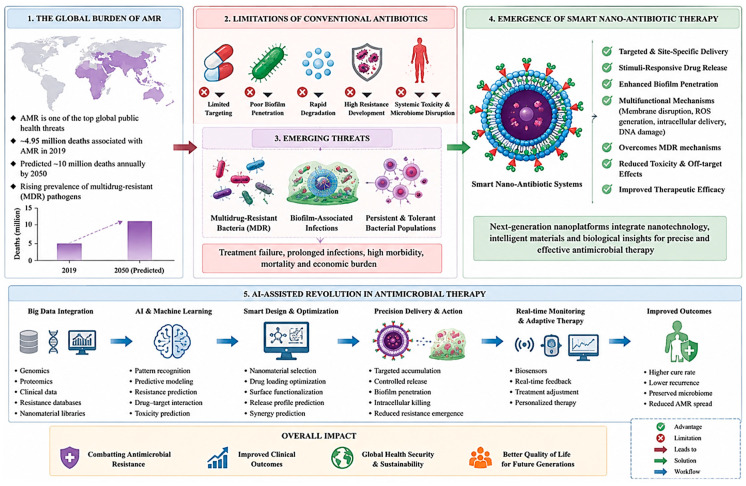
Overview of antimicrobial resistance challenges and the emerging role of AI-guided smart nano-antibiotics. The figure highlights the limitations of conventional antimicrobial therapy and illustrates how stimuli-responsive nanoplatforms integrated with artificial intelligence can support targeted drug delivery, controlled release, and improved therapeutic decision-making for resistant infections.

To address these challenges, the present review follows a structured framework that connects the major components of next-generation antimicrobial therapy. The discussion begins with the fundamental principles of nano-antibiotics and stimuli-responsive drug delivery systems, followed by the emerging role of artificial intelligence in nanomaterial design and therapeutic optimization. Subsequently, targeted antimicrobial strategies, advanced nanoplatforms, translational challenges, and clinical developments are critically examined. Finally, future opportunities for intelligent and personalized antimicrobial systems are highlighted within the broader context of combating antimicrobial resistance. Figure 1 illustrates the growing global burden of antimicrobial resistance (AMR) and highlights the limitations of conventional antibiotics, including poor targeting, limited biofilm penetration, rapid drug degradation, and increasing multidrug-resistant bacterial infections. So it further demonstrates how smart nano-antibiotic systems integrated with artificial intelligence can provide advanced solutions through targeted delivery, stimuli-responsive drug release, enhanced antimicrobial efficacy, biofilm disruption, and real-time therapeutic optimization. The schematic emphasizes the transition from traditional antimicrobial therapy toward intelligent precision nanomedicine for effective management of resistant infectious diseases.

### 1.1. Literature Search Methodology

This article was conducted as a narrative review focusing on recent advances in AI-guided smart nano-antibiotic systems for precision antimicrobial therapy. Relevant literature was collected from major scientific databases, including PubMed, Web of Science, Scopus, and Google Scholar. The search primarily covered publications from 2015 to 2025 using combinations of keywords such as “antimicrobial resistance”, “nano-antibiotics”, “smart nanocarriers”, “stimuli-responsive nanoparticles”, “artificial intelligence”, “machine learning”, “nanomedicine”, “biofilm infections”, and “precision antimicrobial therapy”. Peer-reviewed research articles, reviews, and selected clinical studies published in English were considered. Studies were selected based on their relevance to smart nano-antibiotic design, AI-assisted optimization, antimicrobial applications, translational challenges, and clinical development. Duplicate, unrelated, and insufficiently documented studies were excluded to ensure scientific relevance and quality.

### 1.2. Scope and Objectives of This Review

This review aims to provide a comprehensive and critical overview of AI-guided stimuli-responsive nano-antibiotic systems for precision antimicrobial therapy. The major objectives of this review are summarized below:

- To discuss the global burden and biological mechanisms associated with antimicrobial resistance.
- To critically analyze the limitations of conventional antibiotic therapies against resistant infections.
- To examine recent advances in antimicrobial nanotechnology and multifunctional nano-antibiotic platforms.
- To explore the design and mechanistic principles of stimuli-responsive antimicrobial nanocarriers.
- To evaluate the emerging role of artificial intelligence in antimicrobial drug discovery and nanoformulation optimization.
- To highlight the potential of AI-integrated smart nanoplatforms in precision and personalized antimicrobial therapy.
- To discuss current translational challenges, biosafety concerns, and future clinical perspectives associated with smart nano-antibiotics.

## 2. Fundamentals of Smart Nano-Antibiotics

Smart nano-antibiotics represent an advanced class of multifunctional antimicrobial systems that integrate nanotechnology, intelligent drug delivery mechanisms, and precision therapeutic engineering to overcome the growing limitations of conventional antibiotics. Unlike traditional antimicrobial agents, these nanoscale platforms possess tunable physicochemical properties, enhanced pathogen targeting capability, controlled drug release behavior, and improved bioavailability, enabling more efficient treatment of multidrug-resistant infections and biofilm-associated diseases. The fundamental design of smart nano-antibiotics involves diverse nanomaterials including metallic nanoparticles, polymeric carriers, lipid-based systems, and hybrid nanostructures that can interact dynamically with microbial cells and pathological microenvironments. In addition, these systems utilize multiple antimicrobial mechanisms such as membrane disruption, reactive oxygen species generation, intracellular delivery enhancement, and stimuli-responsive therapeutic activation. Understanding the classification, structural composition, antimicrobial mechanisms, and functional advantages of smart nano-antibiotic systems is therefore essential for developing next-generation precision antimicrobial therapies capable of improving therapeutic efficacy, minimizing toxicity, and reducing antimicrobial resistance progression.

### 2.1. Definition and Classification of Nano-Antibiotics

Nano-antibiotics represent an advanced class of antimicrobial systems in which nanoscale materials are engineered either to possess intrinsic antimicrobial properties or to function as carriers for controlled antibiotic delivery. Unlike conventional antibiotics that rely primarily on direct biochemical inhibition of specific microbial pathways, nano-antibiotics integrate nanoscale physicochemical interactions with therapeutic functionality, thereby enabling multimodal antimicrobial mechanisms [22]. These systems are generally designed within a particle size range of 1–100 nm, although larger Nano architectures may also exhibit nanoscale biological behavior depending on their surface properties and structural organization. The emergence of nano-antibiotics has significantly transformed the conceptual framework of antimicrobial therapy by introducing programmable, adaptive, and stimuli-responsive therapeutic systems capable of overcoming several resistance-associated limitations of traditional antibiotics [11]. The term “smart nano-antibiotics” specifically refers to engineered nanoplatforms capable of responding dynamically to biological or external stimuli while simultaneously performing targeted antimicrobial actions. Such systems are often integrated with environmental sensing capability, controlled drug release behavior, pathogen recognition properties, and multifunctional therapeutic responses [23]. Smart nano-antibiotics may selectively activate antimicrobial activity in response to acidic infection microenvironments, bacterial enzymes, reactive oxygen species (ROS), temperature changes, hypoxia, magnetic fields, ultrasound, or light irradiation [24]. This responsive functionality allows spatially and temporally regulated therapy, thereby minimizing systemic toxicity and improving therapeutic precision. In many advanced formulations, smart nano-antibiotics also incorporate imaging agents, biosensors, or AI-assisted predictive modules that further enhance treatment personalization and infection monitoring. Nano-antibiotics can be broadly classified according to their composition, functional mechanism, therapeutic role, and responsiveness characteristics. Based on therapeutic functionality, nano-antibiotics are generally divided into intrinsic antimicrobial nanoparticles and antibiotic-loaded nanocarriers [22]. Intrinsic antimicrobial nanoparticles directly exert bactericidal effects through physicochemical interactions with microbial cells. Examples include silver nanoparticles, zinc oxide nanoparticles, copper oxide nanoparticles, titanium dioxide nanoparticles, and graphene-derived nanostructures. These materials often induce membrane disruption, oxidative stress generation, protein denaturation, DNA damage, and metabolic dysfunction in microbial cells [25]. Because their antimicrobial activity involves simultaneous multi-target interactions, the probability of microorganisms developing rapid resistance against such systems is substantially reduced compared to single-target conventional antibiotics. In contrast, antibiotic-loaded nanocarriers primarily function as transport systems for delivering antimicrobial agents to infection sites with improved efficiency and controlled pharmacokinetics [26]. These carriers encapsulate, adsorb, or chemically conjugate antibiotics within nanoscale matrices, thereby enhancing drug solubility, stability, bioavailability, and tissue penetration. Controlled release properties further enable sustained therapeutic concentration while reducing dosing frequency and systemic side effects [27]. Nanocarrier systems have demonstrated particular effectiveness against intracellular pathogens and biofilm-associated infections where conventional antibiotic diffusion is severely restricted. Furthermore, targeted surface modification using ligands, antibodies, peptides, or aptamers allows selective recognition of infected tissues or microbial surfaces, thereby improving therapeutic specificity. From a compositional perspective, nano-antibiotics are commonly categorized into metallic nanoparticles, polymeric nanoparticles, lipid-based nanostructures, carbon-based nanomaterials, silica-based systems, dendrimers, and hybrid nanocomposites. Metallic nanoparticles possess strong intrinsic antimicrobial activity and are widely investigated for broad-spectrum infection control [28]. Polymeric nanoparticles provide exceptional flexibility in drug encapsulation, biodegradability, and stimuli-responsive engineering. Lipid-based nanosystems offer superior biocompatibility and membrane interaction properties, making them suitable for clinical translation. Carbon nanostructures such as graphene oxide, carbon nanotubes, and fullerene derivatives exhibit unique electrical, mechanical, and antimicrobial characteristics that support multifunctional therapeutic applications [29]. Hybrid nanocomposites combine multiple material classes within a single platform to achieve synergistic therapeutic performance. Another important classification of nano-antibiotics is based on stimuli responsiveness. Passive nano-antibiotics generally rely on physicochemical accumulation and diffusion behavior, whereas active or stimuli-responsive systems are engineered to release therapeutic payloads under specific pathological conditions [30]. pH-responsive systems exploit the acidic microenvironment commonly associated with bacterial infection and inflammation. Enzyme-responsive nanoplatforms are activated by bacterial enzymes such as lipases, proteases, or hyaluronidases, enabling selective antimicrobial activation at infected sites. Redox-responsive systems utilize differences in oxidative stress levels between infected and healthy tissues to trigger controlled drug release. External stimuli-responsive systems, including photothermal, magnetic, and ultrasound-responsive nanoplatforms, enable precise therapeutic activation through externally applied energy sources [31]. Such dynamic behavior significantly enhances treatment selectivity and reduces unnecessary exposure of healthy tissues to antimicrobial agents. The classification of nano-antibiotics also increasingly incorporates computational and AI-guided design strategies. Machine learning algorithms are now employed to optimize nanoparticle size, surface charge, hydrophobicity, drug loading efficiency, release kinetics, and toxicity profiles. AI-assisted predictive models can analyze large experimental datasets to identify ideal nanoformulation parameters capable of maximizing antimicrobial activity while minimizing cytotoxicity [32]. This integration of computational intelligence with nanotechnology has accelerated the development of personalized and adaptive antimicrobial systems tailored to specific pathogens and patient conditions. Nano-antibiotics represent a versatile and rapidly evolving therapeutic field that extends beyond conventional antimicrobial paradigms. Their multifunctional nature, tunable physicochemical properties, and capacity for stimuli-responsive precision therapy provide substantial advantages for combating multidrug-resistant infections [13].

**Figure 2:**
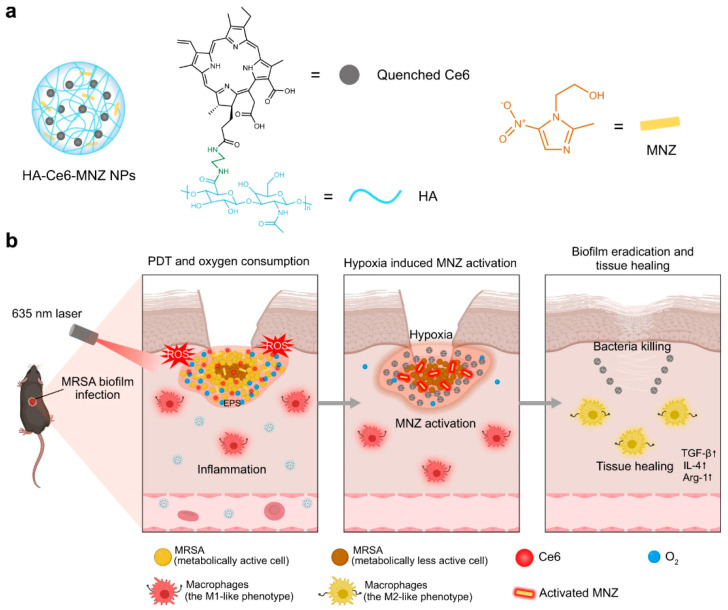
(**a**) Schematic representation of HA-Ce6-MNZ nanoparticles (HCM NPs) composed of hyaluronic acid (HA), chlorin e6 (Ce6), and metronidazole (MNZ), along with their mechanism against MRSA biofilm infections. (**b**) Upon reaching the infected site, the nanoparticles release Ce6 and MNZ, where photodynamic therapy (PDT) enhances local hypoxic conditions to activate MNZ, leading to efficient biofilm disruption, bacterial eradication, and accelerated tissue healing. Adapted from Reference [24] under the Creative Commons CC BY 4.0 license.

As the field continues to evolve through advances in material science, synthetic biology, computational modeling, and artificial intelligence, smart nano-antibiotics are expected to play a transformative role in the future of precision antimicrobial medicine. Figure 2 demonstrates the potential of multifunctional nano-antibiotic systems for overcoming MRSA biofilm-associated infections through hypoxia-enhanced photodynamic therapy. The combined release of Ce6 and metronidazole provides synergistic antibacterial activity, leading to improved biofilm elimination and accelerated infected tissue recovery.

### 2.2. Types of Nanomaterials Used (Metal, Polymeric, Lipid-Based)

Therapeutic performance of smart nano-antibiotics is fundamentally influenced by the physicochemical characteristics of the nanomaterials used in their construction. Different classes of nanomaterials exhibit distinct structural, biological, and functional properties that determine their antimicrobial activity, drug loading capacity, biocompatibility, targeting behavior, and stimuli responsiveness. Among the numerous nanomaterials investigated for antimicrobial applications, metallic nanoparticles, polymeric nanomaterials, and lipid-based nanosystems have emerged as the most extensively studied and clinically promising platforms [33]. These nanomaterials provide unique opportunities for engineering multifunctional antimicrobial systems capable of overcoming microbial resistance, enhancing drug delivery efficiency, and enabling precision therapeutic control. Metal-based nanomaterials possess intrinsic antimicrobial properties and are among the earliest nanostructures explored for infection control applications [34]. Silver nanoparticles are particularly recognized for their broad-spectrum antimicrobial activity against Gram-positive bacteria, Gram-negative bacteria, fungi, and certain viruses. Their antimicrobial mechanism involves membrane disruption, interaction with sulfur-containing proteins, interference with respiratory enzymes, ROS generation, and nucleic acid damage. Due to their strong bactericidal efficiency and relatively low probability of resistance development, silver nanoparticles are widely incorporated into wound dressings, coatings, catheters, and antimicrobial formulations [35]. However, concerns regarding long-term cytotoxicity, ion accumulation, and environmental persistence remain important challenges requiring careful optimization. Gold nanoparticles have also attracted significant attention because of their excellent biocompatibility, tunable optical properties, and ease of surface functionalization. Although gold nanoparticles possess weaker intrinsic antimicrobial activity compared with silver nanoparticles, they are valuable as multifunctional delivery platforms and photothermal therapeutic agents. Surface modification with antibiotics, peptides, antibodies, or nucleic acids enables targeted antimicrobial delivery and enhanced pathogen recognition. Under near-infrared irradiation, gold nanoparticles can generate localized heat capable of disrupting bacterial membranes and biofilms, thereby supporting synergistic antimicrobial therapy [36]. Other metallic nanomaterials including zinc oxide, copper oxide, iron oxide, titanium dioxide, and magnesium oxide nanoparticles have also demonstrated considerable antimicrobial potential. Zinc oxide nanoparticles are particularly attractive because of their photocatalytic ROS generation, UV-blocking properties, and relatively favorable biosafety profile. Copper oxide nanoparticles exhibit potent oxidative stress-mediated antimicrobial effects but may present higher cytotoxicity at elevated concentrations [37]. Magnetic iron oxide nanoparticles provide additional advantages in magnetically guided targeting and imaging applications. Titanium dioxide nanoparticles are widely investigated in photocatalytic antimicrobial systems due to their ability to generate ROS under light activation. Collectively, metal-based nanomaterials offer strong antimicrobial performance; however, their clinical translation requires careful control of dose-dependent toxicity, biodistribution, biodegradability, and long-term biosafety [38]. Polymeric nanomaterials constitute another versatile class of antimicrobial nanoplatforms due to their tunable structure, biodegradability, and exceptional drug encapsulation capability. Polymeric nanoparticles are generally fabricated using natural polymers such as chitosan, alginate, gelatin, dextran, and hyaluronic acid, or synthetic polymers including PLGA, PEG, PCL, and polyacrylamide. These systems can encapsulate hydrophilic and hydrophobic antimicrobial agents while enabling sustained and stimuli-responsive release behavior. Their structural flexibility also supports surface engineering for targeted delivery and immune modulation [39]. Chitosan-based nanoparticles are particularly notable because chitosan itself possesses intrinsic antimicrobial activity arising from electrostatic interaction with negatively charged microbial membranes. Chitosan nanostructures can disrupt membrane permeability, inhibit biofilm formation, and enhance intracellular antibiotic penetration. PLGA nanoparticles have gained extensive biomedical acceptance because of their excellent biodegradability and controlled release properties [40]. PEGylation further improves circulation stability and reduces rapid immune clearance, thereby enhancing systemic therapeutic efficiency. Stimuli-responsive polymeric nanomaterials capable of responding to pH, enzymes, ROS, or temperature changes are increasingly investigated for infection-specific antimicrobial activation. Polymeric micelles, nanogels, dendrimers, and hydrogel-based systems also represent important subclasses of polymeric nano-antibiotics. Dendrimers possess branched architectures with multiple functional groups that facilitate high drug loading and multivalent microbial interaction [41]. Nanogels provide soft, hydrophilic, and absorbent matrices suitable for localized infection therapy and wound healing applications. Injectable hydrogel systems capable of sustained local antibiotic release have demonstrated promising results in chronic wound management and implant-associated infections. Lipid-based nanomaterials are widely recognized for their superior biocompatibility and clinical translational potential [42]. Liposomes represent one of the most established lipid nanocarriers and consist of phospholipid bilayer vesicles capable of encapsulating both hydrophilic and hydrophobic antimicrobial agents. Liposomal encapsulation enhances antibiotic stability, prolongs circulation time, reduces toxicity, and improves intracellular drug delivery [43]. Several liposomal antimicrobial formulations have already received clinical approval, highlighting their therapeutic relevance. Solid lipid nanoparticles and nanostructured lipid carriers provide improved physical stability and controlled drug release compared with conventional liposomes. These systems are particularly advantageous for enhancing poorly soluble antibiotic bioavailability and protecting labile therapeutic agents from degradation [44]. Lipid nanoparticles can also fuse with bacterial membranes, thereby improving antimicrobial penetration and intracellular delivery efficiency. In addition, lipid-based nanosystems demonstrate considerable potential for pulmonary, transdermal, ocular, and oral antimicrobial delivery applications due to their favorable biocompatibility profile. Recent developments increasingly focus on hybrid nanomaterials that combine metallic, polymeric, and lipid components within integrated multifunctional systems [45]. Such hybrid platforms are capable of simultaneously achieving intrinsic antimicrobial activity, targeted drug delivery, stimuli responsiveness, and imaging functionality.

AI-assisted material optimization further accelerates the rational design of nanomaterials with balanced antimicrobial potency, biosafety, and therapeutic precision [46]. Consequently, the strategic selection and engineering of nanomaterials remain central to the successful development of next-generation smart nano-antibiotics for precision antimicrobial therapy.

### 2.3. Mechanisms of Antimicrobial Action

The antimicrobial activity of smart nano-antibiotics arises from a combination of physicochemical interactions, targeted intracellular disruption, and stimuli-responsive therapeutic behavior. Unlike conventional antibiotics that generally depend on a single biochemical target, nano-antibiotics often exhibit multimodal antimicrobial mechanisms that simultaneously attack different structural and metabolic components of microbial cells [47]. This multifunctional behavior significantly reduces the probability of rapid resistance development because microorganisms must overcome multiple independent stress pathways at the same time. In many cases, the nanoscale dimensions of these materials also enhance their interaction with bacterial membranes, allowing improved penetration into biofilms and intracellular infection sites. The greatest strength of nano-antibiotics lies not merely in their antimicrobial potency, but in their ability to destabilize microbial defense systems through coordinated and adaptive mechanisms rather than isolated molecular inhibition. One of the primary antimicrobial mechanisms involves direct disruption of microbial cell membranes. Positively charged nanoparticles can strongly interact with negatively charged bacterial surfaces through electrostatic attraction, leading to membrane destabilization, pore formation, and leakage of intracellular contents [48]. Metallic nanoparticles such as silver and zinc oxide are especially effective in damaging membrane integrity because their nanoscale surfaces can physically penetrate lipid bilayers and alter membrane permeability. This membrane-associated stress frequently results in loss of osmotic balance, ion transport dysfunction, and eventual cell lysis. In Gram-negative bacteria, nanoparticle interaction with lipopolysaccharide-rich outer membranes further enhances structural instability. Polymeric nanomaterials containing cationic functional groups can similarly induce membrane deformation while also facilitating intracellular drug delivery. The membrane-targeted antimicrobial strategies may provide long-term therapeutic advantages because bacterial membranes are less susceptible to rapid mutational adaptation compared with intracellular enzyme targets. Reactive oxygen species (ROS) generation represents another important antimicrobial pathway associated with nano-antibiotics. Several nanomaterials including silver nanoparticles, titanium dioxide, zinc oxide, and copper oxide can catalyze intracellular oxidative stress through formation of superoxide radicals, hydroxyl radicals, and hydrogen peroxide [49]. Excessive ROS production overwhelms microbial antioxidant defense systems and causes oxidation of proteins, lipids, nucleic acids, and membrane structures. The oxidative imbalance generated by nano-antibiotics can be broadly represented as:

O^2^ + e^−^ → O^2^ − ·O^−2^

Subsequent radical conversion pathways further amplify oxidative cellular damage and metabolic collapse. In photocatalytic nanoplatforms, external light stimulation can substantially increase ROS generation efficiency, thereby enabling controlled antimicrobial activation at infection sites [50]. Importantly, ROS-mediated killing mechanisms are nonspecific, which reduces the likelihood of bacteria developing stable resistance pathways. The oxidative stress-based antimicrobial therapy represents one of the most promising approaches for combating multidrug-resistant biofilm-associated infections because it bypasses many traditional resistance mechanisms. Smart nano-antibiotics also interfere with intracellular biomolecular processes after penetrating microbial cells. Certain nanoparticles can bind directly to DNA and RNA, resulting in impaired replication, transcriptional dysfunction, and inhibition of protein synthesis [51]. Metallic ions released from nanoparticles may interact with sulfur-containing enzymes and ribosomal proteins, causing irreversible metabolic disturbances. In addition, nanocarriers loaded with antibiotics can improve intracellular drug concentration by overcoming efflux pump-mediated resistance and membrane permeability barriers. This enhanced intracellular accumulation is particularly important in chronic infections caused by intracellular pathogens or biofilm-protected bacterial populations. Stimuli-responsive nanoplatforms further improve therapeutic specificity by releasing antimicrobial agents selectively under infection-associated conditions such as acidic pH, enzyme secretion, or elevated ROS concentration. The controlled intracellular delivery one of the most transformative aspects of smart nano-antibiotics because it shifts antimicrobial therapy from passive diffusion toward active therapeutic targeting. Another critical mechanism involves disruption of microbial biofilms, which are resistant multicellular communities embedded within extracellular polymeric matrices [30]. Biofilms act as physical and biochemical barriers that severely reduce antibiotic penetration and protect bacteria from immune clearance. Due to their nanoscale dimensions and surface tunability, nano-antibiotics can penetrate biofilm structures more effectively than conventional antibiotics. Some nanomaterials also degrade extracellular polymeric substances or interfere with quorum sensing pathways responsible for microbial communication and biofilm maturation [51]. Photothermal and magnetically responsive nanoplatforms can additionally induce localized thermal or mechanical disruption of biofilm architecture, thereby increasing bacterial susceptibility to antimicrobial agents. The antibiofilm capability of smart nanoplatforms may ultimately become one of the decisive factors determining their future clinical success, particularly in implant-associated and chronic wound infections. Table 2 summarizes the diverse and multifunctional antimicrobial mechanisms employed by smart nano-antibiotic systems to combat resistant microbial pathogens. Unlike conventional antibiotics that mainly rely on single biochemical targets, nanotechnology-based antimicrobial platforms utilize simultaneous physical, chemical, and biological interactions to enhance bacterial eradication efficiency. The table further demonstrates how advanced nanomaterials can overcome major antimicrobial resistance barriers including biofilm protection, intracellular persistence, oxidative defense mechanisms, and microbial communication pathways. Integration of controlled drug release, targeted delivery, and synergistic therapeutic actions enables these nanosystems to achieve higher antimicrobial precision with reduced resistance development. The mechanisms presented in this table highlight the potential of smart nano-antibiotics as next-generation multifunctional therapeutic platforms for managing multidrug-resistant infections.

### 2.4. Advantages over Traditional Antibiotics

Smart nano-antibiotics offer several important advantages over conventional antimicrobial therapies due to their multifunctional therapeutic behavior, adaptive responsiveness, and engineered nanoscale properties [60]. Traditional antibiotics generally rely on single-target biochemical inhibition, whereas smart nanoplatforms integrate multiple antimicrobial pathways within a unified system. This distinction becomes particularly significant in the treatment of multidrug-resistant infections where microorganisms have already evolved defense mechanisms against classical therapeutic agents. Nano-antibiotics not only improve antimicrobial efficacy but also enhance drug delivery precision, reduce systemic toxicity, and enable programmable therapeutic responses under pathological conditions. The transition from conventional antibiotics to smart nano-antibiotics represents a broader evolution from static pharmacology toward dynamic and biologically interactive antimicrobial therapy. One of the major advantages of nano-antibiotics is their ability to overcome microbial resistance mechanisms. Conventional antibiotics are vulnerable to bacterial enzymatic degradation, target-site mutation, reduced membrane permeability, and active efflux pump systems [22]. In contrast, nano-antibiotics frequently exhibit simultaneous membrane disruption, oxidative stress induction, intracellular damage, and controlled drug release mechanisms that collectively reduce bacterial adaptability. Multifunctional nanoplatforms also improve antimicrobial penetration into biofilms and intracellular compartments where traditional antibiotics often fail to reach therapeutic concentrations [61]. Because many nanomaterials act through physicochemical interactions rather than specific molecular binding, microorganisms face greater difficulty in developing stable resistance pathways. This reduced susceptibility to resistance development could make nano-antibiotics an essential component of future antimicrobial strategies against persistent and evolving pathogens. Targeted and stimuli-responsive drug delivery is another major advantage of smart nano-antibiotics. Conventional antibiotics distribute systemically throughout the body, frequently exposing healthy tissues to unnecessary drug concentrations and increasing the risk of adverse side effects. Smart nanoplatforms can selectively accumulate at infection sites through passive targeting, ligand-mediated recognition, or stimuli-triggered activation. Infection-associated environmental changes such as acidic pH, elevated enzyme activity, hypoxia, and oxidative stress can trigger controlled release of antimicrobial agents precisely where therapy is needed [62]. External stimuli including light, magnetic fields, and ultrasound can further improve spatial and temporal control of antimicrobial activation. Such selective delivery minimizes off-target toxicity while improving therapeutic concentration at infected tissues. The precision-controlled drug release may ultimately redefine antimicrobial pharmacokinetics by allowing therapies to adapt dynamically to local disease microenvironments rather than relying solely on systemic administration. Nano-antibiotics also provide substantial improvements in drug stability, solubility, and pharmacokinetic behavior. Many conventional antibiotics suffer from poor aqueous solubility, rapid degradation, limited circulation time, and insufficient tissue penetration [63]. Nanocarrier systems can encapsulate unstable or poorly soluble antibiotics within protective matrices that improve bioavailability and prolong therapeutic activity. Controlled release kinetics may be mathematically represented as:

dMt/dt = k (M∞ − Mt)

where Mt represents released drug concentration at time *t*, and *k* denotes the release constant. Such controlled release profiles enable sustained antimicrobial exposure while reducing dosing frequency and peak-dose toxicity. Lipid-based and polymeric nanocarriers additionally improve penetration across biological barriers and facilitate intracellular drug accumulation. The pharmacokinetic optimization one of the most clinically relevant advantages of smart nanotechnology because therapeutic failure often arises not from lack of drug potency, but from inadequate delivery efficiency. Another important advantage involves multifunctional therapeutic integration. Smart nano-antibiotics can combine antimicrobial therapy with imaging, biosensing, photothermal treatment, immune modulation, and real-time monitoring capabilities within a single nanoplatform [64]. Such theranostic systems enable simultaneous diagnosis and treatment while allowing clinicians to evaluate therapeutic response dynamically. AI-assisted nanoformulation optimization further enhances treatment personalization by predicting optimal nanoparticle design parameters based on infection type, microbial profile, and patient-specific biological conditions [65]. This convergence of nanotechnology, precision medicine, and computational intelligence creates opportunities for adaptive antimicrobial therapies that were previously impossible using conventional antibiotics alone. Future clinical importance of smart nano-antibiotics will depend not only on their antimicrobial performance, but also on their ability to function as intelligent therapeutic systems capable of integrating diagnosis, targeting, monitoring, and controlled intervention within a unified platform.

## 3. Stimuli-Responsive Nanoplatforms for Drug Delivery

Stimuli-responsive nanoplatforms represent a key advancement in antimicrobial nanomedicine by enabling controlled drug release in response to specific biological or external signals. Unlike conventional nanocarriers that release therapeutic agents passively, these systems are engineered to undergo structural or physicochemical changes when exposed to predefined triggers such as pH variation, enzymatic activity, temperature fluctuation, oxidative stress, light irradiation, magnetic fields, or ultrasound. The primary objective of stimuli-responsive delivery is to regulate the timing, rate, and location of antimicrobial release according to local microenvironmental conditions. Based on the triggering mechanism, these systems can be classified into pH-responsive, enzyme-responsive, temperature-responsive, redox-responsive, and multi-stimuli responsive nanoplatforms. Understanding the mechanisms governing stimulus recognition and drug release is essential for designing advanced nano-antibiotic systems with improved therapeutic control.

### 3.1. pH-Responsive Systems

pH-responsive nanoplatforms have attracted considerable attention because many infection-associated microenvironments exhibit acidic conditions that differ from those of healthy tissues [66]. This characteristic provides an opportunity to engineer nanocarriers that undergo controlled physicochemical changes in response to local pH variations, thereby regulating antimicrobial release. The effectiveness of these systems primarily depends on the selection of suitable pH-sensitive materials and their ability to respond predictably within biologically relevant pH ranges. The fundamental principle of pH-responsive systems involves the incorporation of ionizable functional groups or acid-labile chemical bonds that undergo structural transformation under acidic conditions [67]. Common pH-sensitive materials include poly(histidine), poly(β-amino esters), chitosan, poly(acrylic acid), alginate, hydrazone linkages, and Schiff-base bonds. Under physiological pH, these materials generally maintain structural stability and retain the encapsulated drug. However, exposure to acidic environments induces protonation, bond cleavage, polymer swelling, or carrier destabilization, resulting in controlled release of the antimicrobial payload. The protonation process can be represented as:

*R* − *NH*_2_ + *H*^+^ → *R* − *NH*_3_^+^

**Figure 3:**
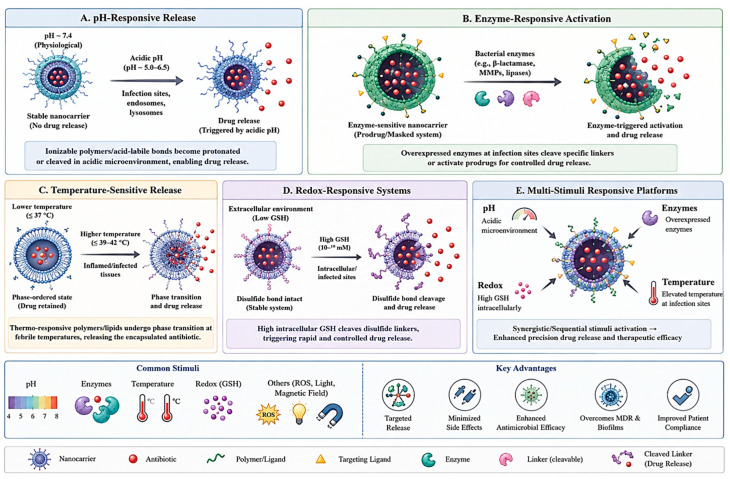
Schematic presentation of different stimuli-responsive nano-antibiotic drug delivery systems. The figure summarizes representative smart nanocarriers designed to respond to endogenous and exogenous stimuli, including acidic pH, bacterial enzymes, temperature variations, oxidative stress, and combined multi-stimuli conditions. Trigger-induced changes in nanoparticle structure, surface properties, or carrier stability enable site-specific activation and controlled antimicrobial release. These responsive mechanisms provide a versatile platform for enhancing the effectiveness of advanced antimicrobial nanomedicine.

Such pH-induced transformations alter carrier architecture and facilitate the release of therapeutic agents in response to environmental changes [68]. Different pH-responsive nanocarriers employ distinct release mechanisms depending on their composition and structural design. Polymeric nanoparticles often rely on protonation-induced swelling or degradation, whereas liposomal systems may undergo membrane destabilization under acidic conditions. Similarly, mesoporous silica nanoparticles frequently utilize acid-cleavable gatekeepers that detach in response to reduced pH, allowing drug diffusion from internal pores. The versatility of these mechanisms enables the development of nanoplatforms with tunable release profiles and adjustable activation thresholds suitable for different infection-associated environments [69]. Recent advances have focused on improving the responsiveness, stability, and release kinetics of pH-sensitive nanomaterials. Factors such as polymer composition, molecular weight, crosslinking density, surface charge, and pKa values strongly influence carrier performance and drug-release behavior [70]. Precise control of these parameters is essential for achieving predictable activation and minimizing unintended drug leakage during circulation. In addition, the integration of computational modeling and AI-assisted formulation design may facilitate the optimization of pH-responsive systems by predicting material behavior under varying physiological conditions. Despite significant progress, several challenges remain. Physiological pH variations among tissues, differences in infection microenvironments, and manufacturing-related variability may affect release efficiency and reproducibility [71]. Furthermore, maintaining carrier stability under physiological conditions while ensuring rapid activation under acidic conditions remains a key design consideration. Future research should focus on developing robust pH-sensitive materials with well-defined response characteristics, scalable fabrication methods, and improved control over release kinetics to support clinical translation. Figure 3 illustrates different stimuli-responsive nano-antibiotic systems developed for precision antimicrobial therapy. The schematic highlights how smart nanocarriers respond to infection-associated conditions such as acidic pH, bacterial enzymes, temperature changes, oxidative imbalance, and multiple biological triggers for controlled drug release. These adaptive systems improve infection-site targeting, enhance biofilm penetration, and reduce unwanted systemic toxicity. Stimuli-responsive nanoplatforms offer a more efficient and selective antimicrobial strategy compared with conventional antibiotic delivery systems.

### 3.2. Enzyme-Responsive Systems

Enzyme-responsive nanoplatforms represent an important class of smart antimicrobial delivery systems that utilize infection-associated enzymatic activity as endogenous triggers for controlled drug release. During bacterial infections, microorganisms and host inflammatory responses produce a variety of enzymes, including proteases, lipases, phospholipases, hyaluronidases, gelatinases, and β-lactamases, which create distinct biochemical environments within infected tissues [72]. These enzymes provide specific molecular signals that can be exploited to activate nanocarriers selectively under pathological conditions. As a result, enzyme-responsive systems have attracted considerable interest for their ability to regulate antimicrobial release according to local biological cues. The design of enzyme-responsive nanocarriers typically involves the incorporation of enzyme-cleavable substrates, peptide sequences, degradable polymers, or chemically sensitive linkers within the nanoparticle structure [73]. Under normal physiological conditions, these materials remain stable and retain their therapeutic cargo. However, when exposed to target enzymes, specific cleavage reactions induce structural changes that destabilize the carrier and initiate drug release. A generalized enzymatic reaction can be expressed as:

*Substrate* + *Enzyme* → *Products*

Depending on the nanoplatform design, enzymatic cleavage may trigger polymer degradation, removal of protective coatings, opening of nanoporous gates, or disassembly of carrier matrices. The responsiveness and release kinetics are largely governed by enzyme concentration, substrate specificity, and linker chemistry. Various material systems have been developed for enzyme-responsive antimicrobial delivery. Peptide-based linkers are widely employed because their amino acid sequences can be tailored to specific enzymes. Similarly, lipid-based nanocarriers may incorporate lipase-sensitive components that undergo degradation in enzyme-rich environments. Polymeric nanoparticles containing degradable ester, amide, or peptide bonds have also demonstrated effective enzyme-triggered release behavior. In addition, hybrid nanostructures combining multiple responsive components offer greater flexibility in controlling activation profiles and release rates [74]. The selection of appropriate responsive materials is therefore essential for achieving predictable and efficient drug-release performance. Recent advances have focused on improving the specificity and responsiveness of enzyme-sensitive nanomaterials through rational molecular design. Factors such as substrate accessibility, cleavage efficiency, linker stability, carrier architecture, and enzyme-substrate affinity strongly influence therapeutic performance [75]. Computational modeling and AI-assisted material screening may further accelerate the identification of optimal enzyme-responsive components and release parameters. These approaches can facilitate the development of nanocarriers with tunable activation thresholds and improved reproducibility. Despite significant progress, several challenges remain. Enzyme expression levels may vary considerably among pathogens, infection stages, and physiological environments, potentially affecting activation efficiency [76]. In addition, maintaining carrier stability during circulation while preserving rapid enzyme responsiveness remains an important design consideration. Future research should focus on developing robust enzyme-sensitive materials, improving control over release kinetics, and establishing scalable manufacturing strategies to support the clinical translation of enzyme-responsive nano-antibiotic systems [77,78].

### 3.3. Temperature-Responsive Systems

Temperature-responsive nanoplatforms represent an important class of smart antimicrobial delivery systems that utilize thermal stimuli to regulate drug release. Localized infections and inflammatory environments often exhibit elevated temperatures resulting from increased metabolic activity, vascular changes, and immune responses [79]. In addition, external heat sources such as near-infrared irradiation, magnetic fields, ultrasound, and microwave stimulation can generate controlled thermal conditions for activating nanocarriers. These systems are designed to undergo physicochemical changes at predefined temperature thresholds, enabling temperature-dependent release of antimicrobial agents. The functionality of temperature-responsive nanocarriers is primarily based on thermosensitive materials that exhibit lower critical solution temperature (LCST) or upper critical solution temperature (UCST) behavior [80]. Below the transition temperature, these materials generally remain hydrated and structurally stable, whereas exceeding the transition threshold induces dehydration, polymer collapse, phase transition, or matrix destabilization. These changes facilitate the release of encapsulated therapeutic agents. Thermodynamic behavior governing these transitions can be described as:

Δ*G* = Δ*H* − *T*Δ*S*

where temperature-dependent free energy changes influence the structural state of Thermoresponsive material. Commonly investigated materials include poly(N-isopropylacrylamide) (PNIPAM), polyethylene glycol-based copolymers, thermosensitive hydrogels, and temperature-responsive polymeric micelles. The selection of appropriate transition temperatures is critical for achieving reliable and controllable drug-release behavior. Various temperature-responsive nanocarriers employ distinct release mechanisms depending on their composition and architecture. Polymeric nanoparticles often undergo volume changes or conformational transitions, whereas thermosensitive hydrogels may experience sol–gel transformations that alter drug diffusion rates. Liposomal systems can exhibit membrane permeability changes under elevated temperatures, promoting release of encapsulated agents. In addition, hybrid nanomaterials incorporating photothermal or magnetothermal components can generate localized heat upon external stimulation, providing an additional level of control over activation and release kinetics [55]. These approaches enable the development of nanoplatforms with tunable thermal responsiveness and adjustable release profiles. Recent research has focused on improving the responsiveness, stability, and reproducibility of thermoresponsive materials through molecular engineering and material optimization [81]. Factors including polymer composition, molecular weight, crosslinking density, particle size, and transition temperature significantly influence carrier performance. Computational modeling and AI-assisted material screening may further facilitate the rational design of temperature-sensitive nanocarriers with predictable activation behavior under diverse physiological conditions. Despite considerable progress, several challenges remain. Precise control of thermal thresholds is essential to ensure consistent activation while maintaining carrier stability during circulation [82]. Variations in local temperature distribution and material properties may influence release efficiency and reproducibility. Also, scalable fabrication and long-term storage stability remain important considerations for future development. Continued advances in thermoresponsive materials, manufacturing strategies, and release-control technologies are expected to support the clinical translation of temperature-responsive nano-antibiotic systems [83].

### 3.4. Redox-Responsive Systems

Redox-responsive nanoplatforms have emerged as an important class of smart antimicrobial delivery systems because infection-associated microenvironments frequently exhibit altered oxidative and reductive conditions compared with healthy tissues [84]. During bacterial infections, immune activation and inflammatory processes generate elevated levels of reactive oxygen species (ROS) and other redox-active molecules, creating biochemical environments that can serve as endogenous triggers for drug release. By exploiting these changes, redox-responsive nanocarriers enable controlled activation through selective chemical transformations within the carrier structure. The design of redox-responsive systems primarily relies on incorporating oxidation-sensitive or reduction-sensitive chemical groups into nanoparticle architectures [85]. Common responsive moieties include disulfide bonds, thioketal linkages, selenium-containing compounds, and ROS-sensitive polymers. Under reducing or oxidative conditions, these groups undergo cleavage or degradation, resulting in destabilization of the carrier and subsequent release of the encapsulated therapeutic payload. A representative reduction-sensitive reaction involving disulfide bond cleavage can be expressed as:

*R* − *S* − *S* − *R* + 2*H*^+^
*+* 2*e*^−^ → 2*R* − *SH*

This bond cleavage alters the structural integrity of the nanocarrier and facilitates controlled drug release in response to local redox conditions. Similarly, ROS-responsive polymers may degrade directly upon exposure to oxidizing species such as hydrogen peroxide, resulting in activation of the delivery system. A variety of redox-responsive materials have been investigated for antimicrobial applications. Polymeric nanoparticles containing disulfide-crosslinked networks are among the most commonly studied systems because of their well-defined degradation behavior. Thioketal-based polymers have also attracted considerable interest owing to their sensitivity toward ROS-rich environments. In addition, hybrid nanostructures integrating redox-responsive polymers with lipid, metallic, or mesoporous components offer enhanced flexibility in controlling release kinetics and carrier stability [86]. The selection of responsive materials and linker chemistry plays a critical role in determining activation efficiency, degradation rate, and therapeutic performance. Recent advances have focused on improving the responsiveness and reliability of redox-sensitive nanomaterials through molecular engineering and computational design approaches [87]. Parameters such as bond stability, polymer composition, crosslinking density, nanoparticle size, and degradation kinetics strongly influence drug-release behavior. AI-assisted material screening and computational modeling may further support the identification of optimal redox-responsive architectures with predictable activation characteristics under diverse physiological conditions. Despite their significant potential, several challenges remain. Redox conditions can vary substantially among different infection environments, which may affect activation efficiency and release reproducibility [88]. In addition, maintaining carrier stability during circulation while preserving sufficient responsiveness at the target site remains an important design consideration. Future research should focus on developing robust redox-sensitive materials, improving control over degradation behavior, and establishing scalable fabrication strategies to support the clinical translation of redox-responsive nano-antibiotic systems.

### 3.5. Multi-Stimuli Responsive Nanocarriers

Multi-stimuli responsive nanocarriers represent one of the most advanced developments in smart antimicrobial nanotechnology because they integrate responsiveness to two or more biological or external triggers within a single therapeutic platform. Unlike single-stimulus systems that rely on only one environmental signal, these nanocarriers are designed to respond to multiple pathological conditions simultaneously or sequentially, thereby providing greater control over drug release behavior [89]. Since infectious microenvironments often exhibit overlapping characteristics such as acidic pH, elevated enzymatic activity, oxidative stress, temperature variation, and hypoxia, multi-responsive systems offer a versatile strategy for regulating therapeutic activation under complex biological conditions. The construction of multi-stimuli responsive nanoplatforms generally involves combining different responsive polymers, chemical linkers, surface coatings, or functional layers within a unified nanoscale architecture [90]. For example, a nanoparticle may incorporate pH-sensitive polymers together with ROS-cleavable bonds and enzyme-degradable components. Under specific environmental conditions, each responsive element undergoes structural changes that collectively contribute to controlled release of Therapeutic payload. Such designs allow hierarchical activation processes in which different stimuli trigger distinct stages of carrier destabilization and drug release. The response of these systems can be conceptually represented as:

*R_total_* = *R_pH_* + *R_ROS_*

where the total release behavior depends on the combined contribution of multiple stimuli-responsive mechanisms [91]. The relative contribution of each trigger is influenced by material composition, environmental conditions, and carrier architecture. Consequently, careful selection of responsive components is essential for achieving predictable activation and controlled release profiles. A variety of material combinations have been explored for developing multi-responsive nanocarriers. Similar multi-responsive design principles have been reported in advanced nanoplatforms that integrate pH, ROS, and external stimuli to achieve sequential therapeutic activation under complex pathological microenvironments [92]. Polymeric nanoparticles, liposomes, hydrogels, mesoporous silica nanoparticles, and hybrid nanocomposites have all been engineered with multiple responsive functionalities. In some systems, endogenous triggers such as pH, enzymes, and redox conditions are combined, whereas others integrate internal stimuli with externally applied factors including light, magnetic fields, ultrasound, or thermal activation [93]. These approaches provide greater flexibility in regulating release kinetics and therapeutic activation. Recent advances have focused on improving the structural stability, responsiveness, and reproducibility of multi-responsive nanoplatforms. The use of modular carrier designs, biodegradable polymers, standardized linker chemistries, and quality-by-design manufacturing approaches has shown promise in improving formulation consistency. In addition, computational modeling and AI-assisted material optimization may facilitate the identification of optimal material combinations, trigger thresholds, and release parameters that support reliable multifunctional performance under physiologically relevant conditions. Despite their considerable potential, multi-stimuli responsive systems remain scientifically complex. The incorporation of multiple responsive elements can increase formulation complexity, introduce interactions between different activation pathways, and complicate manufacturing processes [94]. Achieving reproducible release behavior while maintaining carrier stability during storage and circulation remains a major challenge. Future research should focus on simplifying carrier architectures, improving manufacturing scalability, and developing robust design strategies capable of delivering predictable multifunctional responsiveness under clinically relevant conditions. Table 3 represented the major categories of stimuli-responsive nano-antibiotic systems designed for precision antimicrobial therapy. The table highlights how different pathological or external triggers can selectively activate controlled drug release and improve infection-site targeting. These smart nanoplatforms enhance therapeutic efficacy, minimize systemic toxicity, and provide adaptive antimicrobial responses against complex multi-drug-resistant infections.

## 4. Artificial Intelligence in Nano-Antibiotic Design

While stimuli-responsive nanoplatforms provide the foundation for controlled antimicrobial delivery, their successful design and optimization increasingly depend on advanced computational approaches. Artificial intelligence has emerged as a powerful tool for accelerating nanomaterial development, predicting biological interactions, and improving therapeutic performance. Therefore, the integration of AI with smart nanotechnology represents a critical step toward precision antimicrobial therapy.

### 4.1. Machine Learning Models for Nanomaterial Design

**Figure 4:**
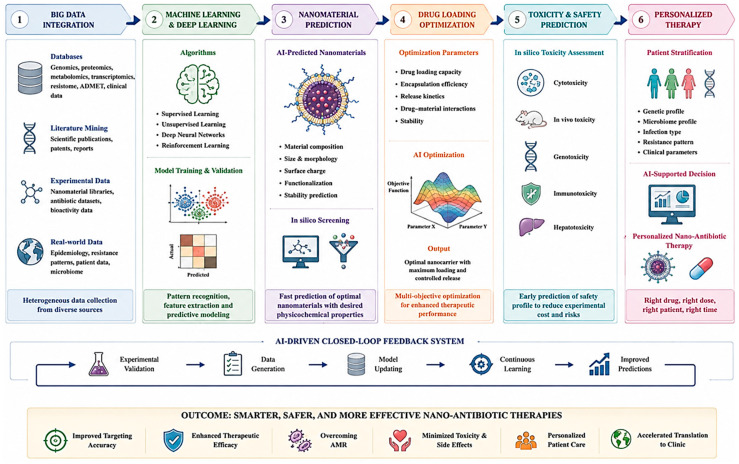
Artificial Intelligence Workflow in Smart Nano-Antibiotic Design and Optimization.

Machine learning (ML) has emerged as an important computational tool for the rational design of smart nano-antibiotic systems by establishing quantitative relationships between nanomaterial characteristics and biological performance [95,96]. Unlike conventional trial-and-error approaches, ML models can rapidly analyze large experimental datasets and identify patterns that guide nanomaterial optimization. Several ML algorithms have been applied in antimicrobial nanomedicine, including random forest (RF), support vector machine (SVM), decision tree (DT), gradient boosting (GB), artificial neural networks (ANN), and deep learning models [97]. These algorithms are capable of handling complex nonlinear relationships among multiple formulation variables and can generate predictive models with high accuracy. The predictive performance of ML models depends on the quality of input parameters used during training. Common inputs include nanoparticle size, polydispersity index, surface charge (zeta potential), morphology, chemical composition, crystallinity, ligand density, hydrophobicity, polymer composition, drug-loading capacity, encapsulation efficiency, and release kinetics. Additional biological descriptors such as bacterial strain type, minimum inhibitory concentration (MIC), cytotoxicity data, and cellular uptake behavior may also be incorporated into model development [98,99]. Based on these inputs, ML algorithms can predict a wide range of outputs relevant to nano-antibiotic development. Typical predicted outcomes include antimicrobial activity, bacterial inhibition efficiency, nanoparticle stability, drug release behavior, cellular internalization, biodistribution, toxicity profiles, and formulation performance. The predictive relationship can be represented as:

*y* = *f* (*x*_1_, *x*_2_, *x*_3_, …, *x_n_*)

where x*_n_* represents nanomaterial descriptors and y represents the predicted biological or therapeutic response. Recent studies have demonstrated the utility of ML models in screening large numbers of nanoparticle formulations and identifying candidates with improved antimicrobial efficacy and safety profiles prior to experimental validation. Such computational approaches reduce development time, lower experimental costs, and improve formulation selection efficiency. Despite these advantages, broader implementation remains dependent on the availability of standardized, high-quality datasets and interpretable AI frameworks capable of providing mechanistic insights into nano-bio interactions [46]. Machine learning models are increasingly being applied to predict key nanomaterial properties relevant to antimicrobial performance. Common input parameters include nanoparticle size, surface charge, morphology, drug loading efficiency, polymer composition, hydrophobicity, and release kinetics. Algorithms such as random forest, support vector machine, gradient boosting, and artificial neural networks have been employed to establish quantitative relationships between material characteristics and biological outcomes. These predictive models can accelerate formulation screening and reduce experimental workload by identifying promising nano-antibiotic candidates prior to laboratory validation. Figure 4 presents the role of artificial intelligence in the development of smart nano-antibiotic systems. The schematic shows how AI and machine learning can analyze biological and nanomaterial data to optimize drug loading, release behavior, antimicrobial activity, and safety profiles. Integration of computational modeling with nanotechnology supports faster and more precise development of advanced antimicrobial nanoplatforms for combating resistant infections.

### 4.2. AI in Drug Loading and Release Optimization

Artificial intelligence has become an important tool for optimizing drug loading and release behavior in smart nano-antibiotic systems by analyzing complex relationships between formulation variables and therapeutic performance [100]. Traditional optimization approaches often require extensive experimental screening of material compositions, carrier structures, and preparation conditions. AI-based methods can accelerate this process by identifying optimal formulation parameters from large experimental datasets. Among AI approaches, deep learning models have shown particular promise for handling high-dimensional nanomedicine datasets. Convolutional neural networks (CNNs) are widely used for image-based analysis of nanomaterials, including nanoparticle morphology classification, particle size estimation, surface characterization, and structural quality assessment from electron microscopy and imaging datasets [101]. Automated image analysis using CNNs reduces manual interpretation and enables rapid evaluation of formulation consistency. Graph neural networks (GNNs) have emerged as powerful tools for predicting nanomaterial properties because they can effectively represent complex structural relationships between nanoparticle components, surface ligands, polymers, and therapeutic payloads [102]. By learning interactions within these networks, GNN models can predict nanoparticle stability, drug-loading capacity, release behavior, and biological performance with improved accuracy. Deep learning algorithms are also increasingly applied for nanoparticle-property prediction using physicochemical descriptors such as particle size, surface charge, composition, porosity, hydrophobicity, encapsulation efficiency, and degradation characteristics [103]. These models can establish quantitative relationships between formulation parameters and expected outcomes, including drug release profiles, antimicrobial activity, cellular uptake, and biosafety characteristics. Recent studies have demonstrated that AI-assisted prediction models can significantly reduce experimental workload by identifying promising nanoformulations prior to laboratory testing [104,105]. Nevertheless, the reliability of these approaches remains dependent on the availability of high-quality datasets, standardized characterization methods, and robust experimental validation. Continued advances in deep learning and nanomaterial informatics are expected to further improve the accuracy and efficiency of smart nano-antibiotic design. In details deep learning approaches enable the analysis of complex and high-dimensional datasets generated from nanomaterial characterization, imaging studies, omics platforms, and biological assays. Convolutional neural networks have been utilized for nanoparticle image analysis and morphology classification, whereas recurrent and graph neural networks have shown potential for predicting nanomaterial biological interactions and antimicrobial efficacy. These advanced models provide improved prediction accuracy compared with conventional statistical methods, particularly when large datasets are available.

### 4.3. Predictive Modeling of Nano Bio Interactions

Predictive modeling has become an important application of artificial intelligence in smart nano-antibiotic development by enabling the estimation of critical formulation parameters that influence biological performance [106]. The behavior of nanoparticles is strongly affected by multiple physicochemical characteristics, making experimental optimization both time-consuming and resource intensive. AI-based predictive models provide a computational framework for identifying relationships between formulation variables and therapeutic outcomes. Machine learning algorithms are increasingly used to predict nanoparticle size, polydispersity index (PDI), zeta potential, drug-loading capacity, and release behavior based on synthesis conditions and material composition [107]. These parameters are among the most important descriptors governing nanoparticle stability, dispersion, cellular interaction, and therapeutic performance. By analyzing large experimental datasets, AI models can identify optimal formulation conditions while reducing the need for extensive trial-and-error experimentation. Particle size prediction is particularly important because size influences circulation behavior, cellular uptake, and antimicrobial activity. Similarly, PDI serves as an indicator of particle uniformity and formulation quality, while zeta potential provides information regarding colloidal stability and surface interactions. AI algorithms can establish quantitative relationships between formulation variables and these physicochemical properties, enabling more efficient nanocarrier optimization. Predictive modeling is also widely applied to estimate drug-loading efficiency and release profiles. Input variables such as polymer composition, lipid ratio, surface chemistry, porosity, and preparation conditions can be correlated with encapsulation efficiency and release kinetics. These models assist researchers in selecting formulations capable of achieving desired therapeutic concentrations and controlled drug release characteristics. Recent studies have demonstrated that machine learning models can accurately predict multiple formulation parameters simultaneously, including particle size, PDI, zeta potential, drug-loading efficiency, and release behavior [108]. Such computational approaches improve formulation screening efficiency, reduce development costs, and accelerate the design of smart nano-antibiotic systems. Nevertheless, reliable prediction remains dependent on high-quality datasets, standardized characterization methods, and rigorous experimental validation to ensure model accuracy and reproducibility [109]. Despite significant advances in predictive modeling, improving the interpretability of AI-generated predictions remains a major challenge in nano–bio interaction research. Future AI systems may benefit from the integration of explainable artificial intelligence (XAI), which can identify the specific physicochemical properties and biological factors that most strongly influence nanoparticle behavior. In addition, physics-informed machine learning and mechanistic modeling approaches can incorporate established biological principles into predictive frameworks, enabling more transparent and biologically meaningful predictions. Combining computational models with experimental validation, high-resolution imaging, and multi-omics datasets may further reveal the molecular mechanisms governing nanoparticle uptake, biodistribution, protein corona formation, immune responses, and therapeutic outcomes. Such developments are expected to enhance both the reliability and scientific interpretability of AI-guided nano-antibiotic design.

### 4.4. AI in Toxicity and Safety Assessment

Recent studies have employed machine learning datasets ranging from several hundred to thousands of nanoparticle formulations generated from published literature, experimental databases, and high-throughput screening platforms. Common validation strategies include independent training-test datasets, k-fold cross-validation, external validation datasets, and performance metrics such as accuracy, mean squared error, receiver operating characteristic curves, and area under the curve values. Typical predictive endpoints include nanoparticle size, polydispersity index, zeta potential, drug-loading efficiency, release kinetics, antimicrobial activity, and toxicity profiles. However, differences in experimental protocols, limited dataset availability, and lack of standardized reporting remain major challenges affecting model reliability and generalizability. Toxicity and biosafety evaluation remain among the most significant challenges limiting the clinical translation of smart nano-antibiotics. Although nanomaterials offer remarkable therapeutic advantages, their small size, high surface reactivity, and complex biological interactions may also induce unintended cytotoxicity, oxidative stress, inflammation, immune dysfunction, and long-term organ accumulation [110]. Conventional toxicological assessment methods typically require extensive in vitro and in vivo experimentation, which is time-consuming, costly, and often unable to fully capture the multidimensional behavior of nanomaterials within biological systems. Artificial intelligence has therefore emerged as a transformative tool for accelerating toxicity prediction and improving safety-oriented nanomaterial design. The AI-assisted toxicity modeling may become equally as important as antimicrobial optimization because clinical success ultimately depends on achieving an appropriate balance between therapeutic efficacy and biological safety. AI-based toxicological models analyze relationships between nanoparticle physicochemical properties and observed biological responses using large experimental datasets [111]. Important predictive parameters include particle size, shape, surface area, zeta potential, aggregation behavior, solubility, crystallinity, and chemical composition. Machine learning algorithms such as random forests, support vector machines, neural networks, and quantitative structure-activity relationship (QSAR) models are widely employed for predicting cytotoxicity, oxidative stress generation, inflammatory signaling, hemocompatibility, and genotoxicity. A simplified toxicity prediction function may be represented as:

*Toxicity* = *f* (*Size*, *Charge*, *Surface Area*, *Composition*)

Such computational approaches allow researchers to identify potentially hazardous nanomaterial characteristics before extensive biological testing. The early-stage predictive screening can substantially reduce developmental cost while simultaneously improving patient safety. One of the most important applications of AI in safety assessment involves prediction of oxidative stress-mediated toxicity. Many antimicrobial nanomaterials intentionally generate reactive oxygen species to destroy microbial cells; however, excessive ROS generation may also damage healthy tissues. AI systems can evaluate oxidative potential by analyzing nanoparticle composition, catalytic activity, surface defects, and environmental conditions. Predictive models are increasingly capable of estimating the probability of mitochondrial dysfunction, lipid peroxidation, DNA damage, and inflammatory cytokine activation under different biological scenarios [112]. Such computational insight is particularly important for metallic nanoparticles including silver, copper oxide, and titanium dioxide systems that exhibit strong oxidative reactivity. The optimizing antimicrobial ROS production while minimizing host oxidative injury represents one of the central design challenges in future nano-antibiotic development. AI-driven toxicity assessment is also valuable for predicting nanoparticle biodistribution and long-term accumulation. Certain nanomaterials may persist within organs such as the liver, spleen, lungs, kidneys, or brain, potentially causing chronic toxicity and inflammatory complications. Machine learning models trained on pharmacokinetic and imaging datasets can estimate nanoparticle circulation time, tissue deposition, metabolic transformation, and clearance pathways. Such predictive systems help researchers modify nanoparticle size, surface chemistry, and degradability to improve biosafety profiles [97]. AI-assisted simulation of immune interactions additionally enables prediction of complement activation, macrophage uptake, and immunogenicity. The future antimicrobial nanoplatforms must prioritize biodegradability and controlled elimination as fundamental design principles rather than secondary considerations. Another emerging application involves AI-guided personalized safety assessment. Biological responses to nanoparticles vary considerably depending on patient-specific factors including genetics, immune status, microbiome composition, metabolic profile, and disease condition 226. Integrating clinical and biological datasets with machine learning algorithms may eventually allow prediction of individualized toxicity risk before therapeutic administration. Such precision safety modeling could significantly improve treatment reliability in vulnerable patient populations. Furthermore, AI-assisted robotic screening platforms are increasingly capable of automating high-throughput nanotoxicology analysis, thereby accelerating regulatory evaluation and formulation optimization [113]. The integration of AI with personalized medicine may fundamentally reshape future nanomedicine safety evaluation by shifting from generalized toxicity assumptions toward individualized risk prediction. Despite substantial progress, important limitations still remain. AI models are dependent on the availability of standardized, high-quality experimental datasets, yet nanotoxicology data often vary significantly across studies due to differences in synthesis methods, assay conditions, and biological models [111]. Many predictive systems also lack mechanistic transparency, making interpretation difficult for regulatory agencies and clinicians. In addition, long-term chronic toxicity and environmental nanotoxicity remain insufficiently understood. The adoption of explainable AI frameworks may improve regulatory confidence by providing mechanistic insights into nanoparticle-induced toxicity rather than relying solely on black-box predictions. Future development of AI-assisted nanotoxicology will require internationally standardized databases, explainable AI frameworks, and closer collaboration among computational scientists, toxicologists, clinicians, and regulatory organizations to ensure reliable and clinically relevant safety prediction. Table 4 highlights the expanding role of artificial intelligence in the development and optimization of smart nano-antibiotic systems. The table demonstrates how AI-driven computational approaches support nanomaterial prediction, therapeutic optimization, toxicity assessment, and precision antimicrobial design through advanced data analysis and predictive modeling. The integration of artificial intelligence with antimicrobial nanotechnology is accelerating the development of safer, more efficient, and personalized therapeutic platforms for combating antimicrobial resistance.

### 4.5. Integration of Multi-Omics Data

The integration of multi-omics data with artificial intelligence is rapidly emerging as a powerful strategy for advancing precision nano-antibiotic therapy. Infectious diseases are biologically complex processes involving dynamic interactions among microbial genomes, host immune responses, metabolic pathways, proteomic signaling, and environmental factors [114]. Conventional antimicrobial approaches often focus on isolated biological targets and therefore fail to fully capture this complexity. Multi-omics technologies including genomics, transcriptomics, proteomics, metabolomics, lipidomics, and microbiomics generate enormous amounts of biological data capable of revealing comprehensive molecular insights into infection progression and therapeutic response. AI-assisted integration of these datasets provides unprecedented opportunities for designing personalized smart nano-antibiotics tailored to specific pathogens, resistance mechanisms, and patient biological profiles. Advanced nanobiosensing platforms combined with microfluidic technologies have shown significant potential for rapid and sensitive detection of multiple bacterial pathogens, supporting future intelligent antimicrobial management systems [115]. The multi-omics integration may become one of the defining technological foundations of next-generation precision antimicrobial medicine. Genomic and transcriptomic data are particularly important for identifying microbial resistance genes, virulence pathways, and infection-specific molecular signatures [116]. AI algorithms can analyze sequencing datasets to predict antimicrobial susceptibility patterns and identify optimal therapeutic targets for nanoparticle-based interventions. Machine learning models are increasingly capable of correlating genomic mutations with resistance evolution and nanoparticle sensitivity. Transcriptomic profiling additionally enables monitoring of bacterial stress responses following nano-antibiotic exposure, thereby helping researchers optimize therapeutic strategies. A simplified omics integration framework may conceptually be represented as:

*Precision Therapy* = *f*(*Genomics* + *Proteomics* + *Metabolomics*)

The integrating molecular-level pathogen information with smart nanotechnology could dramatically improve antimicrobial specificity and reduce unnecessary broad-spectrum antibiotic exposure. Proteomics and metabolomics further expand understanding of host–pathogen interactions and therapeutic response mechanisms. Proteomic analysis can identify infection-associated enzymes, inflammatory mediators, membrane proteins, and biofilm-associated factors that may serve as triggers or targets for stimuli-responsive nanoplatforms. Metabolomic profiling provides insight into microbial metabolic adaptation, oxidative stress pathways, and local infection microenvironments. AI-assisted interpretation of these complex datasets enables identification of predictive biomarkers associated with therapeutic response, toxicity risk, and infection progression [117]. Such information can guide the rational design of nanoplatforms responsive to specific biochemical conditions including enzyme secretion, ROS generation, pH variation, or metabolic imbalance. The multi-omics-guided nanoplatform engineering represents a major advancement because it allows therapeutic systems to be designed around actual biological conditions rather than generalized assumptions. Microbiome analysis is another increasingly important area in precision antimicrobial therapy. Conventional broad-spectrum antibiotics often disrupt beneficial microbial communities, contributing to dysbiosis, immune imbalance, and secondary infections [118]. AI-integrated microbiome analysis can identify patient-specific microbial compositions and predict how nano-antibiotic therapies may influence microbial ecology. Such information may support development of selective antimicrobial nanoplatforms that preserve beneficial microbiota while targeting pathogenic organisms. Additionally, microbiome-derived biomarkers may assist in monitoring therapeutic response and predicting infection recurrence. The preserving microbiome stability will become an increasingly important objective in future antimicrobial nanomedicine. AI-driven multi-omics integration also supports adaptive and personalized therapeutic decision-making. Deep learning algorithms can process complex multidimensional datasets that would be impossible to interpret manually [116]. These computational systems can identify hidden biological correlations, classify infection subtypes, predict treatment outcomes, and recommend optimized nanoparticle formulations based on individual patient profiles. In advanced precision medicine models, multi-omics data may eventually be combined with biosensor feedback and real-time clinical monitoring to create self-adjusting nano-antibiotic systems capable of dynamically adapting therapy according to disease progression. Ultimate such intelligent therapeutic ecosystems represent one of the most exciting future directions in AI-guided antimicrobial nanotechnology. Nevertheless, significant challenges remain in implementing multi-omics integration for clinical nano-antibiotic development. Omics datasets are extremely large, heterogeneous, and computationally demanding, requiring advanced infrastructure and sophisticated analytical algorithms [119]. Data standardization, privacy concerns, interpretability limitations, and high analytical cost also restrict widespread clinical application. Several recent studies have demonstrated the practical utility of AI in nanomedicine development. Machine learning-assisted models have been successfully employed to predict nanoparticle toxicity, optimize drug-loading efficiency, and estimate biological responses based on physicochemical characteristics. These findings suggest that AI can serve as a valuable decision-support tool for accelerating nanomaterial development and improving formulation success rates prior to experimental validation [120,121]. Furthermore, translating omics-derived predictions into reproducible therapeutic outcomes remains scientifically challenging due to biological variability among patients and pathogens. Future progress will depend on the establishment of integrated biomedical databases, explainable AI platforms, and interdisciplinary collaboration capable of transforming complex molecular data into clinically actionable precision nanomedicine strategies.

## 5. Targeted and Precision Antimicrobial Therapy

The combined advances in stimuli-responsive nanotechnology and AI-assisted design provide new opportunities for precision antimicrobial interventions. Building upon the smart nano-antibiotic platforms discussed in previous sections, targeted therapeutic strategies can improve infection-site specificity, enhance treatment efficacy, and reduce unintended systemic effects. Precision antimicrobial therapy aims to optimize treatment efficacy by tailoring therapeutic interventions according to pathogen characteristics, infection microenvironment, and host-specific biological factors [122].

### 5.1. Infection Site Targeting Strategies

Targeted antimicrobial delivery has become a central objective in modern nanomedicine because conventional antibiotics often distribute nonspecifically throughout the body, leading to reduced therapeutic efficiency, systemic toxicity, and incomplete eradication of localized infections. Smart nano-antibiotics are specifically engineered to improve accumulation at infected tissues while minimizing unnecessary exposure to healthy organs. Such targeted delivery systems enhance local drug concentration, improve intracellular penetration, reduce dosing frequency, and suppress the emergence of antimicrobial resistance associated with prolonged subtherapeutic exposure [123]. In precision antimicrobial therapy, targeting strategies are designed not only to transport antimicrobial agents efficiently but also to recognize unique biological characteristics of infected microenvironments. The infection-targeted nanotherapy represents one of the most important conceptual transitions in antimicrobial medicine because it changes treatment from generalized systemic exposure toward localized and biologically guided intervention. Passive targeting is among the earliest approaches used in antimicrobial nanoplatforms and primarily depends on physicochemical accumulation within inflamed or infected tissues [124]. Infection-associated inflammation often increases vascular permeability and disrupts endothelial integrity, allowing nanoscale materials to accumulate more effectively through enhanced permeation and retention-like effects. Nanoparticles with optimized size, surface charge, and hydrophilicity can preferentially localize within inflamed tissues due to altered vascular architecture and impaired lymphatic clearance. Polyethylene glycol (PEG) coatings are frequently employed to prolong circulation time and reduce rapid immune clearance, thereby improving nanoparticle accumulation at infection sites. However, passive targeting alone may not provide sufficient specificity in complex infections because inflammatory conditions vary substantially among patients and disease stages. The passive targeting serves as a useful foundational strategy, but future precision antimicrobial systems will increasingly require active biological recognition mechanisms for improved therapeutic accuracy. Active targeting strategies involve surface functionalization of nanoparticles with ligands capable of recognizing infection-associated molecular markers. These ligands may include antibodies, antimicrobial peptides, aptamers, carbohydrates, folic acid, mannose derivatives, or receptor-specific molecules that selectively bind bacterial cells, infected tissues, or activated immune cells. Ligand-receptor interaction may conceptually be represented as:

*Ligand* + *Receptor* ⇌ *Ligand* − *Receptor Complex*

Such molecular recognition enhances nanoparticle adhesion, internalization, and retention within infected microenvironments. Mannose-functionalized nanoparticles, for example, have shown improved targeting toward macrophages harboring intracellular pathogens because macrophages express mannose receptors during immune activation. Similarly, antibody-conjugated nanocarriers can selectively recognize bacterial surface antigens and biofilm-associated proteins. Active targeting significantly improves therapeutic precision because it allows nano-antibiotics to interact directly with biological signatures associated with infection rather than relying solely on passive accumulation. Stimuli-responsive targeting strategies further enhance infection specificity by activating therapeutic functions only under pathological conditions. Infection sites frequently exhibit acidic pH, elevated enzyme activity, oxidative stress, hypoxia, and localized temperature changes. Smart nanoplatforms engineered with pH-sensitive polymers, enzyme-cleavable linkers, ROS-responsive materials, or thermosensitive components can selectively release antimicrobial agents under these conditions [94]. Such systems reduce premature drug leakage and improve localized therapeutic concentration. In several advanced designs, external triggers including near-infrared light, ultrasound, magnetic fields, or electrical stimulation are additionally employed to regulate nanoparticle activation with high spatial and temporal precision. The combining biological responsiveness with externally controllable activation may represent the future of adaptive antimicrobial therapy, particularly in difficult-to-access infections and implant-associated diseases. Cell membrane-coated nanoparticles and biomimetic targeting systems have also emerged as promising approaches in precision antimicrobial nanomedicine. These systems utilize natural cell membranes derived from immune cells, erythrocytes, platelets, or bacterial vesicles to camouflage nanoparticles and improve biological compatibility. Biomimetic coatings enhance immune evasion, prolong circulation, and facilitate interaction with infection-associated tissues [125]. Macrophage membrane-coated nanoparticles, for instance, exhibit improved accumulation within inflammatory environments because they inherit natural immune targeting properties from source cells. Such biomimetic systems may additionally neutralize bacterial toxins and inflammatory mediators while simultaneously delivering antimicrobial agents. A major challenge in treating persistent infections is the presence of bacteria within intracellular compartments such as phagosomes, where pathogens can evade immune surveillance and conventional antibiotics. To address this issue, nanotechnology platforms can be engineered with targeting ligands, cell-penetrating peptides, or biomimetic surface coatings that facilitate selective uptake by infected cells. In addition, stimuli-responsive nanocarriers capable of responding to intracellular pH changes, enzymatic activity, or oxidative stress may enable localized drug release within pathogen-containing compartments. Such strategies can improve intracellular antimicrobial delivery while minimizing exposure of healthy cells to high drug concentrations. The biomimetic targeting reflects a significant evolution in nanomedicine because it integrates synthetic nanotechnology with naturally evolved biological recognition mechanisms. Despite substantial progress, several limitations continue to challenge infection-targeted nano-antibiotic therapy. Heterogeneity of infection microenvironments, variability in receptor expression, immune clearance, and nonspecific protein adsorption can reduce targeting efficiency [126]. In addition, large-scale reproducibility and regulatory validation of functionalized nanoparticles remain difficult. Future development of targeted antimicrobial nanoplatforms will increasingly rely on AI-assisted optimization, multi-omics-guided biomarker selection, and real-time biosensing systems capable of dynamically adapting targeting behavior according to patient-specific infection characteristics.

### 5.2. Biofilm Penetration and Disruption

Biofilm-associated infections represent one of the most difficult challenges in antimicrobial therapy because biofilms create protective microbial communities that resist both antibiotic treatment and host immune defense mechanisms. Biofilms are structured aggregates of microorganisms embedded within extracellular polymeric substances composed primarily of polysaccharides, proteins, extracellular DNA, and lipids [127]. These matrices create physical and biochemical barriers that severely restrict antibiotic diffusion, reduce oxygen availability, alter microbial metabolic activity, and facilitate horizontal gene transfer associated with antimicrobial resistance. As a result, bacteria residing within biofilms can exhibit resistance levels hundreds to thousands of times greater than their planktonic counterparts. Conventional antibiotics often fail to eradicate mature biofilms completely, leading to chronic infections, implant contamination, recurrent inflammation, and therapeutic failure. The effective biofilm penetration and disruption may ultimately determine the clinical success of next-generation smart nano-antibiotics. Nanotechnology provides several important advantages for overcoming biofilm-associated resistance barriers. Due to their nanoscale dimensions, smart nano-antibiotics can penetrate dense extracellular polymeric matrices more effectively than conventional antibiotics [128]. Surface charge engineering plays a particularly important role because positively charged nanoparticles can interact strongly with negatively charged biofilm components, thereby improving retention and penetration within microbial communities. Certain nanoparticles additionally undergo pH-triggered or enzyme-triggered surface charge conversion after entering acidic biofilm environments, further enhancing diffusion and bacterial interaction. Polymeric nanoparticles, lipid-based nanocarriers, metallic nanostructures, and dendrimers have all demonstrated enhanced penetration capabilities in experimental biofilm models. Similarly, enhanced biofilm targeting can be achieved through surface-modified nanoparticles designed to penetrate the extracellular matrix and release antimicrobial agents in response to infection-specific signals. This selective approach improves bacterial eradication within protected niches while reducing damage to surrounding host tissues. The nanoscale mobility combined with tunable surface chemistry provides a major therapeutic advantage in targeting structurally complex biofilm environments. One of the primary mechanisms of biofilm disruption involves degradation of extracellular polymeric substances. Smart nanoplatforms can incorporate enzymes such as DNase, proteases, dispersin B, or polysaccharide-degrading agents capable of destabilizing biofilm architecture [129]. Enzyme-responsive nanocarriers may additionally release antimicrobial agents selectively after encountering biofilm-associated enzymes. In some advanced systems, nanoparticles generate reactive oxygen species that oxidatively damage extracellular matrices and bacterial membranes simultaneously. ROS-mediated oxidative stress may be simplified as:

*ROS* + *Biomolecules* → *Oxidative Damage*

**Figure 5:**
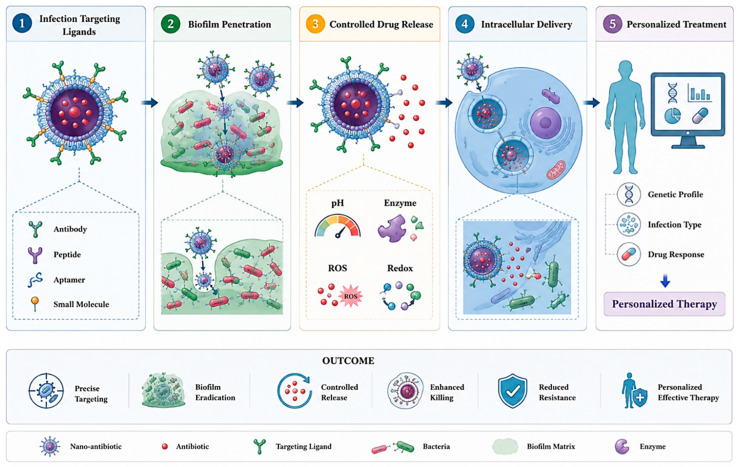
Schematic presentation of Targeted Precision Antimicrobial Therapy Using Smart Nano-Antibiotics and their outcomes.

Photothermal nanomaterials including gold nanoparticles, graphene oxide, and carbon nanotubes can further induce localized thermal disruption of biofilm structures under near-infrared irradiation. Such synergistic strategies improve antibiotic penetration while weakening microbial defense barriers. Ultimate, combining physical, chemical, and enzymatic disruption mechanisms within a single nanoplatform offers a effective strategy for eliminating mature resistant biofilms. Quorum sensing inhibition is another important approach in biofilm-targeted nanotherapy. Quorum sensing refers to bacterial communication systems that regulate biofilm maturation, virulence factor production, and collective microbial behavior [130]. Smart nanocarriers loaded with quorum sensing inhibitors can interfere with microbial signaling pathways, thereby preventing biofilm formation or destabilizing existing biofilm communities. Some nanoparticles additionally act as signaling decoys by adsorbing bacterial communication molecules and reducing coordinated microbial behavior. Combining quorum sensing inhibition with antibiotic delivery often produces synergistic antimicrobial effects because weakened biofilms become more susceptible to therapeutic penetration. The targeting bacterial communication pathways represents a promising complementary strategy for future precision antimicrobial therapy. Another major advantage of nano-antibiotics in biofilm treatment is their ability to improve intracellular and deep-layer antimicrobial delivery. Biofilms frequently contain metabolically dormant persister cells that exhibit high tolerance toward conventional antibiotics [131]. Smart nanoplatforms capable of sustained release, stimuli-responsive activation, or external trigger responsiveness can maintain prolonged antimicrobial pressure within deep biofilm regions where conventional antibiotics rapidly lose activity. Multifunctional nanoplatforms may also combine photothermal therapy, magnetic targeting, nitric oxide release, and immune modulation to enhance biofilm eradication efficiency. AI-assisted optimization of nanoparticle size, surface chemistry, and release kinetics further improves penetration behavior and therapeutic precision. The development of predictive models that accurately represent biofilm-associated infections is essential for improving the design of nano-antibiotic systems. Future modeling approaches should incorporate key biofilm characteristics, including spatial heterogeneity, extracellular polymeric matrix composition, nutrient gradients, bacterial metabolic diversity, and interspecies interactions. Integration of artificial intelligence with imaging data, multi-omics analyses, and advanced in vitro biofilm models may provide a more realistic representation of biofilm behavior and treatment response. Such models could facilitate the rational optimization of nanomaterial properties for enhanced penetration, retention, and antimicrobial activity within resistant biofilm communities. The integration of AI with antibiofilm nanotechnology could significantly accelerate development of adaptive therapeutic systems capable of responding dynamically to heterogeneous biofilm microenvironments. Despite encouraging progress, important challenges remain in translating biofilm-targeted nano-antibiotics into clinical practice. Dense biofilm heterogeneity, limited oxygen diffusion, immune interaction, and nanoparticle aggregation may reduce penetration efficiency in vivo. Long-term biosafety and controlled degradation of antibiofilm nanomaterials also require careful evaluation. Furthermore, excessive ROS generation or thermal disruption may damage surrounding healthy tissues if not properly controlled. Future success in biofilm-targeted antimicrobial nanomedicine will depend on developing multifunctional, biocompatible, and precisely regulated nanoplatforms capable of achieving complete biofilm eradication without compromising host tissue integrity. Figure 5 illustrates targeted precision antimicrobial therapy using smart nano-antibiotic systems against resistant bacterial infections. The schematic highlights selective infection-site targeting, biofilm penetration, controlled drug release, and enhanced intracellular delivery through surface-functionalized nanocarriers. These intelligent nanoplatforms improve therapeutic efficiency while reducing systemic toxicity and the risk of antimicrobial resistance development.

### 5.3. Controlled and Sustained Drug Release

Controlled and sustained drug release is one of the most important functional advantages of smart nano-antibiotics because it directly influences therapeutic concentration, antimicrobial efficacy, dosing frequency, and systemic safety. Conventional antibiotics frequently exhibit rapid systemic distribution and short biological half-lives, often requiring repeated administration to maintain effective therapeutic levels. Such fluctuating drug concentrations may result in subtherapeutic exposure, incomplete bacterial eradication, and accelerated emergence of antimicrobial resistance [132]. In contrast, smart nanoplatforms are engineered to regulate the spatial and temporal release of antimicrobial agents through programmable nanoscale architectures and stimuli-responsive mechanisms. This capability enables prolonged therapeutic action, improved infection-site retention, and reduction of systemic toxicity. The controlled release technology represents one of the most clinically transformative aspects of nano-antibiotics because therapeutic failure is often linked not to lack of antimicrobial potency, but to inefficient and poorly regulated drug delivery. The release behavior of nano-antibiotics depends strongly on nanocarrier composition, polymer degradation, diffusion kinetics, surface chemistry, and environmental responsiveness. Polymeric nanoparticles, liposomes, hydrogels, dendrimers, and mesoporous nanostructures are commonly designed to provide sustained antimicrobial release over extended periods. Drug release may occur through diffusion, matrix erosion, swelling, enzymatic degradation, or external activation depending on the structural characteristics of the nanoplatform [57]. Controlled release kinetics are frequently modeled using diffusion-based equations such as:

Mt/M∞ = kt^1/2^

where Mt/M∞ represents the fraction of drug released over time and k denotes the kinetic release constant. Such mathematical modeling allows optimization of release profiles according to infection severity and therapeutic objectives. The kinetic control is essential because antimicrobial effectiveness depends not only on drug concentration, but also on maintaining sufficient exposure duration at infected tissues. Stimuli-responsive nanoplatforms provide additional sophistication by enabling infection-triggered release under specific pathological conditions. Acidic pH, bacterial enzymes, oxidative stress, hypoxia, and local temperature elevation associated with infections can selectively activate antimicrobial release from smart carriers. This responsive behavior minimizes premature drug leakage during systemic circulation while maximizing therapeutic concentration at infection sites. For example, pH-sensitive polymeric nanoparticles may remain stable at physiological pH but rapidly release antibiotics within acidic biofilm environments. Enzyme-responsive systems similarly exploit bacterial proteases or lipases to trigger localized drug liberation. External stimuli such as near-infrared light, ultrasound, and magnetic fields further allow clinicians to regulate antimicrobial release with high spatial precision. The integrating sustained delivery with environmental responsiveness creates a adaptive therapeutic system capable of responding dynamically to evolving infection conditions. Controlled release systems are especially valuable for treating chronic infections and biofilm-associated diseases where prolonged antimicrobial exposure is necessary. Biofilms frequently contain dormant persister cells that survive transient antibiotic exposure and contribute to recurrent infection. Sustained nano-antibiotic release can maintain effective antimicrobial concentrations within deep biofilm regions for extended durations, thereby improving eradication efficiency. Injectable hydrogels and implantable nanocomposite systems have shown particular promise in orthopedic infections, wound healing, and implant-associated microbial colonization because they provide localized long-term antimicrobial delivery without repeated systemic dosing. Multifunctional nanoplatforms combining controlled release with photothermal therapy, ROS generation, or immune modulation further enhance therapeutic performance. The prolonged localized antimicrobial activity may become increasingly important as resistant chronic infections continue to rise globally. Another important advantage of controlled release nanotechnology involves reduction of systemic toxicity and improvement of patient compliance. Conventional antibiotics often require frequent dosing schedules that increase the risk of gastrointestinal disturbance, nephrotoxicity, hepatotoxicity, and microbiome disruption. Sustained-release nano-antibiotics reduce dosing frequency while maintaining more stable plasma and tissue drug levels. Such pharmacokinetic stability minimizes concentration peaks associated with toxicity and troughs associated with therapeutic failure [63]. Lipid-based nanocarriers and biodegradable polymeric systems additionally protect unstable antibiotics from premature degradation and improve bioavailability. The sustained-release smart nanoplatforms may significantly improve both therapeutic outcomes and patient adherence, particularly in long-duration antimicrobial treatments. Despite considerable progress, several challenges remain in optimizing controlled and sustained antimicrobial release systems. Achieving predictable release kinetics under variable biological conditions remains difficult due to differences in infection microenvironment, immune response, and tissue physiology [113]. Premature burst release, incomplete drug liberation, nanoparticle instability, and manufacturing reproducibility also present major translational limitations. Furthermore, prolonged nanoparticle retention may increase the risk of chronic tissue accumulation or inflammatory responses if biodegradation is not properly controlled. The future advances will likely depend on AI-assisted kinetic optimization, real-time biosensing integration, and personalized release programming capable of tailoring antimicrobial delivery according to individual patient infection dynamics.

### 5.4. Personalized Antimicrobial Therapy

Personalized antimicrobial therapy has emerged as an important concept in modern infectious disease management because microbial infections and patient responses exhibit substantial biological variability. Conventional antibiotic treatments are generally based on standardized dosing regimens and broad-spectrum therapeutic strategies that often fail to account for differences in pathogen resistance profiles, host immune status, metabolic conditions, microbiome composition, and infection microenvironment [133]. Such generalized approaches contribute to therapeutic failure, unnecessary toxicity, microbiome disruption, and accelerated antimicrobial resistance development. Smart nano-antibiotics integrated with artificial intelligence, biosensing technologies, and multi-omics analysis provide a powerful framework for developing personalized antimicrobial therapies tailored to individual patient characteristics. The precision-guided antimicrobial nanomedicine represents a major evolution in infectious disease treatment because it shifts clinical focus from population-based therapy toward individualized therapeutic optimization. One of the fundamental aspects of personalized antimicrobial therapy involves pathogen-specific targeting and resistance profiling. Advances in genomics and AI-assisted microbial analysis now allow rapid identification of bacterial species, resistance genes, virulence factors, and metabolic signatures associated with infection progression. Smart nanoplatforms can therefore be engineered to deliver antibiotics selectively against specific pathogens or resistance mechanisms [15]. Surface-functionalized nanoparticles containing antibodies, aptamers, antimicrobial peptides, or receptor-specific ligands enhance recognition of particular microbial strains while minimizing effects on beneficial microbiota. Such targeted precision reduces unnecessary broad-spectrum antibiotic exposure and may help preserve microbial ecological balance. The pathogen-specific nanotherapy may become increasingly important in combating multidrug-resistant infections where conventional empirical treatments often fail. Personalized therapy also involves adapting antimicrobial release behavior according to patient-specific biological environments [134]. Infection-associated pH, oxidative stress, enzyme activity, oxygen availability, and inflammatory signaling vary substantially among patients and disease stages. Stimuli-responsive nanoplatforms capable of sensing these biological conditions can regulate antimicrobial release dynamically in response to local pathological cues. AI-driven predictive systems further optimize nanoparticle composition, release kinetics, and targeting behavior using patient-derived biological data. Such adaptive therapeutic control improves treatment precision while reducing systemic toxicity. The combining biosensing technologies with smart nanocarriers may eventually allow real-time therapeutic adjustment according to continuously changing infection conditions. Multi-omics integration plays a major role in personalized nano-antibiotic therapy because it enables comprehensive understanding of host–pathogen interactions at molecular levels [119]. Genomic, transcriptomic, proteomic, metabolomic, and microbiome analyses provide detailed information regarding immune response, microbial adaptation, inflammatory pathways, and therapeutic susceptibility. AI algorithms can process these multidimensional datasets to predict therapeutic response and identify optimal nano-antibiotic formulations for individual patients. Such precision-guided strategies are particularly valuable in chronic infections, immunocompromised patients, and polymicrobial diseases where biological complexity is exceptionally high. The integration of omics technologies with smart nanomedicine may ultimately redefine infectious disease management by enabling truly individualized therapeutic intervention. Another important aspect of personalized antimicrobial nanotherapy involves minimizing patient-specific toxicity risk [46]. Biological responses to nanoparticles vary depending on age, genetics, immune condition, metabolic profile, organ function, and existing comorbidities. AI-assisted toxicity prediction models can analyze patient-specific clinical and molecular data to estimate potential adverse responses before therapeutic administration. Such computational safety assessment improves formulation selection and dosage optimization while reducing the probability of severe side effects. Personalized biodistribution prediction may additionally help optimize nanoparticle size, surface chemistry, and degradability for individual physiological conditions. The individualized safety optimization equally important as antimicrobial effectiveness because therapeutic success requires maintaining a careful balance between pathogen eradication and host tissue compatibility. Emerging technologies such as wearable biosensors, implantable monitoring devices, and real-time infection tracking systems are further expanding the possibilities of personalized antimicrobial therapy [135]. Smart nanoplatforms integrated with biosensors may eventually allow continuous monitoring of inflammatory biomarkers, microbial load, oxidative stress, or local pH changes during treatment. AI systems could then dynamically adjust drug release behavior according to therapeutic response. Such closed-loop therapeutic systems would represent a major advancement toward autonomous precision medicine. The future of antimicrobial nanotechnology may increasingly involve intelligent therapeutic ecosystems in which diagnosis, monitoring, targeting, and adaptive treatment function together within integrated personalized platforms. Despite remarkable potential, personalized antimicrobial nanotherapy still faces several important challenges. High analytical cost, limited clinical infrastructure, data privacy concerns, regulatory complexity, and lack of standardized biological databases currently restrict widespread implementation. Additionally, integrating large-scale omics data and real-time biosensing information into clinically practical treatment systems remains technically demanding. The future progress will depend on interdisciplinary collaboration among nanotechnologists, clinicians, microbiologists, computational scientists, and regulatory agencies to establish clinically reliable and economically accessible precision antimicrobial platforms capable of addressing the growing global burden of resistant infections.

## 6. Hybrid and Advanced Nano-Antibiotic Systems

### 6.1. Metal-Based Nanoparticles (Ag, ZnO, Etc.)

Metal-based nanoparticles are among the most extensively investigated nanomaterials in antimicrobial nanomedicine because of their strong intrinsic antimicrobial activity, broad-spectrum effectiveness, and multifunctional therapeutic potential [34]. Unlike conventional antibiotics that generally target specific microbial pathways, metallic nanoparticles often exert simultaneous physicochemical and biochemical damage against bacterial cells, thereby reducing the probability of rapid resistance development. Their nanoscale dimensions provide exceptionally high surface-area-to-volume ratios, enabling enhanced interaction with microbial membranes, intracellular structures, and biofilm matrices. Smart metal-based nano-antibiotics are increasingly engineered with stimuli-responsive coatings, targeting ligands, and controlled release mechanisms to improve therapeutic specificity and biosafety. The metallic nanoplatforms represent one of the most powerful antimicrobial technologies currently available because they combine direct bactericidal activity with adaptable nanomedical functionality. Silver nanoparticles (AgNPs) are the most widely studied metal-based antimicrobial nanomaterials due to their potent broad-spectrum activity against Gram-positive bacteria, Gram-negative bacteria, fungi, and certain viruses [52]. The antimicrobial action of AgNPs involves multiple synergistic mechanisms including membrane disruption, reactive oxygen species generation, protein denaturation, enzyme inhibition, and DNA damage. Silver ions released from nanoparticle surfaces can strongly interact with sulfur-containing proteins and phosphate groups in nucleic acids, leading to metabolic dysfunction and inhibition of microbial replication. Ion release behavior may be simplified as:

*Ag* → *Ag*^+^ + *e*^−^

The released silver ions subsequently participate in oxidative and structural cellular damage pathways. AgNPs have demonstrated remarkable activity against multidrug-resistant pathogens and biofilm-associated infections because their mechanisms are nonspecific and difficult for bacteria to evade through simple mutational adaptation. The silver-based nano-antibiotics remain among the most clinically promising antimicrobial nanomaterials, particularly in wound healing, implant coatings, and topical infection management. Zinc oxide nanoparticles (ZnO NPs) have also gained substantial attention because of their photocatalytic antimicrobial activity, relative biocompatibility, and multifunctional therapeutic behavior [136]. ZnO nanoparticles generate reactive oxygen species under ultraviolet or visible light exposure, leading to oxidative damage of microbial membranes, proteins, and intracellular components. Additionally, released zinc ions can interfere with bacterial enzymatic systems and membrane integrity. ZnO nanomaterials possess advantages such as low cost, environmental stability, and favorable biosafety profiles compared with several other metallic nanostructures. In antimicrobial wound healing applications, ZnO nanoparticles additionally support tissue regeneration and anti-inflammatory activity. Surface-modified ZnO systems capable of pH-responsive or enzyme-responsive activation are increasingly investigated for precision antimicrobial therapy. The ZnO-based nanoplatforms are particularly attractive because they combine antimicrobial functionality with regenerative therapeutic potential. Gold nanoparticles (AuNPs) differ from silver and zinc oxide nanoparticles because their primary value lies less in intrinsic antimicrobial activity and more in their exceptional physicochemical tunability and multifunctionality [137]. AuNPs exhibit excellent biocompatibility, stable surface chemistry, and controllable optical properties, making them ideal platforms for targeted antimicrobial delivery and photothermal therapy. Surface conjugation with antibiotics, antimicrobial peptides, antibodies, aptamers, or nucleic acids significantly enhances pathogen-specific targeting. Under near-infrared irradiation, AuNPs efficiently convert optical energy into localized heat capable of disrupting bacterial membranes and biofilm structures. Such photothermal activation enables precise external control of antimicrobial therapy. The gold nanotechnology may play a major role in future theranostic antimicrobial systems where diagnosis, targeting, and therapy are integrated within a single multifunctional platform. Other important metallic nanomaterials include copper oxide, titanium dioxide, magnesium oxide, and iron oxide nanoparticles [138]. Copper oxide nanoparticles exhibit strong oxidative antimicrobial activity and can effectively disrupt resistant bacterial membranes; however, their potential cytotoxicity requires careful dose optimization. Titanium dioxide nanoparticles are widely explored in photocatalytic antimicrobial systems due to their ability to generate ROS under light activation. Iron oxide nanoparticles provide additional advantages in magnetic targeting, imaging, and externally guided drug delivery [53]. Magnetically responsive systems can accumulate selectively at infected tissues under external magnetic fields while simultaneously enabling controlled drug release or hyperthermia-based therapy. The multifunctionality is becoming increasingly important in metallic nano-antibiotic design because future therapeutic systems will likely require simultaneous targeting, imaging, monitoring, and antimicrobial activity. Despite their significant therapeutic promise, metallic nanoparticles also present important translational challenges. Potential cytotoxicity, oxidative tissue injury, long-term organ accumulation, environmental persistence, and limited biodegradability remain major concerns. Excessive ROS generation may damage healthy tissues and provoke inflammatory responses if not carefully regulated. In addition, large-scale synthesis reproducibility and standardized safety evaluation remain technically challenging. Recent multifunctional nanoplatforms based on biocompatible materials have demonstrated efficient intracellular therapeutic delivery and favorable biosafety profiles, supporting the development of advanced hybrid nanocarrier systems [139]. In the future development of metallic nano-antibiotics will increasingly depend on advanced surface engineering, biodegradable hybrid structures, AI-assisted toxicity prediction, and precision-controlled therapeutic activation capable of balancing antimicrobial potency with long-term biological safety.

### 6.2. Polymeric and Lipid Nanocarriers

Polymeric and lipid-based nanocarriers represent two of the most clinically adaptable and biocompatible platforms in smart antimicrobial nanomedicine. Unlike many metallic nanoparticles that rely primarily on intrinsic antimicrobial activity, polymeric and lipid nanocarriers are generally designed to improve drug delivery efficiency, therapeutic precision, pharmacokinetic stability, and stimuli-responsive release behavior [58]. These nanoplatforms are particularly valuable for encapsulating conventional antibiotics, antimicrobial peptides, nucleic acids, and combination therapeutics within controlled nanoscale architectures. Their structural versatility allows extensive surface modification, targeted delivery, and integration with responsive materials for precision antimicrobial therapy. The polymeric and lipid nanocarriers may ultimately achieve broader clinical translation than many inorganic nanomaterials because of their superior biodegradability, formulation flexibility, and safety profile. Polymeric nanocarriers are commonly fabricated using biodegradable natural or synthetic polymers capable of encapsulating therapeutic agents within nanoscale matrices. Frequently used materials include chitosan, alginate, gelatin, dextran, hyaluronic acid, poly(lactic-co-glycolic acid) (PLGA), polyethylene glycol (PEG), and polycaprolactone (PCL). These polymers provide excellent control over nanoparticle size, degradation behavior, surface functionality, and drug release kinetics [140]. Polymeric nanoparticles can encapsulate both hydrophilic and hydrophobic antimicrobial agents while protecting unstable drugs from premature degradation. Drug release from polymeric systems frequently occurs through diffusion, swelling, matrix erosion, or enzymatic degradation mechanisms. Controlled polymer degradation may conceptually be represented as:

Polymer + H_2_O → Degraded Fragments

The tunable biodegradation is one of the greatest strengths of polymeric nanotechnology because it allows customizable therapeutic release profiles according to infection severity and tissue environment. Chitosan-based nanoparticles have attracted particular attention due to the intrinsic antimicrobial and mucoadhesive properties of chitosan itself. Positively charged chitosan molecules interact strongly with negatively charged bacterial membranes, promoting membrane destabilization and enhanced intracellular antibiotic delivery. Chitosan nanocarriers additionally improve biofilm penetration and wound healing performance while exhibiting relatively favorable biosafety characteristics [141]. PLGA nanoparticles are similarly important because of their excellent biodegradability and regulatory acceptance in biomedical applications. PEGylation of polymeric nanoparticles further enhances systemic circulation stability by reducing protein adsorption and immune clearance. Stimuli-responsive polymeric systems capable of responding to pH, enzymes, ROS, or temperature changes are increasingly explored for infection-selective antimicrobial activation. The responsive polymeric nanocarriers provide a flexible platform for designing adaptive and patient-specific antimicrobial therapies. Lipid-based nanocarriers are equally important in precision antimicrobial medicine due to their excellent biocompatibility and membrane interaction properties [142]. Liposomes are among the earliest and most clinically established nanocarriers, consisting of phospholipid bilayer vesicles capable of encapsulating both hydrophilic and hydrophobic drugs. Liposomal encapsulation improves antibiotic stability, reduces systemic toxicity, prolongs circulation time, and enhances intracellular delivery. Several liposomal antimicrobial formulations have already achieved clinical approval, highlighting the translational potential of lipid nanotechnology. Solid lipid nanoparticles and nanostructured lipid carriers provide additional advantages such as improved physical stability and controlled release behavior compared with traditional liposomes. The lipid-based nanocarriers are particularly important because their membrane-mimicking characteristics allow efficient biological interaction with relatively low immunogenicity. Another important advantage of polymeric and lipid nanocarriers is their ability to support multifunctional and hybrid therapeutic systems. These platforms can co-deliver antibiotics, antimicrobial peptides, anti-inflammatory agents, quorum sensing inhibitors, or nucleic acid therapeutics within single nanoscale architectures. Surface functionalization with antibodies, peptides, aptamers, or biomimetic coatings further improves infection-site targeting and intracellular accumulation [54]. In advanced formulations, polymeric or lipid nanocarriers are combined with metallic nanoparticles, photothermal agents, biosensors, or AI-guided responsive systems to create adaptive therapeutic nanoplatforms. The future of antimicrobial nanomedicine will likely involve hybrid multifunctional systems rather than isolated single-material approaches. Despite their promising therapeutic potential, polymeric and lipid nanocarriers still face important translational challenges. Drug leakage during storage, limited long-term stability, manufacturing complexity, sterilization difficulty, and scalability issues remain significant concerns. In some cases, rapid clearance by the mononuclear phagocyte system may reduce therapeutic efficiency. Furthermore, maintaining reproducible release kinetics under variable physiological conditions remains difficult. In the future advancement of polymeric and lipid nano-antibiotics will depend heavily on AI-assisted formulation optimization, advanced biomaterials engineering, and precision manufacturing technologies capable of improving reproducibility, stability, and clinical reliability.

### 6.3. Nano-Drug Combination Systems

Nano-drug combination systems represent an advanced therapeutic strategy in antimicrobial nanomedicine where nanomaterials are integrated with antibiotics, antimicrobial peptides, phytochemicals, nucleic acids, or multiple therapeutic agents to achieve synergistic antimicrobial activity. Conventional monotherapy approaches often fail against multidrug-resistant pathogens because bacteria rapidly adapt through mutation, biofilm formation, efflux pump activation, and metabolic reprogramming [143]. Combination therapy aims to overcome these limitations by attacking microbial cells through multiple simultaneous mechanisms, thereby reducing the probability of resistance development and improving therapeutic efficacy. Nanotechnology further enhances this strategy by improving drug stability, targeted delivery, controlled release, and intracellular penetration. The nano-drug combination systems are particularly promising because they integrate the advantages of conventional antimicrobial pharmacology with the multifunctionality of smart nanoplatforms. One of the most common approaches involves combining metallic nanoparticles with conventional antibiotics to enhance bactericidal efficiency. Silver nanoparticles, zinc oxide nanoparticles, and copper oxide nanoparticles have demonstrated strong synergistic interactions with antibiotics such as ciprofloxacin, vancomycin, ampicillin, tetracycline, and gentamicin. Metallic nanoparticles increase bacterial membrane permeability and disrupt cellular integrity, thereby facilitating deeper intracellular antibiotic penetration. Simultaneously, released metal ions and reactive oxygen species create oxidative stress that weakens microbial defense systems. Synergistic interaction may conceptually be represented as:

*Effect_combined_* > *Effect_nano_* + *Effect_drug_*

Such synergistic enhancement frequently allows reduction of antibiotic dosage while maintaining strong antimicrobial potency. The dosage reduction is important because it may minimize systemic toxicity and slow resistance evolution associated with excessive antibiotic exposure. Polymeric and lipid-based nanocarriers are also extensively used for combination antimicrobial delivery. These systems can simultaneously encapsulate multiple antibiotics or co-deliver antibiotics together with anti-inflammatory agents, antimicrobial peptides, quorum sensing inhibitors, or biofilm-disrupting molecules. Controlled co-release enables synchronized therapeutic action against multiple bacterial survival pathways. For example, one drug may destabilize the biofilm matrix while another directly eliminates bacterial cells. Similarly, anti-inflammatory compounds may reduce tissue damage while antimicrobial agents suppress infection progression [59]. Multifunctional polymeric systems additionally improve pharmacokinetic stability and prolong retention at infected tissues. The synchronized therapeutic delivery through smart nanocarriers represents a major advancement over traditional combination therapies where independent drug pharmacokinetics often remain poorly coordinated. Nano-drug combination systems are particularly valuable in treating biofilm-associated and intracellular infections. Biofilms significantly restrict antibiotic penetration and create metabolically heterogeneous microbial populations including resistant persister cells. Nanoparticles capable of penetrating extracellular polymeric matrices can transport antimicrobial agents deeply into biofilms while simultaneously releasing ROS-generating materials, enzymes, or photothermal agents to destabilize biofilm structure [144]. In intracellular infections, nanocarriers improve antibiotic delivery into macrophages and infected epithelial cells where conventional antibiotics often exhibit limited penetration. The multifunctional nanocarrier-mediated combination therapy may become essential for managing chronic and recurrent infections associated with resistant microbial communities. Another rapidly developing area involves combining nanotechnology with antimicrobial peptides, bacteriophages, and nucleic acid therapeutics. Antimicrobial peptides exhibit broad-spectrum membrane-disruptive activity but often suffer from instability and rapid degradation in physiological environments. Encapsulation within nanoparticles protects these molecules and improves targeted delivery. Similarly, bacteriophage-loaded nanoplatforms can selectively infect pathogenic bacteria while minimizing disruption of beneficial microbiota. Small interfering RNA (siRNA), CRISPR-associated systems, and antisense oligonucleotides are additionally being integrated into nanocarriers to suppress resistance genes and virulence pathways. AI-assisted optimization further improves co-loading efficiency, release sequencing, and therapeutic synergy. The integrating biological therapeutics with smart nanotechnology could fundamentally reshape the future landscape of precision antimicrobial medicine. Despite substantial therapeutic promise, nano-drug combination systems still face several translational challenges. Co-loading multiple agents with distinct physicochemical properties often complicates nanoparticle fabrication and release optimization [56]. Biomimetic nanoparticles derived from cellular membranes have demonstrated enhanced biological compatibility, prolonged circulation, and improved targeting capability, making them attractive candidates for next-generation antimicrobial nanomedicine [145]. Drug–drug interactions within nanocarriers may alter stability or reduce therapeutic activity. Maintaining synchronized release profiles under variable physiological conditions also remains difficult. Furthermore, increased formulation complexity may create additional regulatory and manufacturing barriers. In the future progress will depend on advanced materials engineering, AI-driven formulation design, and scalable precision manufacturing technologies capable of producing reproducible multifunctional nano-antibiotic systems suitable for clinical application.

### 6.4. Surface Functionalization and Ligand Targeting

Surface functionalization is one of the most critical engineering strategies in smart antimicrobial nanotechnology because the outer surface of nanoparticles largely determines their biological interaction, targeting efficiency, circulation stability, immune recognition, and therapeutic specificity. Bare nanoparticles frequently undergo rapid protein adsorption, aggregation, immune clearance, and nonspecific tissue interaction after systemic administration [146]. Surface modification therefore allows researchers to tailor nanoparticle behavior according to desired therapeutic objectives. Through functionalization with polymers, ligands, biomolecules, or responsive chemical groups, nano-antibiotics can achieve enhanced infection-site targeting, improved biofilm penetration, prolonged circulation, and controlled biological activation. The surface engineering transforms nanoparticles from passive drug carriers into biologically interactive therapeutic systems capable of selective antimicrobial action. Polymer coatings are among the most widely used surface modification approaches for improving nanoparticle stability and biocompatibility. Polyethylene glycol (PEG) is especially important because PEGylation reduces protein adsorption and minimizes recognition by the mononuclear phagocyte system, thereby prolonging systemic circulation time. Reduced immune clearance improves nanoparticle accumulation at infected tissues and enhances therapeutic efficiency. Surface hydration provided by PEG coatings also decreases nanoparticle aggregation under physiological conditions. Other polymers such as chitosan, dextran, hyaluronic acid, and zwitterionic materials are additionally used to improve mucoadhesion, cellular interaction, or stimuli responsiveness [147]. The polymeric surface engineering remains foundational for clinical nanomedicine because therapeutic efficacy is strongly dependent on maintaining nanoparticle stability within complex biological environments. Ligand targeting further enhances nanoparticle specificity by enabling selective recognition of bacterial cells, infected tissues, or activated immune cells. Targeting ligands may include antibodies, aptamers, peptides, carbohydrates, folic acid derivatives, mannose molecules, or receptor-specific biomolecules attached to nanoparticle surfaces through covalent or electrostatic interactions. Ligand-mediated targeting interaction may conceptually be represented as:

*Nanoparticle* − *Ligand* + *Target Receptor* → *Targeted Binding*

Such molecular recognition significantly improves nanoparticle localization and internalization at infection sites. Mannose-functionalized nanoparticles, for example, exhibit enhanced uptake by macrophages due to interaction with mannose receptors expressed during immune activation. Similarly, antibody-conjugated nanoplatforms can selectively recognize bacterial surface antigens and biofilm-associated proteins. The ligand-directed targeting is especially important for precision antimicrobial therapy because it minimizes unnecessary exposure to healthy tissues while improving pathogen-specific therapeutic concentration. Stimuli-responsive surface functionalization adds another level of therapeutic sophistication. Nanoparticle surfaces can be engineered to respond dynamically to infection-associated conditions such as acidic pH, elevated ROS levels, bacterial enzymes, or temperature changes [124]. For example, pH-responsive coatings may undergo protonation within acidic infection environments, causing surface charge reversal that improves bacterial adhesion and biofilm penetration. Enzyme-sensitive linkers can selectively expose targeting ligands or trigger drug release after encountering bacterial proteases or lipases. Such adaptive behavior significantly improves therapeutic precision and minimizes premature activation during systemic circulation. The environmentally responsive surface engineering may become central to the development of future intelligent nano-antibiotic systems capable of autonomous biological adaptation. Biomimetic functionalization strategies are also gaining major attention in advanced antimicrobial nanomedicine. These systems utilize natural biological membranes or biomolecular coatings derived from erythrocytes, platelets, macrophages, neutrophils, or bacterial vesicles to camouflage nanoparticles and improve physiological compatibility [148]. Cell membrane-coated nanoparticles inherit many natural biological functions from source cells, including immune evasion, inflammatory targeting, and toxin neutralization. Macrophage membrane-coated nanoparticles, for example, preferentially accumulate at inflammatory infection sites while simultaneously reducing immune clearance. Such biomimetic systems additionally decrease nonspecific toxicity and prolong circulation time. The biomimetic surface functionalization represents one of the advanced strategies currently being explored because it merges synthetic nanotechnology with naturally evolved cellular recognition mechanisms. Despite substantial progress, several limitations continue to affect surface-functionalized nano-antibiotics. Complex surface modification procedures may increase manufacturing cost and reduce large-scale reproducibility. Ligand density, orientation, and stability strongly influence targeting performance, yet maintaining consistent surface architecture remains technically challenging. Protein corona formation during systemic circulation may also mask targeting ligands and reduce therapeutic specificity. Furthermore, excessive surface functionalization may alter nanoparticle pharmacokinetics or increase immunogenicity. The future development of surface-engineered antimicrobial nanoplatforms will require AI-assisted optimization, standardized functionalization protocols, and advanced characterization methods capable of ensuring predictable and clinically reliable targeting performance. Table 5 presents various hybrid nano-antibiotic systems developed to enhance antimicrobial performance through synergistic therapeutic mechanisms. The table illustrates how combining different nanomaterials, antibiotics, polymers, peptides, or photothermal agents can improve targeting efficiency, controlled drug delivery, and antibacterial potency against resistant pathogens. These multifunctional hybrid platforms represent promising next-generation strategies for advanced precision antimicrobial therapy. Despite the encouraging therapeutic outcomes reported for many hybrid nano-antibiotic systems, most available evidence remains limited to in vitro investigations and preclinical animal studies. Clinical data evaluating long-term safety, therapeutic efficacy, large-scale manufacturing feasibility, and regulatory compliance are still scarce. Therefore, further translational research and well-designed clinical studies are required before these advanced multifunctional nanoplatforms can be considered for routine clinical application.

## 7. Challenges and Limitations

### 7.1. Toxicity and Biocompatibility Issues

Despite the remarkable therapeutic potential of smart nano-antibiotics, toxicity and biocompatibility concerns remain among the most significant barriers preventing widespread clinical translation. Nanomaterials possess unique physicochemical properties including extremely small size, large surface-area-to-volume ratio, high surface reactivity, and enhanced biological interaction capability. While these characteristics contribute to strong antimicrobial performance, they may also produce unintended adverse biological effects such as oxidative stress, inflammation, immune dysregulation, cellular damage, and long-term organ accumulation [149]. Unlike conventional antibiotics that are generally metabolized through well-characterized biochemical pathways, many nanomaterials interact dynamically with proteins, membranes, organelles, and immune systems in ways that remain incompletely understood. The ensuring long-term biological safety is equally as important as achieving antimicrobial efficacy because even effective nano-antibiotics cannot be clinically acceptable without predictable biocompatibility. One of the most frequently reported mechanisms of nanomaterial toxicity involves excessive generation of reactive oxygen species (ROS). Metallic nanoparticles such as silver, zinc oxide, copper oxide, and titanium dioxide can induce oxidative stress through catalytic surface reactions that damage lipids, proteins, mitochondria, and nucleic acids within host tissues. Although ROS generation contributes to antimicrobial activity, uncontrolled oxidative processes may simultaneously harm healthy mammalian cells. Oxidative stress-mediated injury may conceptually be represented as:

*ROS* + *Cellular Components* → *Oxidative Damage*

Excessive ROS exposure can initiate apoptosis, mitochondrial dysfunction, inflammatory signaling, and DNA fragmentation. Chronic oxidative stress may additionally contribute to tissue fibrosis and organ dysfunction following prolonged nanoparticle exposure. The future nano-antibiotic systems must achieve a careful balance between antimicrobial oxidative potency and host cellular protection. Nanoparticle size, morphology, and surface chemistry also strongly influence biocompatibility behavior [150]. Smaller nanoparticles often exhibit higher cellular uptake and deeper tissue penetration, but they may additionally cross biological barriers such as the blood–brain barrier and accumulate within sensitive organs including the liver, spleen, kidneys, lungs, and brain. Positively charged nanoparticles generally demonstrate enhanced bacterial interaction due to electrostatic attraction with negatively charged microbial membranes; however, they may also exhibit stronger interaction with mammalian cell membranes, increasing cytotoxicity and hemolytic risk. Surface coatings and functionalization strategies can partially improve biosafety by reducing nonspecific interaction and immune recognition. The rational surface engineering is essential for minimizing unintended biological damage while preserving antimicrobial efficiency. Immune system interaction represents another major challenge in antimicrobial nanomedicine. Nanoparticles may activate complement pathways, inflammatory cytokine release, macrophage recruitment, and adaptive immune responses depending on their composition and surface properties. Excessive immune activation can lead to inflammatory toxicity, hypersensitivity reactions, or rapid nanoparticle clearance that reduces therapeutic effectiveness [151]. Conversely, some nanomaterials may suppress immune function or alter normal immunological signaling pathways. Protein corona formation following nanoparticle exposure to biological fluids further complicates immune behavior by altering surface identity and cellular recognition patterns. The understanding immune-nanoparticle interaction is critically important because immune responses strongly influence both therapeutic efficacy and long-term biosafety. Long-term accumulation and biodegradability are also major concerns, particularly for inorganic nanomaterials with limited metabolic clearance. Certain metallic nanoparticles may persist within tissues for prolonged periods and undergo slow dissolution or transformation under physiological conditions. Chronic accumulation raises concerns regarding delayed toxicity, inflammatory tissue remodeling, and environmental persistence following excretion. Biodegradable polymeric and lipid-based systems generally exhibit improved safety profiles; however, degradation products themselves may sometimes induce local irritation or metabolic imbalance. Furthermore, repeated administration of nano-antibiotics could potentially alter microbiome composition and disrupt beneficial microbial ecosystems [63]. The biodegradability and controlled elimination among the most important future design criteria for clinically acceptable antimicrobial nanoplatforms. Another important limitation involves the lack of standardized toxicity assessment protocols for nanomedicine. Toxicological outcomes frequently vary across studies due to differences in synthesis methods, particle characterization, biological models, dosing conditions, and analytical techniques. Conventional toxicology assays are often insufficient for evaluating the dynamic and multifunctional behavior of nanomaterials. In vivo prediction remains especially challenging because biological responses are influenced by complex interactions among nanoparticles, proteins, immune systems, microbiota, and disease microenvironments. AI-assisted toxicity prediction and high-throughput screening technologies are increasingly being explored to improve safety evaluation efficiency and reproducibility. In addition to immediate biosafety concerns, the long-term fate of metallic nanoparticles remains an important consideration for both human health and environmental sustainability. Metallic nanomaterials such as silver, gold, zinc oxide, and copper-based nanoparticles may accumulate in organs including the liver, spleen, kidneys, and lungs following prolonged exposure, potentially leading to chronic oxidative stress, inflammatory responses, or alterations in cellular homeostasis. Furthermore, nanoparticles released through manufacturing processes, medical waste, or environmental disposal may persist in aquatic and terrestrial ecosystems, where they can interact with microorganisms, plants, and higher organisms. Such environmental accumulation may influence microbial community structure, nutrient cycling, and ecological balance. Therefore, future research should prioritize long-term biodistribution studies, biodegradability assessment, environmental monitoring, and the development of safer and more sustainable nano-antibiotic materials to support responsible clinical translation. The future progress in antimicrobial nanomedicine will depend heavily on internationally standardized nanotoxicology frameworks capable of generating reliable and clinically relevant safety data. Despite these challenges, substantial advances continue to improve the biosafety profile of smart nano-antibiotics. Surface modification, biomimetic coatings, biodegradable materials, stimuli-responsive activation, and precision-targeted delivery all contribute to reducing nonspecific toxicity [14]. AI-driven material optimization and personalized safety prediction may further improve clinical reliability in the future. The toxicity challenges should not be viewed as barriers preventing nanomedicine development, but rather as critical scientific problems that must be systematically addressed through interdisciplinary collaboration, advanced materials engineering, and rigorous long-term biological investigation.

### 7.2. Stability and Scalability

Stability and large-scale manufacturability remain major obstacles limiting the commercial and clinical translation of smart nano-antibiotic systems. Although numerous nanoplatforms demonstrate impressive antimicrobial performance under laboratory conditions, maintaining consistent physicochemical stability and reproducible functionality during long-term storage, transportation, and industrial production is considerably more challenging. Nano-antibiotics are sensitive systems whose therapeutic behavior depends strongly on particle size, morphology, surface chemistry, encapsulation efficiency, and colloidal stability [102]. Even minor alterations during manufacturing or storage may significantly affect drug release behavior, targeting capability, antimicrobial potency, and biological safety. The scalable reproducibility is one of the most critical requirements for future clinical implementation because therapeutic reliability cannot be achieved without stable and standardized manufacturing processes. Physicochemical instability is one of the most common problems encountered in antimicrobial nanomedicine. Nanoparticles often undergo aggregation, sedimentation, oxidation, hydrolysis, or premature drug leakage during storage due to their high surface energy and reactive interfaces. Aggregation may increase particle size, reduce effective surface area, alter biodistribution behavior, and decrease antimicrobial activity [152]. Surface oxidation can additionally modify nanoparticle reactivity and destabilize functional coatings or targeting ligands. Drug-loaded polymeric and lipid-based systems frequently experience gradual payload leakage over time, reducing therapeutic efficiency before administration. Colloidal instability may conceptually be represented as:

*Stable Nanoparticles* → *Aggregation* + *Sedimentation*

The long-term physicochemical stability is especially important for smart stimuli-responsive systems because uncontrolled structural changes may trigger unintended drug release or loss of responsiveness. Environmental factors such as temperature, pH, ionic strength, light exposure, and biological fluid interaction further complicate nanoparticle stability. Lipid-based nanocarriers are particularly susceptible to membrane fusion, lipid oxidation, and phase separation during storage. Polymeric nanoparticles may undergo hydrolytic degradation or structural swelling depending on environmental conditions [153]. Metallic nanoparticles can experience uncontrolled ion release or oxidation that alters antimicrobial potency and toxicity behavior. Maintaining stability under physiological conditions is equally important because protein adsorption and protein corona formation after systemic administration may significantly alter nanoparticle targeting efficiency and pharmacokinetics. The future nano-antibiotic development must prioritize environmental robustness alongside antimicrobial performance to ensure clinically reliable therapeutic behavior. Scalability presents another major translational challenge because many nanomaterial synthesis methods remain difficult to reproduce consistently at industrial levels. Laboratory-scale fabrication techniques often involve controlled conditions that become difficult to maintain during large-volume manufacturing. Variability in mixing rates, temperature control, solvent evaporation, reaction kinetics, and purification procedures can significantly affect nanoparticle size distribution, encapsulation efficiency, and surface functionality [154]. Batch-to-batch inconsistency is particularly problematic for multifunctional and stimuli-responsive systems where therapeutic performance depends on precise nanoscale architecture. The industrial scalability may become a decisive factor determining which nano-antibiotic technologies ultimately achieve commercial success. Complex multifunctional nanoplatforms also increase manufacturing difficulty and production cost. Systems incorporating targeting ligands, responsive polymers, imaging agents, multiple drugs, or biomimetic coatings often require multistep fabrication procedures involving sophisticated purification and characterization methods. Such complexity not only reduces manufacturing efficiency but also complicates regulatory evaluation and quality control. Sterilization presents additional challenges because conventional sterilization methods such as heat treatment, radiation, or filtration may damage nanoparticle structure or destabilize sensitive therapeutic components. The simplified yet multifunctional design strategies particularly important for improving translational feasibility and industrial adoption. Another important limitation involves insufficient standardization in nanomedicine characterization and regulatory assessment. Different research groups frequently employ inconsistent synthesis protocols, analytical methods, and reporting standards, making comparison among studies difficult. Parameters such as particle size distribution, zeta potential, morphology, encapsulation efficiency, release kinetics, and biodegradability are often measured using different methodologies [155]. This lack of harmonization complicates reproducibility and delays regulatory approval processes. AI-assisted process optimization and automated manufacturing technologies are increasingly being explored to improve production consistency and reduce variability. Future large-scale production of multi-stimuli responsive nano-antibiotics will require standardized manufacturing protocols, quality-by-design approaches, automated synthesis platforms, and AI-assisted process optimization to ensure batch-to-batch consistency while preserving stimulus-responsive functionality. Such strategies may significantly improve product stability, reproducibility, and industrial scalability. The integration of computational modeling, robotic synthesis, and real-time quality monitoring may significantly improve future nanomedicine scalability. Despite these challenges, considerable progress is being made toward improving the stability and manufacturability of antimicrobial nanoplatforms. Lyophilization techniques, advanced surface coatings, biodegradable stabilizers, and controlled atmosphere packaging have improved long-term storage performance for several nanomaterials. Continuous-flow synthesis systems and microfluidic manufacturing technologies additionally offer improved scalability and batch reproducibility compared with conventional bulk synthesis methods. AI-guided formulation optimization is further accelerating identification of stable nanoparticle compositions and scalable manufacturing conditions. The future success in antimicrobial nanomedicine will depend not only on scientific innovation but also on the development of economically feasible, industrially scalable, and regulatory-compliant production strategies capable of supporting widespread clinical implementation.

### 7.3. Regulatory and Ethical Considerations

Regulatory and ethical considerations represent major challenges in the clinical translation of smart nano-antibiotics because these systems combine complex nanomaterials, artificial intelligence, precision medicine technologies, and advanced biological interaction mechanisms within sophisticated therapeutic platforms. Conventional pharmaceutical regulatory frameworks were primarily developed for small-molecule drugs and biologics with relatively predictable physicochemical behavior. In contrast, nano-antibiotics often exhibit dynamic properties that change according to environmental conditions, surface modifications, biological interactions, and stimuli-responsive activation [60]. Such complexity creates substantial uncertainty regarding safety evaluation, manufacturing consistency, long-term toxicity assessment, and clinical validation. The regulatory advancement is now equally as important as scientific innovation because many promising antimicrobial nanotechnologies may fail to achieve clinical adoption without appropriate governance and standardized evaluation systems. One of the most significant regulatory difficulties involves the lack of internationally harmonized standards for nanomedicine characterization and safety assessment. Parameters such as particle size distribution, morphology, surface charge, aggregation behavior, biodegradability, and protein corona formation strongly influence therapeutic performance and toxicity profiles. However, different research laboratories and industrial manufacturers frequently use inconsistent synthesis methods, analytical techniques, and reporting standards. As a result, reproducibility among studies often remains limited, complicating regulatory comparison and clinical interpretation. Furthermore, small changes in nanoparticle formulation or surface chemistry may substantially alter biological behavior, making batch-to-batch consistency critically important. The establishing globally standardized characterization protocols is essential for ensuring regulatory confidence and facilitating broader clinical acceptance of nano-antibiotic technologies. Long-term safety assessment is another major regulatory concern. Many nano-antibiotics exhibit prolonged tissue retention, dynamic biodegradation pathways, or environmentally persistent behavior that cannot be fully evaluated using conventional toxicological testing frameworks [156]. Chronic exposure effects, immune modulation, reproductive toxicity, genotoxicity, and ecological impact remain insufficiently understood for many nanomaterials. Metallic nanoparticles are particularly concerning because certain systems may accumulate within organs or persist in environmental ecosystems after excretion. Regulatory agencies therefore require extensive preclinical and clinical safety evaluation before approving nanomedicine-based therapeutics. However, traditional toxicology models are often inadequate for capturing the complex biological interaction patterns of multifunctional nanoplatforms. The future regulatory science must evolve toward more dynamic and mechanism-based nanotoxicology evaluation strategies. The integration of artificial intelligence into nano-antibiotic design introduces additional ethical and regulatory complexities. AI systems are increasingly involved in nanoparticle optimization, toxicity prediction, drug release modeling, personalized therapeutic planning, and multi-omics interpretation. However, many advanced machine learning algorithms function as “black box” systems with limited transparency regarding how decisions are generated [157]. Lack of interpretability creates regulatory challenges because clinicians and regulatory authorities may be unable to fully understand or validate AI-generated therapeutic recommendations. Ethical concerns also arise regarding algorithmic bias, dataset imbalance, and unequal healthcare accessibility. If AI systems are trained primarily on limited population datasets, therapeutic predictions may become less reliable for underrepresented patient groups. The explainable AI and transparent computational governance will become essential requirements for future AI-guided antimicrobial nanomedicine. Patient privacy and biomedical data security are also important ethical concerns in precision antimicrobial therapy [158]. Personalized nano-antibiotic systems frequently rely on genomic data, microbiome analysis, clinical biomarkers, and multi-omics profiling to optimize treatment strategies. Such sensitive biological information requires strict protection against unauthorized access, misuse, or commercial exploitation. Data-sharing among hospitals, research institutions, AI developers, and pharmaceutical companies further complicates privacy management. Inadequate cybersecurity or poor data governance could undermine public trust and delay adoption of personalized nanomedicine technologies. The ethical management of patient-derived biological data must remain a foundational principle during future development of AI-integrated antimicrobial systems. Another important ethical issue involves equitable global access to advanced nano-antibiotic therapies. The development of multifunctional smart nanoplatforms often requires expensive materials, sophisticated manufacturing infrastructure, AI-based computational resources, and specialized clinical support systems. Such costs may restrict access primarily to technologically advanced healthcare systems while low-income regions continue facing severe antimicrobial resistance burdens with limited therapeutic resources 385. This disparity raises concerns regarding fairness and global healthcare inequality. In the future antimicrobial innovation should prioritize affordability and scalability alongside scientific sophistication to ensure that emerging technologies remain accessible to populations most affected by resistant infectious diseases. Environmental ethics additionally represent an emerging area of concern in antimicrobial nanotechnology [159]. Nanoparticles released during manufacturing, clinical use, or waste disposal may enter aquatic ecosystems, soil environments, and microbial communities where they could potentially disrupt ecological balance and contribute to environmental nanotoxicity. Metallic nanoparticles with strong antimicrobial activity may unintentionally affect beneficial environmental microorganisms involved in nutrient cycling and ecological stability. Regulatory agencies are therefore increasingly emphasizing life-cycle analysis and environmental risk assessment during nanomaterial evaluation. The environmentally sustainable design principles will become increasingly important for future development of clinically and ecologically responsible antimicrobial nanoplatforms. Despite these challenges, considerable progress is being made toward improving regulatory and ethical governance in nanomedicine. International organizations and regulatory agencies are gradually developing more specialized guidelines for nanotechnology-based therapeutics [160]. AI-assisted quality control, standardized nanomaterial databases, explainable machine learning frameworks, and advanced biosafety modeling may further improve regulatory reliability in the future. One of the major challenges in nanomedicine research is the variation in characterization methods used across different laboratories. Differences in synthesis procedures, analytical techniques, and biological testing protocols can produce inconsistent results, making it difficult to compare studies and evaluate nanomaterial performance reliably. Establishing standardized characterization guidelines, common reporting standards, and validated reference materials would help improve data quality and reproducibility. In addition, collaborative inter laboratory studies and greater coordination between researchers, industry, and regulatory agencies could support the development of internationally accepted evaluation frameworks for smart nano-antibiotic systems. The successful clinical translation of smart nano-antibiotics will ultimately require balanced integration of scientific innovation, ethical responsibility, transparent regulation, and global healthcare accessibility.

**Figure 6:**
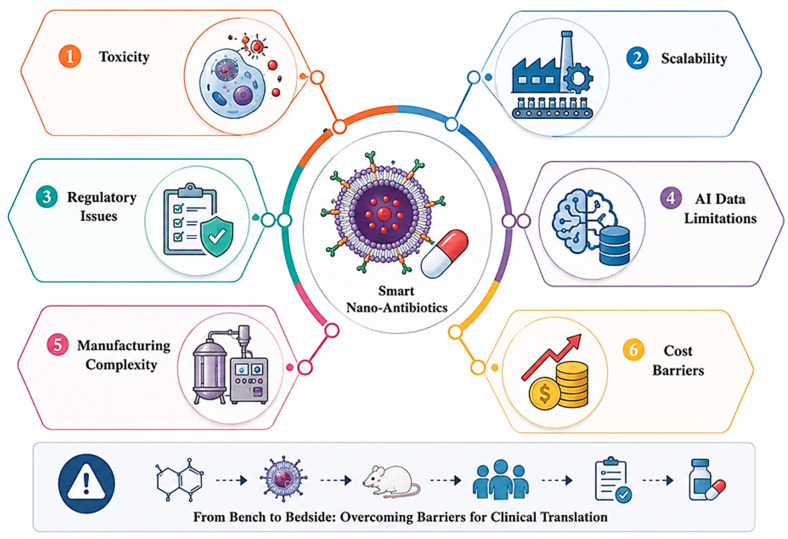
Schematic representation of challenges and translational barriers in smart Nano-Antibiotic development.

Figure 6 summarizes the major challenges limiting the clinical translation of smart nano-antibiotic systems. The schematic highlights important barriers including toxicity, manufacturing complexity, scalability limitations, regulatory uncertainty, AI data issues, and high production costs. It also emphasizes the need for standardized biosafety evaluation and interdisciplinary collaboration to support the successful development of clinically applicable antimicrobial nanomedicine.

### 7.4. Data Limitations in AI Models

Artificial intelligence has demonstrated enormous potential in antimicrobial nanomedicine; however, the effectiveness and reliability of AI-driven nano-antibiotic systems are fundamentally dependent on the quality, diversity, and scale of available datasets. Machine learning algorithms require large volumes of structured and biologically relevant data to generate accurate predictions regarding nanoparticle design, toxicity behavior, drug release kinetics, targeting efficiency, and therapeutic outcomes [161]. Unfortunately, nanomedicine datasets are often fragmented, inconsistent, heterogeneous, and experimentally incomplete. Such limitations significantly reduce model reliability and restrict the practical implementation of AI-guided antimicrobial nanotechnology. The data quality currently represents one of the most critical bottlenecks limiting the advancement of precision nano-antibiotic systems. One of the major challenges involves the lack of standardized experimental protocols across nanomedicine research studies. Nanoparticle synthesis methods, characterization techniques, biological assays, dosing conditions, and analytical procedures frequently vary among laboratories. As a result, datasets generated from different studies are often difficult to compare or integrate into unified machine learning frameworks [162]. Variations in particle size measurement, zeta potential analysis, toxicity evaluation, and antimicrobial testing can produce inconsistent outcomes even for apparently similar nanomaterials. This experimental heterogeneity reduces model generalizability and may introduce substantial prediction errors. Data inconsistency may conceptually be represented as:

*Prediction Accuracy* ∝ *Data Quality*

In the future progress in AI-guided nanomedicine will depend heavily on internationally standardized data generation and reporting systems. Another important limitation is the relatively small size of many nanomedicine datasets. Advanced machine learning and deep learning algorithms typically require very large datasets to achieve robust predictive performance. However, antimicrobial nanotechnology research is often expensive, technically complex, and experimentally time-consuming, limiting the availability of large-scale high-quality biological data. Many published studies involve only small experimental sample sizes or specific laboratory conditions that fail to capture broader biological variability [163]. Consequently, AI models trained on limited datasets may become over fitted, meaning they perform well under training conditions but fail to generalize accurately in real-world clinical environments. The insufficient dataset diversity is especially problematic because infectious diseases and patient responses are heterogeneous. Biological complexity further complicates AI model development in antimicrobial nanomedicine. Nano-bio interactions involve numerous dynamic processes including protein corona formation, immune response modulation, intracellular trafficking, microbial adaptation, metabolic signaling, and environmental responsiveness. These interactions vary according to patient physiology, pathogen species, infection stage, and tissue microenvironment. Capturing such multidimensional biological behavior within simplified computational models remains extremely difficult. Many AI systems rely primarily on physicochemical nanoparticle parameters while underrepresenting complex biological factors that strongly influence therapeutic outcomes. The future predictive models must incorporate more biologically comprehensive datasets including omics information, immune profiling, microbiome dynamics, and real-time biosensor data. Data imbalance and algorithmic bias are additional major concerns. Certain nanomaterials, pathogens, or experimental conditions are heavily represented in published literature, whereas rare diseases, emerging pathogens, or underrepresented populations may contribute very limited data [164]. Such imbalance can bias machine learning predictions toward dominant datasets while reducing accuracy for less represented clinical scenarios. AI models trained primarily on data from specific geographical regions or patient populations may additionally exhibit reduced predictive reliability in diverse global healthcare settings. The addressing algorithmic bias is critically important because antimicrobial resistance is a global challenge affecting diverse patient populations and healthcare infrastructures. Interpretability limitations also reduce clinical trust in AI-generated predictions. Many advanced deep learning systems function as complex nonlinear architectures whose internal decision-making processes remain difficult to understand [165]. Although such models may achieve strong predictive performance, clinicians and regulatory authorities often require mechanistic explanation and biological rationale before accepting therapeutic recommendations. Lack of explainability may therefore limit regulatory approval and clinical adoption of AI-guided nano-antibiotic systems. Explainable AI approaches capable of identifying influential biological variables and mechanistic relationships are increasingly being explored to improve transparency and scientific confidence. The interpretability will become an essential requirement for future clinical AI systems in precision antimicrobial therapy. Another important limitation involves restricted access to high-quality biomedical data due to privacy regulations, intellectual property concerns, and institutional data fragmentation [166]. Clinical datasets involving genomics, microbiome analysis, patient biomarkers, and treatment outcomes are often distributed across separate institutions with limited interoperability. Strict privacy regulations are essential for protecting patient information but may also complicate large-scale collaborative AI development. Federated learning and secure multi-institutional data-sharing frameworks are increasingly proposed as potential solutions for preserving privacy while enabling collaborative model training. The balancing data accessibility with ethical privacy protection will remain one of the most important challenges in future AI-integrated nanomedicine research. Despite these limitations, significant progress is being made toward improving AI data infrastructure in antimicrobial nanotechnology. Public nanomedicine databases, automated high-throughput screening systems, real-time biosensing technologies, and AI-assisted data harmonization approaches are steadily improving dataset quality and availability. Integration of multi-omics analysis, molecular simulations, and clinical monitoring systems may further enhance predictive reliability in the future. Improving model transparency and reducing algorithmic bias will be essential for gaining regulatory approval and clinical acceptance of AI-guided nano-antibiotic systems. Ultimate, overcoming current data limitations will require global interdisciplinary collaboration, standardized experimental frameworks, transparent AI methodologies, and large-scale clinically relevant datasets capable of supporting truly reliable precision nano-antibiotic development. Table 6 outlines the major scientific and translational challenges limiting the clinical development of smart nano-antibiotic systems. The table highlights important barriers including toxicity concerns, manufacturing complexity, scalability issues, regulatory uncertainty, and AI-related data limitations that affect large-scale medical implementation. Overcoming these challenges will be essential for the successful translation of advanced antimicrobial nanomedicine into routine clinical practice.

## 8. Clinical Translation and Current Applications

### 8.1. Preclinical Studies

Preclinical studies play a fundamental role in the development of smart nano-antibiotics because they provide the first comprehensive evaluation of antimicrobial efficacy, pharmacokinetics, biosafety, targeting capability, and therapeutic feasibility before human clinical application. The transition from laboratory-scale nanomaterial synthesis to clinically relevant antimicrobial systems requires extensive in vitro and in vivo investigation to determine whether advanced nanoplatforms can effectively function within complex biological environments [167]. Preclinical evaluation is especially important in antimicrobial nanomedicine because nanoscale therapeutic systems often exhibit dynamic interactions with immune systems, proteins, biofilms, and tissues that cannot be predicted solely from physicochemical characterization. The preclinical research serves not only as a safety checkpoint but also as a critical optimization stage where therapeutic design, targeting efficiency, and biological responsiveness are refined before clinical translation. In vitro studies are generally the first step in evaluating antimicrobial nanoplatform performance [168]. These experiments commonly assess bacterial growth inhibition, minimum inhibitory concentration (MIC), minimum bactericidal concentration (MBC), biofilm penetration, intracellular bacterial killing, cytotoxicity, and controlled drug release behavior. Smart nano-antibiotics have demonstrated significant in vitro efficacy against multidrug-resistant pathogens including methicillin-resistant *Staphylococcus aureus* (MRSA), *Pseudomonas aeruginosa*, *Escherichia coli*, *Klebsiella pneumoniae*, and vancomycin-resistant enterococci [169]. Metallic nanoparticles such as silver and zinc oxide systems frequently exhibit broad-spectrum antimicrobial activity through ROS generation and membrane disruption, while polymeric and lipid-based carriers improve intracellular delivery and sustained release profiles. Antimicrobial performance is often quantitatively evaluated using equations such as:

*MIC* = *Minimum Drug Concentration*/*Visible Bacterial Inhibition*

The advanced in vitro models including biofilm systems, organoids, and microfluidic infection platforms are becoming increasingly important because traditional planktonic bacterial assays often fail to accurately represent real clinical infection conditions. Biofilm-associated infection models have received particularly strong attention during preclinical development because biofilms are resistant to conventional antibiotics. Numerous nano-antibiotic systems have demonstrated enhanced penetration into extracellular polymeric matrices and improved eradication of mature biofilms compared with free antibiotics. Stimuli-responsive nanoplatforms capable of pH-triggered, enzyme-triggered, or photothermal activation have shown especially promising results in disrupting resistant microbial communities [23]. Multifunctional systems integrating ROS generation, quorum sensing inhibition, and sustained drug release have further improved antibiofilm efficacy in experimental settings. A successful preclinical biofilm eradication studies are important because chronic biofilm-associated infections remain one of the greatest unmet clinical challenges in antimicrobial therapy. In vivo animal studies provide additional insight into biodistribution, pharmacokinetics, immune interaction, therapeutic efficacy, and long-term safety. Mouse, rat, rabbit, and zebrafish infection models are commonly used to evaluate systemic infections, wound healing, implant-associated infections, pneumonia, osteomyelitis, and sepsis. Preclinical in vivo studies have demonstrated that smart nanoplatforms can significantly improve infection-site accumulation, prolong antimicrobial retention, reduce systemic toxicity, and enhance survival outcomes compared with conventional antibiotic therapies [170]. Targeted and stimuli-responsive systems often exhibit improved therapeutic specificity by releasing antimicrobial agents selectively within infected tissues while minimizing exposure to healthy organs. The physiologically relevant animal models are essential for understanding the dynamic biological behavior of nano-antibiotics under clinically realistic conditions. Pharmacokinetic and biodistribution studies are also critical during preclinical development. Nanoparticle size, surface charge, hydrophobicity, and surface functionalization strongly influence circulation time, tissue accumulation, organ clearance, and immune recognition. Imaging techniques such as fluorescence imaging, magnetic resonance imaging, positron emission tomography, and photoacoustic imaging are increasingly used to monitor nanoparticle localization and therapeutic response in real time [171]. AI-assisted modeling further improves prediction of biodistribution behavior and optimization of formulation parameters. The understanding in vivo nanoparticle fate is one of the most important requirements for achieving clinically reliable precision antimicrobial therapy. Despite encouraging results, preclinical studies also reveal substantial translational challenges. Many nanoplatforms demonstrating strong laboratory performance fail to maintain similar effectiveness under complex physiological conditions. Protein corona formation, immune clearance, tissue heterogeneity, and biological variability frequently alter nanoparticle behavior in vivo. In addition, differences between animal models and human physiology limit direct clinical extrapolation [172]. Long-term toxicity and chronic accumulation remain insufficiently understood for several nanomaterial systems. The future preclinical research must increasingly focus on clinically relevant infection models, standardized evaluation protocols, and integrated AI-driven predictive systems capable of improving translational reliability between laboratory findings and human therapeutic outcomes. Table 7 highlights the rapid advancement of smart nano-antibiotic systems from experimental research toward translational antimicrobial applications. The summarized studies demonstrate improved therapeutic precision, enhanced antimicrobial efficacy, and better infection-targeted delivery against resistant pathogens. Collectively, these findings support the growing clinical potential of intelligent nanomedicine for next-generation antimicrobial therapy.

### 8.2. Clinical Trial Landscape

The clinical translation of smart nano-antibiotics is gradually progressing from experimental research toward human therapeutic evaluation, although the clinical landscape remains relatively limited compared with other areas of nanomedicine. Numerous antimicrobial nanoplatforms have demonstrated remarkable preclinical performance, yet only a small proportion have advanced into clinical trials due to regulatory complexity, manufacturing challenges, biosafety concerns, and translational uncertainty [173]. Nevertheless, increasing global antimicrobial resistance and the declining effectiveness of conventional antibiotics are accelerating interest in clinically applicable nanotechnology-based antimicrobial therapies. The current clinical trial landscape represents an important transitional stage where experimental nanomedicine concepts are beginning to move toward real-world infectious disease management. Most clinically investigated antimicrobial nanoplatforms currently involve lipid-based nanocarriers, polymeric systems, metallic nanoparticles, and antimicrobial coating technologies. Liposomal antibiotic formulations are among the most advanced clinically because liposomes exhibit relatively favorable biocompatibility and established pharmaceutical manufacturing pathways. Several liposomal formulations have demonstrated improved drug stability, prolonged circulation, enhanced intracellular delivery, and reduced systemic toxicity compared with conventional antibiotics [174]. Liposomal amikacin inhalation systems for pulmonary infections and liposomal amphotericin B for fungal infections are important examples demonstrating Therapeutic potential of nanotechnology-assisted antimicrobial delivery. The clinically successful liposomal systems provide valuable translational frameworks for future smart nano-antibiotic development. Metal-based nanoparticles are also increasingly entering early-stage clinical investigation, particularly in wound healing, topical infection management, and implant-associated antimicrobial applications [175]. Silver nanoparticle-coated wound dressings, catheter coatings, orthopedic implants, and antimicrobial surface materials have demonstrated promising clinical utility due to their broad-spectrum antimicrobial properties and biofilm suppression capability. Several studies have reported improved wound healing outcomes and reduced infection rates using silver-containing nanomaterials. However, systemic administration of metallic nanoparticles remains more limited because long-term biosafety, biodistribution, and accumulation concerns require further evaluation. A topical and localized applications may currently represent the most practical clinical entry point for metallic nano-antibiotic technologies. Stimuli-responsive and AI-assisted nano-antibiotic systems are still largely in early developmental or preclinical stages, although interest in these technologies is rapidly increasing [95]. Personalized antimicrobial nanomedicine involving biosensing integration, real-time therapeutic monitoring, and adaptive drug release remains scientifically promising but clinically complex. Regulatory approval pathways for such multifunctional systems are considerably more challenging because therapeutic performance depends not only on the nanomaterial itself but also on integrated computational algorithms, environmental responsiveness, and biological feedback mechanisms [176]. In the future clinical trials will increasingly evaluate multifunctional smart nanoplatforms capable of combining diagnosis, targeting, monitoring, and therapy within unified precision treatment systems. Clinical trials involving antimicrobial nanotechnology frequently focus on specific high-risk infection categories where conventional therapies are inadequate. Chronic wound infections, diabetic ulcers, implant-associated biofilms, pulmonary infections, multidrug-resistant bacterial diseases, and catheter-associated infections are among the most actively investigated targets. Nano-antibiotic systems are particularly attractive in these conditions because localized delivery and sustained antimicrobial release may significantly improve therapeutic outcomes while reducing systemic toxicity [177]. Combination systems integrating antibiotics with photothermal therapy, ROS generation, or immune modulation are additionally being explored for resistant and recurrent infections. The clinical application will likely expand first in areas where localized therapy and biofilm disruption offer clear advantages over conventional antimicrobial treatment approaches. Despite growing progress, several major limitations continue to slow clinical translation. Regulatory uncertainty remains one of the largest barriers because standardized frameworks for evaluating multifunctional nanoplatforms are still evolving. Manufacturing reproducibility, large-scale stability, sterilization, and quality control also remain difficult for many complex nano-antibiotic systems. Furthermore, clinical trial costs for advanced nanomedicine technologies are often exceptionally high, limiting rapid commercial development. Long-term biosafety assessment is particularly important because nanoparticles may exhibit prolonged tissue retention or unpredictable immune interactions. The stronger collaboration among clinicians, nanotechnologists, regulatory agencies, pharmaceutical industries, and computational scientists will be essential for accelerating future clinical adoption of smart antimicrobial nanomedicine. Another important consideration is the growing role of AI in clinical trial design and patient stratification [178]. Machine learning systems are increasingly being explored to predict therapeutic response, optimize dosing strategies, identify suitable patient populations, and monitor treatment outcomes in nanomedicine-based clinical studies. AI-assisted biomarker analysis and multi-omics integration may further improve precision therapeutic evaluation in future antimicrobial trials. The integration of AI with clinical nanomedicine could substantially improve trial efficiency and support the development of more personalized antimicrobial treatment strategies. The clinical trial landscape for smart nano-antibiotics remains in an evolving but promising stage. Although relatively few systems have achieved advanced clinical approval, the rapid expansion of antimicrobial resistance continues to create urgent demand for innovative therapeutic solutions. In future clinical progress will likely depend on balancing multifunctional therapeutic sophistication with regulatory simplicity, scalable manufacturing, long-term safety assurance, and economically feasible healthcare implementation.

### 8.3. Industrial and Commercial Aspects

The industrial and commercial development of smart nano-antibiotics is becoming increasingly important as global antimicrobial resistance creates urgent demand for innovative therapeutic technologies. Although antimicrobial nanomedicine remains scientifically advanced and commercially emerging, substantial investment from pharmaceutical industries, biotechnology companies, nanotechnology startups, and healthcare sectors is accelerating the transition of nano-antibiotic systems from laboratory research toward industrial application [179]. Commercial interest is particularly strong in areas where conventional antibiotics demonstrate limited effectiveness, including multidrug-resistant infections, chronic wounds, implant-associated biofilms, and hospital-acquired infections. The successful commercialization will ultimately determine whether smart antimicrobial nanotechnologies can produce meaningful global healthcare impact beyond academic research environments. One of the most commercially active sectors involves antimicrobial coatings and surface-engineered medical products. Silver nanoparticle-coated wound dressings, surgical instruments, orthopedic implants, dental materials, catheters, and hospital surface coatings are already being marketed in several healthcare systems due to their strong antimicrobial and antibiofilm properties [180]. Such technologies reduce microbial colonization and lower the risk of healthcare-associated infections, particularly in intensive care environments and implant-related procedures. Compared with systemic nano-antibiotics, antimicrobial coatings generally face fewer regulatory complexities because they function primarily as localized surface-protection technologies rather than internally circulating therapeutic agents. The surface-based nanotechnology products represent one of the earliest commercially sustainable pathways for antimicrobial nanomedicine. Pharmaceutical companies are also increasingly exploring lipid-based and polymeric nanocarrier systems for improved antibiotic delivery. Liposomal antimicrobial formulations have demonstrated commercial viability because they improve pharmacokinetics, reduce toxicity, and extend therapeutic lifetime for several established antimicrobial agents. Nanocarrier-assisted reformulation of existing antibiotics is particularly attractive industrially because it may extend the clinical usefulness of previously approved drugs while reducing the cost and risk associated with entirely new antibiotic discovery [181]. Controlled-release injectable systems, inhalable nanoformulations, and localized antimicrobial hydrogels are also gaining industrial attention for chronic and resistant infections. The repurposing conventional antibiotics through nanotechnology-enhanced delivery may become one of the most economically practical strategies for addressing antimicrobial resistance. The integration of artificial intelligence into nano-antibiotic development is additionally reshaping industrial innovation models. AI-assisted material screening, predictive toxicity analysis, drug release optimization, and manufacturing process control significantly reduce development time and experimental cost. Pharmaceutical industries increasingly utilize machine learning algorithms to identify promising nanoparticle formulations and optimize large-scale production parameters. Automated high-throughput synthesis and robotic screening systems are further accelerating industrial research efficiency. The AI-driven industrial nanomedicine platforms may substantially shorten development cycles and improve the commercial feasibility of precision antimicrobial technologies. Manufacturing scalability and production economics remain major industrial challenges. Many multifunctional nanoplatforms require sophisticated fabrication procedures involving precise nanoscale engineering, ligand conjugation, purification, and quality control [182]. Such complexity significantly increases manufacturing cost and limits large-scale reproducibility. Industrial production additionally requires strict control over particle size distribution, encapsulation efficiency, surface chemistry, sterility, and long-term stability to satisfy regulatory standards. Continuous-flow synthesis systems, microfluidic manufacturing technologies, and AI-assisted process optimization are increasingly explored to improve industrial scalability and batch consistency. The manufacturing simplification without substantial loss of therapeutic functionality will likely become one of the most important priorities for future commercial nanomedicine development. Intellectual property and patent protection are also important in the commercial landscape of smart nano-antibiotics. Companies developing novel nanomaterials, targeting ligands, responsive systems, AI-integrated platforms, or multifunctional antimicrobial formulations actively pursue patent protection to secure competitive market advantages. However, overlapping patents and rapidly evolving technological complexity may create legal and licensing challenges [183]. Collaborative partnerships among academic institutions, biotechnology startups, pharmaceutical companies, and healthcare organizations are therefore becoming increasingly common to accelerate commercialization and share technological expertise. The interdisciplinary industrial collaboration will be essential for translating advanced antimicrobial nanotechnologies into clinically accessible products. Economic accessibility remains another critical commercial consideration. Many advanced smart nanoplatforms involve expensive raw materials, specialized manufacturing infrastructure, and sophisticated analytical technologies. Such costs may limit availability in low-resource healthcare systems despite the severe burden of antimicrobial resistance in developing regions. Cost-effectiveness analysis is therefore becoming increasingly important during industrial planning and clinical adoption evaluation. The future nano-antibiotic commercialization strategies must prioritize affordability, scalability, and global accessibility alongside scientific sophistication. Environmental sustainability is also emerging as an important industrial issue in antimicrobial nanotechnology. Large-scale production and disposal of nanomaterials may contribute to environmental nanoparticle accumulation and ecological toxicity if not properly managed. Regulatory agencies and industries are therefore increasingly focusing on biodegradable materials, green synthesis methods, and environmentally sustainable manufacturing practices [184]. The environmentally responsible commercialization will become increasingly important as antimicrobial nanotechnology expands into broader healthcare markets. The industrial and commercial future of smart nano-antibiotics remains promising but still faces substantial scientific, economic, and regulatory challenges. Continued advances in AI-assisted manufacturing, scalable production technologies, precision nanomaterial engineering, and collaborative industrial partnerships are expected to accelerate future commercialization. The long-term success of antimicrobial nanomedicine will ultimately depend on achieving a practical balance among therapeutic innovation, manufacturing feasibility, regulatory approval, economic accessibility, and global healthcare impact. Although significant progress has been achieved in smart nano-antibiotic research, it is important to recognize that most reported systems remain at the in vitro or preclinical stage of development. Many AI-guided design strategies have demonstrated predictive capability for nanomaterial optimization, toxicity assessment, and drug delivery performance; however, large-scale clinical validation remains limited. Similarly, multifunctional stimuli-responsive nanoplatforms have shown encouraging results in laboratory and animal studies, but their long-term safety, manufacturing reproducibility, regulatory acceptance, and cost-effectiveness have not yet been fully established. Therefore, several concepts discussed in this review, including autonomous AI-guided therapeutic systems and closed-loop antimicrobial nanomedicine platforms, should currently be regarded as emerging future directions rather than clinically validated technologies.

## 9. Future Perspectives

### 9.1. Self-Adaptive and Intelligent Nanoplatforms

Self-adaptive and intelligent nanoplatforms are expected to represent the next major evolution in antimicrobial nanomedicine because they introduce dynamic therapeutic behavior capable of responding autonomously to changing biological environments. Conventional antimicrobial therapies generally rely on fixed dosing schedules and passive drug release systems that cannot actively adjust according to infection progression, microbial resistance, or patient-specific physiological conditions [185]. In contrast, intelligent nano-antibiotics are being designed to sense environmental stimuli, analyze biological signals, and modify therapeutic activity in real time through integrated responsive materials, biosensing systems, and artificial intelligence-guided decision mechanisms. These adaptive nanoplatforms may fundamentally transform antimicrobial therapy from static drug administration into responsive and continuously regulated precision treatment. One of the most important characteristics of self-adaptive nanoplatforms is their ability to respond selectively to pathological microenvironments associated with infection [186]. Infectious tissues frequently exhibit distinct biochemical features such as acidic pH, elevated reactive oxygen species (ROS), hypoxia, abnormal enzyme expression, inflammatory cytokines, and altered metabolic activity. Smart nanomaterials can be engineered to recognize these signals and activate therapeutic functions only within infected regions. Stimuli-responsive behavior may conceptually be represented as:

*Stimulus* + *Smart Nano-platform* → *Adaptive Therapeutic Response*

For example, pH-sensitive nanocarriers may remain inactive during systemic circulation but rapidly release antibiotics within acidic biofilm environments. Similarly, ROS-responsive systems can increase antimicrobial release in inflamed tissues where oxidative stress is elevated. The environmental responsiveness significantly improves therapeutic precision while minimizing unnecessary exposure to healthy tissues. Artificial intelligence is expected to play a central role in future intelligent nanoplatforms because adaptive therapeutic regulation requires continuous analysis of complex biological information [187]. Machine learning systems can process multidimensional datasets involving microbial behavior, patient biomarkers, drug response patterns, immune signaling, and biosensor outputs to optimize nanoparticle performance dynamically. AI-guided systems may eventually predict infection progression and adjust antimicrobial release kinetics before clinical deterioration occurs. Such predictive therapeutic regulation could substantially improve treatment outcomes in chronic infections and multidrug-resistant diseases. AI-assisted adaptive nanomedicine may become one of the most important innovations in future precision healthcare because it enables therapeutic systems to evolve in response to real-time biological conditions [46]. Another promising direction involves the development of autonomous closed-loop therapeutic systems. These systems combine biosensors, responsive nanocarriers, and computational algorithms into integrated platforms capable of continuously monitoring infection biomarkers and regulating therapy without external intervention. Biosensors may detect bacterial toxins, inflammatory cytokines, pH changes, oxygen concentration, or metabolic signals associated with infection severity. Detected signals can then trigger adaptive drug release, photo-thermal activation, ROS generation, or immune modulation through intelligent nanomaterials. The closed-loop therapeutic platforms could dramatically improve treatment precision while reducing delayed clinical response associated with conventional infection management strategies. Self-adaptive nanoplatforms are also expected to improve management of antimicrobial resistance. Resistant bacteria continuously evolve under therapeutic pressure through genetic mutation, efflux pump activation, metabolic adaptation, and biofilm formation. Intelligent nanocarriers capable of dynamically altering drug combinations, release profiles, or targeting behavior may reduce bacterial adaptation efficiency and prolong therapeutic effectiveness [58]. Multifunctional systems integrating antibiotics with antimicrobial peptides, quorum sensing inhibitors, CRISPR-based gene suppression, or photothermal agents may provide flexible resistance-management strategies. The adaptive therapeutic flexibility may become increasingly important as microbial resistance mechanisms continue to evolve globally. Biomimetic and biohybrid nanoplatforms are additionally emerging as advanced intelligent therapeutic systems. These platforms integrate synthetic nanomaterials with biological membranes, engineered cells, or naturally derived biomolecules to achieve enhanced responsiveness and physiological compatibility. Cell membrane-coated nanoparticles can inherit inflammatory targeting, immune evasion, and toxin-neutralizing properties from source cells. Engineered bacterial vesicles and synthetic biological circuits are also being explored for autonomous infection sensing and localized antimicrobial production. In the future intelligent antimicrobial systems may increasingly combine nanotechnology with synthetic biology to achieve sophisticated therapeutic adaptability. Despite enormous promise, several major challenges remain before intelligent nanoplatforms can achieve widespread clinical implementation. Designing stable systems capable of accurate environmental sensing and controlled therapeutic adaptation under variable physiological conditions remains technically demanding [188]. Excessive complexity may also increase manufacturing difficulty, regulatory uncertainty, and production cost. Furthermore, AI-guided therapeutic systems require large volumes of reliable biological data and transparent computational decision-making frameworks to ensure clinical safety and trust. The future success will depend on interdisciplinary collaboration among nanotechnologists, microbiologists, clinicians, computational scientists, and regulatory authorities to develop clinically reliable adaptive antimicrobial systems. Ethical and cybersecurity considerations will also become increasingly important as intelligent therapeutic platforms become more digitally integrated [189]. AI-guided systems relying on real-time patient monitoring and cloud-based computational analysis may create concerns regarding privacy, algorithmic transparency, and potential cybersecurity vulnerabilities. Maintaining secure and ethically responsible therapeutic automation will therefore be essential for future clinical adoption. The intelligent antimicrobial nanomedicine must evolve not only as a technological innovation but also as a responsibly governed healthcare system capable of balancing automation, safety, privacy, and therapeutic effectiveness. Self-adaptive and intelligent nanoplatforms represent one of the most exciting future directions in antimicrobial therapy. Continued progress in responsive biomaterials, AI-driven predictive modeling, biosensing integration, and synthetic biology is expected to accelerate the development of autonomous precision antimicrobial systems. In the future antimicrobial nanomedicine may ultimately transition from passive drug delivery platforms toward intelligent therapeutic ecosystems capable of continuously sensing, analyzing, and responding to infection dynamics in real time.

### 9.2. Integration with Biosensors and Real-Time Monitoring

The integration of biosensors and real-time monitoring technologies with smart nano-antibiotics is emerging as a transformative strategy in precision antimicrobial medicine because it enables continuous observation of infection dynamics, therapeutic response, and patient physiological status during treatment. Conventional antimicrobial therapy often relies on delayed laboratory testing and periodic clinical evaluation, which may fail to detect rapid infection progression or therapeutic failure in real time [190]. Smart biosensor-integrated nanoplatforms provide the possibility of continuously monitoring biological signals associated with infection while simultaneously adjusting therapeutic activity through responsive nanomaterials and AI-guided decision systems. The combination of nanotechnology, biosensing, and real-time analytics may fundamentally redefine future infectious disease management by creating interactive and adaptive therapeutic systems. Biosensors are analytical devices capable of detecting specific biological or chemical signals and converting them into measurable outputs [191]. In antimicrobial nanomedicine, biosensors are increasingly designed to monitor biomarkers such as bacterial toxins, inflammatory cytokines, pH variation, ROS levels, oxygen concentration, enzyme activity, metabolic products, and microbial genetic material. Nanomaterials significantly improve biosensor sensitivity because of their exceptional electrical, optical, magnetic, and catalytic properties. Signal detection behavior may conceptually be represented as:

*Biological Signal* + *Biosensor* → *Measurable Response*

Gold nanoparticles, graphene, quantum dots, carbon nanotubes, magnetic nanoparticles, and conductive polymers are commonly integrated into biosensor systems to amplify detection sensitivity and improve signal transduction efficiency. The nanoscale biosensors may eventually enable ultra-early infection detection before clinical symptoms become severe. Real-time monitoring capability is especially valuable in managing severe infections, chronic wounds, sepsis, implant-associated biofilms, and multidrug-resistant bacterial diseases [192]. Infection microenvironments continuously change during disease progression, often involving dynamic alterations in pH, oxygen availability, inflammatory signaling, and microbial load. Smart biosensor systems integrated with nano-antibiotic platforms can continuously evaluate these changes and provide immediate therapeutic feedback. For example, pH-sensitive biosensors embedded within wound dressings may detect localized infection progression and trigger controlled antibiotic release from associated nanocarriers. Similarly, implantable biosensors may identify early biofilm formation on medical devices before extensive bacterial colonization occurs. The early real-time detection may significantly reduce therapeutic delay and improve patient survival outcomes in severe infections. The integration of biosensors with artificial intelligence further enhances the predictive capability of antimicrobial nanomedicine [193]. AI algorithms can analyze continuous biosensor-generated data streams to identify hidden biological patterns, predict infection progression, optimize drug release timing, and personalize therapeutic strategies according to patient-specific responses. Machine learning systems may additionally distinguish between bacterial and sterile inflammatory conditions using multidimensional biomarker analysis. Such predictive monitoring enables proactive therapeutic adjustment rather than reactive intervention after clinical deterioration. The AI-assisted biosensing may become central to future precision infection management because infectious diseases are dynamic and biologically heterogeneous. Wearable and implantable biosensor technologies are receiving growing attention in advanced antimicrobial healthcare systems. Smart wound dressings containing nanosensors, flexible electronic materials, and responsive antimicrobial nanocarriers are being developed for continuous monitoring of diabetic ulcers, burn injuries, and chronic wound infections [194]. These systems can evaluate moisture levels, temperature, pH, and bacterial biomarkers while simultaneously delivering localized therapy. Implantable biosensors integrated with orthopedic devices, catheters, or tissue scaffolds are also being investigated for long-term infection surveillance. The wearable and implantable technologies may eventually support decentralized infection management and reduce dependence on repeated hospital-based diagnostic testing. Another important future direction involves theranostic nanoplatforms that combine diagnostic and therapeutic functionality within single integrated systems. Such multifunctional nanoplatforms can simultaneously detect infection biomarkers, visualize infection sites through imaging modalities, and deliver antimicrobial therapy in response to detected signals [195]. Magnetic nanoparticles, fluorescent probes, and photoresponsive materials are frequently integrated into theranostic systems for combined imaging and treatment applications. Real-time therapeutic monitoring may additionally help optimize dosing schedules and reduce unnecessary antibiotic exposure. Theranostic nanomedicine could substantially improve clinical precision by integrating diagnosis and treatment into unified responsive platforms. Despite their enormous potential, biosensor-integrated antimicrobial systems still face several important challenges. Maintaining long-term sensor stability and accuracy within complex physiological environments remains technically difficult. Biofouling, signal interference, sensor degradation, and limited power supply may reduce monitoring reliability during prolonged clinical use.

**Figure 7:**
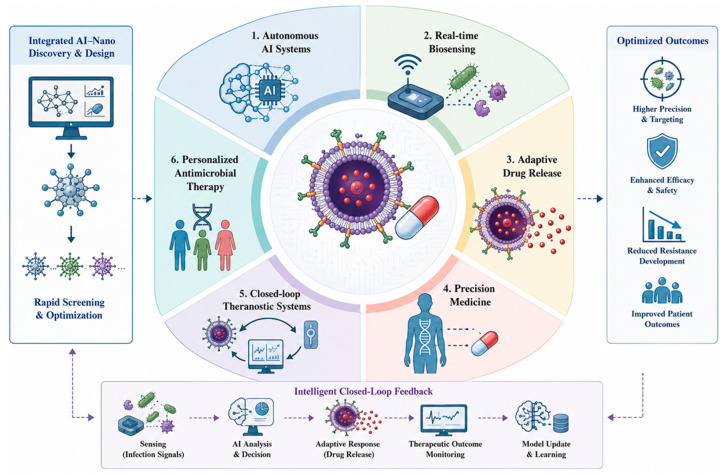
Diagrammatic schematic presentation of future perspectives of AI-guided intelligent nano-antibiotic platforms.

Data management and cybersecurity are additional concerns because continuous biosensor monitoring generates large volumes of sensitive patient information. Ensuring secure data transmission and protecting patient privacy will therefore become increasingly important as digital healthcare integration expands. The robust cybersecurity frameworks and ethical data governance will be essential for maintaining public trust in future intelligent antimicrobial systems. Manufacturing complexity and regulatory approval also remain significant obstacles. Integrating biosensors, nanocarriers, electronic components, and AI-driven computational systems into clinically reliable platforms requires sophisticated interdisciplinary engineering [196]. Regulatory evaluation becomes more complicated because such systems involve combined therapeutic, diagnostic, and digital healthcare functions. The future advancement will depend on collaborative efforts among nanotechnologists, biomedical engineers, clinicians, data scientists, and regulatory agencies to establish standardized and clinically practical development pathways. One promising strategy for personalized antimicrobial therapy involves integrating real-time biosensing platforms with AI-driven decision-making systems. Biosensors capable of continuously monitoring infection-related biomarkers, inflammatory signals, bacterial load, or treatment responses can provide dynamic datasets for machine learning algorithms. By analyzing these data in real time, AI models may predict disease progression, evaluate therapeutic effectiveness, and recommend individualized adjustments in drug dosage, release rate, or treatment duration. The development of closed-loop therapeutic systems that combine biosensing, data analytics, and responsive nanocarriers could enable adaptive antimicrobial interventions tailored to the evolving condition of individual patients. Such an approach may improve therapeutic outcomes while minimizing overtreatment and resistance development. Ultimate, the integration of biosensors and real-time monitoring technologies with smart nano-antibiotics represents one of the most promising future directions in precision antimicrobial medicine. Continued progress in nanosensors, flexible electronics, AI-assisted analytics, and responsive biomaterials is expected to accelerate the development of intelligent therapeutic systems capable of continuous infection monitoring and adaptive treatment control. The future antimicrobial healthcare may increasingly rely on interconnected theranostic platforms that integrate diagnosis, monitoring, prediction, and personalized therapy within unified real-time biomedical ecosystems. Figure 7 highlights the future direction of AI-guided smart nano-antibiotic systems for precision antimicrobial therapy. The schematic shows how artificial intelligence, biosensing technologies, and adaptive nanocarriers can work together for targeted and personalized infection treatment. It also demonstrates the potential of self-regulated drug delivery systems capable of real-time therapeutic monitoring and controlled antimicrobial response.

### 9.3. Autonomous AI-Driven Drug Development

Autonomous AI-driven drug development is emerging as one of the most transformative future directions in antimicrobial nanomedicine because it has the potential to fundamentally accelerate the discovery, optimization, and clinical translation of smart nano-antibiotics. Conventional antimicrobial drug development is often slow, expensive, and experimentally inefficient, frequently requiring more than a decade of research and enormous financial investment before achieving clinical approval [197]. The rapid global expansion of antimicrobial resistance (AMR) has exposed the limitations of traditional pharmaceutical discovery pipelines, particularly because resistant pathogens evolve much faster than new antibiotics can be developed. Artificial intelligence integrated with automated nanotechnology platforms now offers the possibility of creating accelerated and partially autonomous antimicrobial innovation systems capable of continuously designing, testing, and optimizing therapeutic candidates with minimal human intervention. The AI-driven autonomous development may become essential for sustaining future antimicrobial innovation in the face of rapidly evolving resistant pathogens. Machine learning algorithms are increasingly capable of analyzing massive multidimensional datasets involving nanoparticle composition, molecular structure, biological interaction, antimicrobial activity, pharmacokinetics, toxicity profiles, and clinical outcomes [198]. By recognizing hidden relationships within these complex datasets, AI systems can predict promising nano-antibiotic formulations before physical laboratory synthesis occurs. Predictive optimization may conceptually be represented as:

*Input Data* + *AI Model* → *Optimized Nano* − *Antibiotic Design*

Such predictive capability dramatically reduces experimental workload and accelerates identification of therapeutically effective nanoplatforms. AI-assisted molecular screening can simultaneously evaluate thousands of potential material combinations, drug-loading strategies, targeting ligands, and release mechanisms within very short computational timeframes. The predictive AI optimization may significantly shorten antimicrobial development cycles and reduce the economic burden associated with conventional trial-and-error experimentation. Autonomous robotic laboratories integrated with AI systems are also becoming increasingly important in advanced pharmaceutical research [199]. These systems combine automated synthesis platforms, high-throughput screening technologies, robotic experimentation, and machine learning algorithms into closed-loop research environments capable of continuously generating and evaluating new nano-antibiotic candidates. AI algorithms analyze experimental outcomes in real time and subsequently redesign future experiments to optimize antimicrobial performance, toxicity profiles, and stability characteristics. Such iterative self-learning systems may operate continuously with minimal manual intervention, thereby dramatically increasing research efficiency. The autonomous experimental platforms may eventually revolutionize antimicrobial research by transforming drug discovery into a continuously adaptive computational-engineering process. Generative artificial intelligence and deep learning models are additionally expanding the possibilities of antimicrobial nanomaterial design [200]. Advanced generative algorithms can create entirely novel nanoparticle architectures, responsive polymers, antimicrobial peptides, and multifunctional nanocarriers based on predefined therapeutic objectives. These AI systems may design materials with optimized physicochemical properties specifically tailored for biofilm penetration, intracellular delivery, stimuli responsiveness, or reduced toxicity. Computational molecular simulation and digital twin technologies further allow virtual evaluation of nano–bio interactions before laboratory validation. The generative AI may substantially expand the diversity of potential antimicrobial nanomaterials beyond what is achievable through conventional human-guided design approaches. Another important advantage of autonomous AI-driven development involves personalized therapeutic optimization. Future AI systems may integrate patient genomics, microbiome composition, immune profiles, metabolic status, and infection biomarkers to design individualized nano-antibiotic therapies. Real-time biosensor feedback and clinical monitoring data could further allow adaptive modification of therapeutic strategies during treatment [201]. Such precision-guided approaches may improve efficacy while minimizing toxicity and resistance development. The convergence of AI, biosensing, and nanotechnology could eventually enable personalized antimicrobial systems tailored dynamically for each individual patient. AI-driven autonomous platforms are also expected to improve predictive safety assessment and regulatory preparation. Toxicity prediction models trained on large biological datasets may identify high-risk nanomaterials early during development, thereby reducing unnecessary experimental cost and improving clinical safety. Machine learning systems may additionally optimize manufacturing scalability, stability conditions, and quality control procedures before industrial production begins [202]. Such integrated predictive capability could substantially improve translational efficiency from laboratory discovery to commercial implementation. The AI-assisted regulatory modeling and digital quality management may become increasingly important for accelerating clinical approval of complex multifunctional nanomedicines. Despite enormous promise, several major challenges remain before fully autonomous antimicrobial drug development becomes clinically reliable. AI systems remain heavily dependent on large, high-quality datasets that are often limited or experimentally inconsistent in nanomedicine research 493. Algorithmic bias, lack of interpretability, and poor generalization across biological systems may reduce predictive reliability. In addition, fully autonomous experimental systems require sophisticated infrastructure, extensive computational resources, and standardized data integration frameworks [203]. Ethical concerns additionally arise regarding intellectual property ownership, accountability for AI-generated decisions, and transparency of computational therapeutic design. The maintaining human scientific oversight will remain critically important even as AI systems become increasingly autonomous. Cybersecurity and digital infrastructure reliability also represent emerging concerns in AI-driven pharmaceutical systems. Autonomous research platforms relying on cloud-based data integration and automated decision-making may become vulnerable to data corruption, system malfunction, or unauthorized interference. Ensuring secure computational governance and transparent algorithmic validation will therefore be essential for maintaining scientific integrity and public trust. The future antimicrobial innovation systems must evolve with strong ethical, technical, and cybersecurity safeguards alongside increasing automation capability. Autonomous AI-driven drug development represents one of the most revolutionary future opportunities in smart antimicrobial nanomedicine. Continued advances in machine learning, robotics, computational biology, generative AI, and automated laboratory technologies are expected to dramatically accelerate the creation of next-generation nano-antibiotics. The future antimicrobial discovery may increasingly shift from slow sequential experimentation toward intelligent self-optimizing research ecosystems capable of rapidly responding to emerging resistant pathogens and evolving global healthcare needs.

### 9.4. Opportunities in Combating AMR

Antimicrobial resistance (AMR) is currently recognized as one of the greatest global public health threats because resistant pathogens continue to emerge faster than new therapeutic solutions become available. The rapid spread of multidrug-resistant bacteria, biofilm-associated infections, and antibiotic-tolerant microbial populations has significantly reduced the effectiveness of many conventional antimicrobial agents [9]. Smart nano-antibiotics integrated with artificial intelligence, responsive biomaterials, biosensing technologies, and precision targeting systems provide unprecedented opportunities for addressing this growing healthcare crisis. The antimicrobial nanomedicine may represent one of the few truly transformative technological approaches capable of fundamentally altering the future trajectory of global AMR progression. One of the most important advantages of nano-antibiotics in combating AMR is their ability to exert multifunctional antimicrobial mechanisms simultaneously [22]. Conventional antibiotics frequently target single bacterial pathways such as cell wall synthesis, protein translation, or nucleic acid replication, allowing resistant bacteria to evolve specific defense strategies through mutation or gene acquisition. In contrast, many nanoplatforms damage microbial cells through multiple concurrent mechanisms including membrane disruption, reactive oxygen species generation, metabolic interference, intracellular delivery enhancement, and biofilm destabilization. Multifunctional antimicrobial action may conceptually be represented as:

*Resistance Probability* ∝ 1/*Number of Simultaneous Targets*

Because bacteria must adapt to several destructive processes simultaneously, resistance development against multifunctional nanoplatforms may become significantly more difficult. This multi-target capability represents one of the greatest strategic advantages of antimicrobial nanotechnology. Biofilm-associated resistance is another major area where smart nanoplatforms provide substantial therapeutic opportunities [51]. Biofilms create protective microbial communities surrounded by extracellular polymeric matrices that severely restrict antibiotic penetration and promote resistant persister cell survival. Smart nano-antibiotics can penetrate deep into biofilm structures while simultaneously delivering antimicrobial agents, generating ROS, releasing enzymes, or applying photothermal disruption [204]. Stimuli-responsive systems further improve precision by activating therapeutic functions specifically within biofilm microenvironments. The effective biofilm disruption may become critically important because chronic biofilm-associated infections contribute heavily to global antimicrobial treatment failure. Targeted and localized antimicrobial delivery also offers major advantages for AMR management. Conventional systemic antibiotic therapy frequently exposes large microbial populations to suboptimal drug concentrations, thereby accelerating resistance selection. Smart nanocarriers improve infection-site accumulation and maintain controlled therapeutic concentrations directly within infected tissues while minimizing unnecessary systemic exposure [205]. Such precision delivery reduces off-target microbiome disruption and decreases selective pressure on nonpathogenic bacterial populations. The minimizing widespread antibiotic exposure is essential for slowing the ecological expansion of antimicrobial resistance. Artificial intelligence further expands opportunities for combating AMR through predictive therapeutic optimization and rapid resistance surveillance. Machine learning systems can analyze microbial genomics, resistance patterns, patient biomarkers, epidemiological data, and therapeutic outcomes to identify optimal treatment strategies with greater precision. AI-guided nanoplatforms may dynamically adapt drug combinations, release profiles, and targeting behavior according to evolving microbial resistance characteristics [206]. Real-time biosensor monitoring integrated with AI analytics may additionally enable earlier detection of therapeutic failure and emerging resistant strains. The integration of AI with antimicrobial nanotechnology may significantly improve both therapeutic responsiveness and global AMR surveillance capability. Nanotechnology also provides important opportunities for reviving older antibiotics that have lost effectiveness against resistant pathogens. Encapsulation within nanocarriers can improve intracellular penetration, protect unstable drugs from enzymatic degradation, enhance bioavailability, and overcome bacterial efflux mechanisms. Combination nanoplatforms integrating antibiotics with metallic nanoparticles, antimicrobial peptides, or quorum sensing inhibitors further enhance bactericidal synergy [207]. Such strategies may extend the clinical lifespan of existing antibiotics and reduce immediate dependence on entirely new drug classes. The nanotechnology-assisted antibiotic revitalization may provide a practical short-term strategy for managing current resistance crises. Another promising opportunity involves the development of personalized precision antimicrobial therapy. Resistant infections often vary substantially among patients according to pathogen genotype, immune condition, microbiome composition, metabolic state, and infection microenvironment. Smart nano-antibiotics integrated with biosensors and multi-omics analysis may allow individualized therapeutic design optimized for specific patient-pathogen interactions. Such personalized approaches could improve therapeutic efficiency while minimizing unnecessary antibiotic exposure and resistance selection pressure. The individualized antimicrobial therapy may become increasingly important as resistant infections grow more biologically heterogeneous and clinically complex. Global AMR management may additionally benefit from the diagnostic capabilities of advanced nanotechnology systems. Nanobiosensors capable of rapid pathogen identification and resistance gene detection can significantly reduce delays associated with traditional microbiological testing. Early and accurate diagnosis allows clinicians to initiate targeted therapy more quickly while reducing inappropriate empirical antibiotic use [208]. Portable point-of-care nanodiagnostic systems may also improve infectious disease management in resource-limited settings where laboratory infrastructure is limited. The improved diagnostic precision is as important as therapeutic innovation in controlling the spread of antimicrobial resistance. Despite these opportunities, substantial challenges still remain. Manufacturing scalability, regulatory approval, long-term biosafety, economic accessibility, and environmental sustainability continue to limit widespread clinical implementation of advanced antimicrobial nanotechnologies. Resistance adaptation against certain nanomaterials may also eventually emerge if therapeutic misuse occurs. Furthermore, many developing regions facing severe AMR burdens still lack access to advanced healthcare infrastructure required for sophisticated precision nanomedicine. Future success against AMR will require balanced integration of technological innovation, responsible antimicrobial stewardship, global healthcare accessibility, and international collaborative research efforts. Although multifunctional nano-antibiotic systems are designed to reduce the likelihood of resistance development, bacterial adaptation cannot be completely excluded. Potential resistance mechanisms may include alterations in cell membrane composition, enhanced antioxidant defenses against nanoparticle-induced oxidative stress, increased biofilm production, activation of efflux systems, and changes in metabolic pathways that reduce nanomaterial susceptibility. To minimize these risks, future nano-antibiotic platforms should incorporate multiple antimicrobial mechanisms, infection-responsive drug release, and synergistic combinations of antimicrobial agents. Continuous surveillance of microbial adaptation and AI-assisted monitoring of emerging resistance patterns may further support the long-term effectiveness of smart nano-antibiotic therapies against resistant pathogens. Smart nano-antibiotics provide promising opportunities for addressing the growing global AMR crisis. Continued progress in AI-guided nanomedicine, multifunctional therapeutic systems, precision targeting, biosensing integration, and adaptive antimicrobial technologies is expected to significantly expand future therapeutic capabilities. The antimicrobial nanotechnology may ultimately become one of the most powerful scientific tools available for protecting global healthcare systems against the escalating threat of resistant infectious diseases.

## 10. Conclusions

Smart nano-antibiotics have emerged as a promising strategy for addressing the growing challenge of antimicrobial resistance by combining nanotechnology with stimuli-responsive drug delivery, artificial intelligence, biosensing technologies, and precision therapeutic approaches. Compared with conventional antibiotics, these systems offer opportunities for improved drug delivery, controlled release, enhanced antimicrobial activity, and more selective targeting of infection sites. Significant advances have been reported in the development of metallic nanoparticles, polymeric and lipid-based nanocarriers, hybrid nanoplatforms, and AI-assisted formulation optimization. Numerous preclinical studies have demonstrated encouraging results against multidrug-resistant pathogens and biofilm-associated infections. However, it is important to recognize that many smart nano-antibiotic systems, particularly those incorporating artificial intelligence, real-time biosensing, and autonomous therapeutic decision-making, remain at the experimental or early conceptual stage. While AI has shown potential for nanomaterial design, toxicity prediction, and formulation optimization, its application in clinically validated antimicrobial nanomedicine remains limited. Similarly, most multifunctional and stimuli-responsive nanoplatforms have been evaluated primarily in laboratory and preclinical settings, with relatively few systems advancing to clinical investigation. Several challenges continue to hinder clinical translation, including concerns related to long-term safety, biological variability, large-scale manufacturing, regulatory approval, data standardization, and economic feasibility. Addressing these limitations will require coordinated efforts among researchers, clinicians, industry partners, and regulatory agencies. Overall, smart nano-antibiotics represent an important and rapidly developing area of antimicrobial research. Continued advances in material engineering, artificial intelligence, biological validation, and translational development may facilitate the future clinical implementation of safer and more effective antimicrobial nanomedicine, although substantial scientific and regulatory challenges remain to be resolved before widespread clinical adoption can be achieved.

## Figures and Tables

**Table 1:** Comparative Overview of Conventional Antibiotics and Smart Nano-Antibiotic Systems.

Parameter	Conventional Antibiotics	Smart Nano-Antibiotics	Scientific Advantages	References
Therapeutic mechanism	Primarily single-target antimicrobial action such as inhibition of cell wall, protein, or nucleic acid synthesis	Multifunctional mechanisms including membrane disruption, ROS generation, intracellular targeting, and biofilm penetration	Reduced probability of rapid resistance development and improved antimicrobial efficacy	[8,17,19]
Drug delivery behavior	Passive systemic distribution with limited infection-site specificity	Targeted and stimuli-responsive delivery to infected tissues	Enhanced therapeutic concentration and minimized off-target toxicity	[13,19,20]
Biofilm penetration	Poor penetration through dense extracellular polymeric matrix	Enhanced biofilm disruption and deeper penetration capability	Improved eradication of chronic and resistant biofilm-associated infections	[11,17]
Resistance development	High susceptibility to enzymatic degradation, efflux pumps, and target modification	Multiple simultaneous antimicrobial pathways reduce adaptive resistance	Better effectiveness against MDR and XDR pathogens	[4,10,18]
Drug release profile	Rapid and uncontrolled release leading to fluctuating drug concentration	Controlled, sustained, and trigger-responsive release kinetics	Improved therapeutic stability and prolonged antimicrobial action	[19,20,22]
Intracellular infection treatment	Limited intracellular uptake and reduced accumulation in infected cells	Efficient intracellular delivery through nanoscale cellular internalization	Enhanced treatment of persistent intracellular pathogens	[13,23]
Surface functionalization	Minimal capability for ligand-mediated targeting	Easily functionalized with antibodies, peptides, aptamers, or polymers	Improved pathogen targeting and personalized therapy	[15,22]
Toxicity profile	Possible systemic toxicity and microbiota disruption during prolonged therapy	Reduced systemic exposure through localized and controlled release	Lower collateral tissue damage and improved biocompatibility	[12,22]
Responsiveness to microenvironment	No adaptive response to pathological conditions	Dynamically responds to pH, enzymes, ROS, temperature, or external stimuli	Smart therapeutic activation with higher precision	[19,20,21]
Diagnostic integration	Therapeutic function only	Simultaneous diagnostic and therapeutic capability (theranostics)	Real-time infection monitoring and treatment optimization	[23]
AI integration capability	Limited compatibility with computational optimization	Highly compatible with AI-guided design and predictive modeling	Accelerated formulation optimization and precision medicine development	[24,25]
Clinical adaptability	Fixed dosing and generalized treatment approach	Personalized and programmable therapeutic systems	Improved patient-specific antimicrobial management	[23,25]

ROS = Reactive oxygen species; AI = Artificial intelligence. Smart nano-antibiotic systems integrate nanotechnology, stimuli-responsive materials, and computational intelligence to improve antimicrobial precision, therapeutic efficacy, and resistance management.

**Table 2:** Mechanisms of Antimicrobial Action of Smart Nano-Antibiotics.

Mechanism	Biological Target	Antimicrobial Outcome	Example Nanomaterials	Advantages Against AMR	References
Cell Membrane Disruption	Bacterial cell membrane and lipid bilayer	Membrane destabilization, leakage of intracellular components, and rapid bacterial death	Silver nanoparticles, graphene oxide, ZnO nanoparticles, cationic polymeric nanoparticles	Difficult for bacteria to develop resistance due to physical membrane damage	[17,18]
Reactive Oxygen Species (ROS) Generation	Cellular proteins, lipids, DNA, and oxidative defense systems	Oxidative stress-induced bacterial destruction and metabolic dysfunction	ZnO nanoparticles, TiO_2_ nanoparticles, Ag nanoparticles, photothermal nanomaterials	Multi-target oxidative damage reduces adaptive resistance mechanisms	[17,52]
Intracellular Drug Delivery	Intracellular pathogens and infected host cells	Enhanced intracellular antibiotic accumulation and improved pathogen eradication	Liposomes, polymeric nanoparticles, dendrimers	Effective against persistent intracellular bacterial infections	[11,53]
Biofilm Penetration and Disruption	Extracellular polymeric biofilm matrix	Improved penetration, matrix destabilization, and enhanced antibiotic diffusion	Mesoporous silica nanoparticles, metallic nanoparticles, hybrid nanocarriers	Overcomes biofilm-associated antibiotic resistance barriers	[11,17,54]
Controlled and Stimuli-Responsive Drug Release	Infection-associated pathological microenvironment	Site-specific and controlled antimicrobial release under pH, ROS, enzyme, or temperature triggers	pH-responsive polymers, redox-sensitive nanoparticles, thermoresponsive hydrogels	Minimizes premature drug release and improves localized therapy	[19,20,22]
Inhibition of Bacterial Enzymatic Activity	Microbial metabolic enzymes and catalytic proteins	Suppression of essential bacterial biochemical pathways	Metallic nanoparticles and enzyme-responsive systems	Reduced enzymatic resistance and improved therapeutic efficiency	[9,20]
DNA and Genetic Material Damage	Bacterial nucleic acids and replication machinery	Inhibition of replication and induction of bacterial apoptosis-like death	Silver nanoparticles, ROS-generating nanomaterials	Simultaneous genetic and oxidative stress-mediated antimicrobial action	[17,18]
Photothermal Antimicrobial Activity	Bacterial membrane proteins and biofilm structures	Heat-mediated bacterial destruction and enhanced antibiotic sensitivity	Gold nanoparticles, graphene oxide, carbon nanotubes	Effective against multidrug-resistant biofilm-forming bacteria	[21,55]
Immune Modulation and Host Defense Enhancement	Host immune cells and inflammatory pathways	Enhanced immune-mediated bacterial clearance	Biomimetic nanoparticles and multifunctional hybrid systems	Supports host–pathogen defense balance and reduces infection persistence	[23,56]
Synergistic Nano–Drug Combination Therapy	Multiple bacterial survival pathways simultaneously	Enhanced bactericidal efficacy through multimodal therapeutic interaction	Antibiotic-loaded metallic or hybrid nanoparticles	Reduces resistance emergence through combination antimicrobial mechanisms	[57,58]
Quorum Sensing Interference	Bacterial communication signaling pathways	Inhibition of virulence factor production and biofilm maturation	Functionalized polymeric nanoparticles and hybrid systems	Suppresses bacterial pathogenicity without strong selective pressure	[53,59]
Targeted Ligand-Mediated Antimicrobial Delivery	Infection-specific receptors and microbial surface markers	Selective nanoparticle accumulation at infected tissues	Antibody-functionalized nanoparticles, aptamer-modified nanocarriers	Improved precision therapy with reduced systemic toxicity	[22,56]

**Table 3:** Different Types of Stimuli-Responsive Nano-Antibiotic Systems.

Stimulus Type	Trigger Condition	Responsive Mechanism	Drug Release Behavior	Therapeutic Application	Advantages	References
pH-Responsive Systems	Acidic microenvironment in infected tissues and biofilms	Protonation-induced nanoparticle destabilization, polymer swelling, or bond cleavage	Selective release under acidic pathological conditions	Chronic wound infection, intracellular bacterial infection, biofilm-associated diseases	Site-specific delivery, reduced systemic toxicity, enhanced biofilm penetration	[19,20,22]
Enzyme-Responsive Systems	Elevated bacterial enzymes such as proteases, lipases, hyaluronidases, or β-lactamases	Enzymatic degradation of sensitive linkers or polymeric coatings	Infection-triggered antimicrobial activation	Bacterial biofilms, implant-associated infection, localized inflammatory infection	High biological specificity and controlled therapeutic activation	[20,23]
Temperature-Responsive Systems	Local inflammatory hyperthermia or externally applied thermal stimulation	Thermal phase transition, polymer contraction, or heat-triggered carrier destabilization	Temperature-controlled sustained or burst release	Wound healing, orthopedic infection, photothermal antimicrobial therapy	External controllability and combined thermal–antimicrobial activity	[80,55,81,82]
Redox-Responsive Systems	Elevated reactive oxygen species (ROS) or intracellular glutathione imbalance	Cleavage of disulfide, thioketal, or ROS-sensitive chemical bonds	Oxidative stress-mediated intracellular drug release	Intracellular infection, inflammatory tissue infection, resistant biofilms	Infection-selective activation and improved intracellular targeting	[83,84,85,86,87]
Multi-Stimuli Responsive Systems	Simultaneous pathological triggers such as pH, ROS, enzymes, hypoxia, and temperature	Sequential or combined activation of multiple responsive nanocomponents	Hierarchical and programmable therapeutic release	Multidrug-resistant infection, complex biofilm environments, precision antimicrobial therapy	Improved targeting precision, adaptive therapeutic response, enhanced antimicrobial efficacy	[88,89,90,91,92]
Light-Responsive Systems	Near-infrared (NIR), ultraviolet, or visible light irradiation	Photoactivation, photothermal conversion, or photocleavage mechanisms	Spatially and temporally controlled release	Photodynamic antimicrobial therapy and localized infection treatment	Noninvasive remote-controlled activation with precise regulation	[21,55]
Magnetic-Responsive Systems	External magnetic field application	Magnetic guidance and magnetothermal activation	Magnetically controlled drug accumulation and release	Deep tissue infection and implant-associated infection	Enhanced targeting and externally guided therapeutic modulation	[21,81]
Ultrasound-Responsive Systems	Ultrasound-induced cavitation or acoustic stimulation	Mechanical disruption and permeability enhancement	Ultrasound-triggered antibiotic release	Deep-seated infection and biofilm disruption	Noninvasive activation and improved tissue penetration	[21]

**Table 4:** Applications of Artificial Intelligence in Smart Nano-Antibiotic Development.

AI Approach	Function	Nanomedicine Application	Expected Outcomes	Limitations	References
Machine Learning (ML)	Identification of relationships between nanoparticle properties and biological performance	Optimization of nanoparticle size, charge, morphology, surface chemistry, and antimicrobial activity	Accelerated nanomaterial design with improved therapeutic efficiency and reduced experimental screening	Requires large standardized datasets and high-quality experimental input	[92,96]
Deep Learning (DL)	Analysis of highly complex nonlinear biological and physicochemical interactions	Prediction of bacterial membrane interaction, ROS generation, intracellular delivery, and antibiofilm activity	Improved predictive accuracy and discovery of high-performance nano-antibiotic systems	Black-box behavior and limited mechanistic interpretability	[97,98]
Predictive Modeling	Computational prediction of nano–bio interactions and therapeutic outcomes	Protein corona prediction, biodistribution analysis, biofilm penetration, and cellular uptake modeling	Enhanced therapeutic reliability and rational nanoplatform optimization	Biological heterogeneity may reduce universal predictive accuracy	[105,106,107,108]
Toxicity Prediction Models	Prediction of cytotoxicity, oxidative stress, inflammation, and organ accumulation	Safety-oriented nanomaterial screening and biosafety optimization	Reduced developmental cost and improved clinical safety assessment	Incomplete toxicological databases and variability in biological models	[108,110]
QSAR Modeling	Correlation of nanoparticle physicochemical parameters with biological effects	Toxicity estimation and structure–activity relationship analysis	Early-stage identification of hazardous nanomaterial characteristics	Limited applicability across highly diverse nanomaterial systems	[110]
Artificial Neural Networks (ANNs)	Multivariable data processing and therapeutic prediction	Drug loading optimization and controlled release prediction	Improved formulation precision and reproducibility	Computational complexity and overfitting risk	[100,101]
Reinforcement Learning	Iterative optimization based on therapeutic feedback	Adaptive nanoparticle design and dynamic release optimization	Development of self-improving precision nano-antibiotic systems	Requires continuous high-quality feedback data	[103]
Generative AI Models	Autonomous generation of novel nanomaterial architectures	Discovery of optimized antimicrobial nanostructures and multifunctional carriers	Rapid innovation and reduced dependency on empirical trial-and-error methods	Validation and regulatory acceptance remain limited	[98]
AI-Assisted Molecular Simulation	Atomic-scale prediction of nanomaterial interaction with biomolecules	Membrane disruption analysis, drug-carrier interaction, and nanoparticle diffusion studies	Enhanced mechanistic understanding and rational therapeutic engineering	High computational demand and simulation complexity	[98,103]
Multi-Omics Data Integration	Integration of genomics, proteomics, metabolomics, and transcriptomics datasets	Personalized antimicrobial nanotherapy and infection-specific therapeutic optimization	Precision medicine development and patient-specific therapy selection	Data integration complexity and limited standardized omics datasets	[107,108]
AI-Integrated Biosensing Systems	Real-time biological monitoring and therapeutic adjustment	Smart theranostic nanoplatforms and adaptive drug release systems	Dynamic infection monitoring and closed-loop antimicrobial therapy	Technological complexity and translational challenges	[103,108]

**Table 5:** Hybrid Nano-Antibiotic Systems and Their Therapeutic Functions.

Hybrid System	Components	Mechanism	Synergistic Effect	Application	References
Silver Nanoparticle–Antibiotic Hybrid Systems	Ag nanoparticles combined with conventional antibiotics	Membrane destabilization, ROS-mediated bacterial destruction, and enhanced antibiotic internalization	Increased bactericidal potency and restoration of antibiotic sensitivity in resistant strains	MDR bacterial infection and wound-associated infection	[134,52]
ZnO-Based Hybrid Nanoplatforms	Zinc oxide nanoparticles with antimicrobial drugs or polymers	Photocatalytic ROS generation and membrane damage	Improved oxidative antibacterial activity with prolonged therapeutic action	Skin infection, implant coating, and biofilm suppression	[134,136]
Polymeric–Metallic Hybrid Nanocarriers	Biodegradable polymers integrated with metallic nanoparticles	Controlled drug release with simultaneous metallic antimicrobial activity	Enhanced stability, reduced toxicity, and dual antimicrobial functionality	Precision antimicrobial delivery systems	[137,138]
Lipid–Polymer Hybrid Nanoparticles	Lipid shell combined with polymeric core containing antibiotics	Improved encapsulation, sustained release, and enhanced membrane fusion	Increased bioavailability and prolonged circulation time	Intracellular bacterial infection and systemic therapy	[53,139]
Nano–Antibiotic–Peptide Hybrid Systems	Antibiotics integrated with antimicrobial peptides and nanocarriers	Membrane permeabilization and intracellular therapeutic delivery	Synergistic bacterial killing with reduced resistance development	Resistant Gram-positive and Gram-negative infections	[140]
Photothermal Hybrid Nano-Antibiotic Systems	Metallic or carbon nanomaterials with antibiotics	Heat-mediated bacterial destruction and trigger-responsive drug release	Combined thermal ablation and antimicrobial chemotherapy	Biofilm eradication and chronic infection treatment	[141,142]
Magnetic Hybrid Nanoplatforms	Magnetic nanoparticles integrated with antimicrobial agents	Magnetically guided targeting and localized antimicrobial activation	Improved infection-site accumulation and externally controlled therapy	Deep tissue infection and implant-associated infection	[54]
Mesoporous Silica Hybrid Systems	Mesoporous silica nanoparticles loaded with antibiotics and functional coatings	High-capacity drug loading with stimuli-responsive release	Enhanced payload protection and targeted release behavior	Controlled antimicrobial therapy and biofilm penetration	[143]
Carbon-Based Hybrid Nanostructures	Graphene oxide or carbon nanotubes combined with antibiotics	Physical membrane disruption and oxidative bacterial stress	Enhanced surface interaction and multidimensional antimicrobial activity	Resistant bacterial infection and surface disinfection	[59,144]
Biomimetic Hybrid Nanocarriers	Cell membrane-coated nanoparticles carrying antimicrobial agents	Immune evasion and pathogen-targeted delivery	Improved biocompatibility and prolonged circulation	Personalized precision antimicrobial therapy	[56]
Multi-Functional Theranostic Hybrid Systems	Nanocarriers integrated with imaging probes and antimicrobial agents	Simultaneous diagnosis, monitoring, and therapy	Real-time therapeutic tracking with precision treatment capability	Smart infection monitoring and theranostic applications	[145]

**Table 6:** Major Challenges in Clinical Translation of Smart Nano-Antibiotics.

Challenge	Scientific Impact	Clinical Limitation	Possible Solutions	References
Nanomaterial Toxicity	Excessive ROS generation, inflammatory response, cytotoxicity, and long-term tissue accumulation may compromise biosafety	Risk of organ damage, immune dysfunction, and poor patient tolerance during prolonged therapy	Surface engineering, biodegradable nanomaterials, AI-assisted toxicity prediction, and comprehensive biosafety evaluation	[108,110,148]
Physicochemical Stability	Nanoparticle aggregation, premature drug leakage, and structural instability reduce therapeutic reliability	Short shelf life and inconsistent therapeutic performance under physiological conditions	Advanced polymer stabilization, optimized surface coatings, and controlled storage conditions	[83,54]
Large-Scale Manufacturing Complexity	Reproducibility of nanoparticle size, morphology, and drug loading remains difficult during industrial production	Batch-to-batch variability limits regulatory approval and commercial scalability	Standardized synthesis protocols and automated manufacturing technologies	[93,149]
Scalability of Production	Laboratory-scale synthesis methods are often unsuitable for industrial-scale fabrication	High production cost and limited commercial feasibility	Continuous-flow synthesis systems and scalable nanofabrication strategies	[149,150]
AI Data Limitations	Fragmented, heterogeneous, and insufficient datasets reduce predictive reliability of computational models	Reduced clinical confidence in AI-guided therapeutic design	Development of standardized nanomedicine databases and integrated multi-center data sharing	[46,108,144]
Lack of Explainable AI	Black-box machine learning models limit mechanistic understanding and therapeutic transparency	Difficulty in regulatory acceptance and clinical interpretation	Explainable AI frameworks and interpretable predictive modeling approaches	[151,152]
Regulatory Uncertainty	Absence of unified regulatory guidelines for nanomedicine characterization and evaluation	Delayed approval pathways and uncertainty in clinical translation	International regulatory harmonization and nanotechnology-specific evaluation standards	[154,156]
Limited Long-Term Biosafety Data	Insufficient information regarding chronic exposure, biodegradation, and systemic accumulation	Concerns regarding long-term patient safety and post-treatment complications	Longitudinal in vivo studies and real-world clinical monitoring systems	[108,155]
Complex Nano–Bio Interactions	Dynamic interaction with proteins, immune cells, and biological barriers affects therapeutic predictability	Variable biodistribution and inconsistent patient response	AI-assisted predictive nano–bio interaction modeling and precision biomarker analysis	[105,107]
Manufacturing Cost	Advanced nanomaterials, specialized instrumentation, and quality control increase overall production expense	Limited accessibility in low-resource healthcare systems	Cost-effective biomaterials and simplified synthesis technologies	[151,156]
Clinical Reproducibility Issues	Variability in infection microenvironment and patient physiology influences therapeutic behavior	Inconsistent clinical outcomes across patient populations	Personalized nanomedicine design and adaptive therapeutic monitoring	[87,157]
Ethical and Data Security Concerns	AI-driven healthcare systems may involve privacy risks and algorithmic bias	Reduced trust in AI-assisted therapeutic decision-making	Ethical AI governance frameworks and secure biomedical data management systems	[153,158]

**Table 7:** Representative Preclinical and Clinical Studies of Smart Nano-Antibiotic Systems.

Nanoplatform	Antimicrobial Agent	Target Infection	Study Stage	Major Findings	References
Silver nanoparticle-based nano-antibiotic systems	Silver nanoparticles with conventional antibiotics	Multidrug-resistant wound and skin infections	Preclinical	Demonstrated enhanced antibacterial activity, improved biofilm disruption, and reduced bacterial resistance development	[159,160]
Liposomal antibiotic nanocarriers	Vancomycin, gentamicin, and ciprofloxacin	Intracellular and systemic bacterial infections	Preclinical/Clinical	Improved drug stability, prolonged circulation, and enhanced intracellular delivery efficiency	[161,162]
Polymeric nanocarriers	Poly(lactic-co-glycolic acid) (PLGA)-encapsulated antibiotics	Chronic biofilm-associated infections	Preclinical	Sustained drug release and improved therapeutic retention within infected tissues	[163]
ZnO-based antimicrobial nanoplatforms	Zinc oxide nanoparticles	Implant-associated bacterial infection	Preclinical	Significant ROS-mediated antimicrobial activity and biofilm inhibition	[164]
Gold nanoparticle-assisted photothermal systems	Antibiotic-loaded gold nanoparticles	Resistant biofilm infections	Preclinical	Combined photothermal therapy and antimicrobial delivery enhanced bacterial eradication	[165]
Mesoporous silica nanocarriers	Antibiotic-loaded mesoporous silica nanoparticles	Deep tissue and wound infection	Preclinical	High drug loading capacity and stimuli-responsive release improved localized therapy	[166]
Magnetic nanoparticle-guided antimicrobial systems	Magnetic iron oxide nanoparticles with antibiotics	Localized orthopedic and implant infections	Preclinical	Magnetically targeted delivery improved infection-site accumulation and therapeutic precision	[167]
Lipid-polymer hybrid nanoparticles	Hybrid nanocarriers containing antimicrobial drugs	Pulmonary and intracellular infections	Preclinical	Enhanced cellular uptake and reduced systemic toxicity compared with free antibiotics	[168]
Antimicrobial peptide-loaded nanoplatforms	Antimicrobial peptides and nano-antibiotics	Drug-resistant Gram-positive and Gram-negative infections	Preclinical	Improved membrane disruption and synergistic bactericidal activity	[169]
Biomimetic cell membrane-coated nanoparticles	Membrane-coated nano-antibiotic systems	Immune-evasive bacterial infections	Preclinical	Prolonged circulation and enhanced pathogen-targeted delivery	[170]
Stimuli-responsive smart nanocarriers	pH- and ROS-responsive antibiotic nanoparticles	Biofilm-associated chronic infections	Preclinical	Controlled infection-triggered release increased therapeutic specificity	[171]
AI-optimized nano-antibiotic formulations	Computationally designed antimicrobial nanoplatforms	Personalized precision antimicrobial therapy	Early translational research	AI-assisted optimization improved formulation efficiency, release kinetics, and biosafety prediction	[172,173]
Theranostic antimicrobial nanoplatforms	Imaging-integrated nano-antibiotic systems	Real-time monitored infection therapy	Preclinical	Simultaneous diagnosis and antimicrobial treatment improved therapeutic monitoring capability	[174]

## Data Availability

No new data were created or analyzed in this study. Data Sharing is not applicable.
